# Influences of Double Versus Snaffle Bridles on Equine Behaviour at Dressage Competitions and Factors That Interact with Their Effect

**DOI:** 10.3390/ani15121782

**Published:** 2025-06-17

**Authors:** Rifka Faithfull, Kate Lewis, Emily Drury, Sebastian McBride

**Affiliations:** 1 Department of Life Sciences, Aberystwyth University, Aberystwyth SY23 3FG, UK; rifkafaithfull@gmail.com; 2 Centre for Comparative and Evolutionary Psychology, University of Portsmouth, Portsmouth PO1 2DY, UK; kate.lewis@port.ac.uk; 3 School of Veterinary Science, Aberystwyth University, Aberystwyth SY23 3AH, UK; emd37@aber.ac.uk

**Keywords:** horse, conflict behaviours, bridle, rider experience, competition, tack

## Abstract

Despite welfare concerns, conflict behaviours (indicative of momentary states of acute stress) are common in horses during dressage competitions. Identifying factors that affect conflict behaviours during competition therefore has the potential to improve equine welfare. The aim of this exploratory study was to assess the effect of bridle type (double versus snaffle) in relation to other potential affecting factors (e.g., average head angle, competition level and type, tack variation) on conflict behaviours during dressage competition. An ethogram was created to analyse the conflict behaviour of horses (*n* = 135) at dressage competitions. Behaviours were reduced into nine components using principal component analysis. The effects of bridle type and its interaction with other additional factors (competition level, competition type, average head angle) on component scores were analysed using multilevel Bayesian regression models. The results demonstrated a multifaceted interaction between bridle type, inferred rider skill level, and ridden head angle. The results also demonstrated that during dressage tests, 100% of horses performed the conflict behaviours of mouth opening and 86.7% of horses performed tail swishing. The ethological and welfare implications of these findings are discussed.

## Simple Summary

Public concern around the use of different equipment items on horses, and their implications for horse welfare, has led to growing research in this area. The effects of bridle type (double versus snaffle) on equine behaviour has yet to be investigated. This study assessed the effect of wearing double versus snaffle bridles, in conjunction with other factors, including competition level, competition type, and the average angle of the horse’s head whilst ridden, on the presence of conflict behaviours during dressage competitions. The results demonstrated that conflict behaviours were consistently present in the majority of horses during the observed dressage tests and suggested a complex interaction between bridle type and the inferred rider skill level as well as the horse’s ridden head angle during the dressage test. Further research is needed to clarify this complex relationship.

## 1. Introduction

The welfare of performance horses in sport is increasingly being called into question, both in the media and within scientific studies [1,2,3,4,5]. The Equine Ethics and Wellbeing Commission [6] provided the FEI with recommendations on areas in which to enact change. They highlighted ‘training and riding, tack and equipment’ as their highest priority area, with the largest public concerns surrounding the use of bits (41%), excessive tightening and harsh types of nosebands (20%), and the mandatory use of spurs (18%) [6]. The FEI’s response has led to spurs becoming optional at all levels of the sport as of January 2024, as well as the introduction of a noseband gauge to standardise the measurement of noseband tightness in competition and therefore prevent over-tightening.

Other aspects of ‘training and riding, tack and equipment’ may be of concern from a welfare perspective due to the high prevalence of conflict behaviours seen in the ridden competing horse [7,8,9,10,11]. Conflict behaviours are defined here as behaviours performed by the ridden horse that indicate momentary or ongoing states of acute stress. These states may arise from both physical factors (such as pain or discomfort), psychological factors (such as reluctance or inability to respond to operant cues (aids)), or a combination of the two. Typical examples of conflict behaviours range from full body behaviours, such as tail swishing and tripping, to facial behaviours, including exposure of the sclera or the tongue. Conflict behaviours in the ridden horse are often non-specific [12]. For example, tail swishing is prevalent during situations of high physical demand [7], during the use of the spur [13], or because the animal is experiencing pain [14]. Tail swishing can also occur as a non-conflict behaviour in responses to the presence of flies or as a form of communication to indicate an imminent agonistic interaction, such as a kicking or biting [15]. The context surrounding the execution of the conflict behaviour is therefore highly important to consider, as not all instances of the behaviour will be indicative of momentary or ongoing states of acute stress. Conflict behaviours have been observed at high levels across a wide range of competing horses [7,8,9,10] (e.g., in 97.6% of analysed dressage test movements (left- and right-hand circles, change of rein, upwards and downwards transitions, and halt) [10]). Mouth-related conflict behaviours, in particular, have been reported as one of the most prevalent conflict behaviours [16,17] (e.g., performed by 68% of horses [16]). This suggests that there may be a lack of awareness on the part of both riders and trainers as to when this behaviour is occurring and/or a lack of awareness that it is indicative of a negative emotional state in the horse.

Within the equestrian discipline of dressage, research into factors leading to conflict behaviours has previously focused on hyperflexion [8,9,18] and incorrect use of bits [19,20,21]. Whilst the effect of bridle type (double [two bit] versus snaffle [one bit]) on rein tension and pressure beneath the headpiece and the noseband has previously been investigated [22], its effect on conflict behaviours is yet to be assessed, particularly in relation to contextual factors, such as the level and type of dressage competition, the horse’s head angle when ridden, and the use of different tack items, such as the noseband, bit cheekpiece (of the snaffle/bridoon), spurs, and ear bonnets. The inclusion of these factors into multi-level model analyses has the opportunity to provide a more complete picture as to why conflict behaviours may be arising. The aim of this study, therefore, was to provide an exploratory investigation into the effects of double and snaffle bridles on equine behaviour at dressage competitions in the context of other potentially influencing factors (average head angle, competition level, competition type, rider gender, ear bonnet, cheekpiece type, noseband type, patting frequency, and test score) in order to identify future directions for research into the use of different bridle types in the competition dressage horse from a welfare perspective.

## 2. Materials and Methods

### 2.1. Data Collection

Dressage tests (*n* = 135) were filmed [10] by the investigator at two British Dressage competitions from elementary to intermediate I (inter 1) levels. The use of double bridles is currently mandatory at CDI3* level and above for international competitions; therefore, double bridles have become increasingly more common than snaffle bridles at higher competition levels. As such, there were only two snaffle bridles at PSG level and one at inter 1 level, whereas lower levels had greater numbers of snaffles. Category sizes for bridle types across competition levels for each model are presented in the results, and the overall numbers for all horses are presented in Table 1. As such, whilst these data are included within the Bayesian regression models, during discussion of the results, the interactions between bridle type and level are only considered from elementary to advanced medium levels.

**Figure 1:**
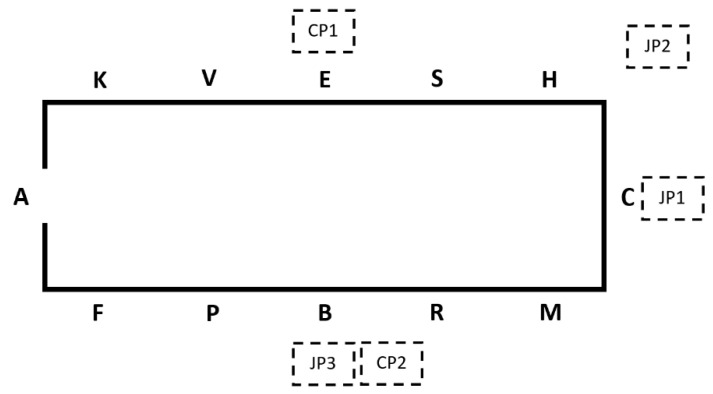
A dressage arena showing the positioning of the judges (JP) and the position of the cameras (CP). At the regional competition, judges were positioned at JP1, JP2, and JP3, and cameras were positioned at either CP1 or CP2. At the regional competition, CP2 was positioned inside of a judges’ box. At the typical competition, there was a single judge at JP1, and the camera was at CP2. The camera’s tripod was levelled via spirit level, and the height adjusted so that the whole horse and the rider were always visible. Cameras were panned and zoomed to keep the horse as large as possible in the frame to maintain the highest possible resolution. The cameras were able to rotate 360° so that their field of view covered the whole arena.

The camera (Canon EOS 700D, EF-S 18–55 mm, 30 f/s, 1920 × 1088, Canon, Tokyo Japan) was positioned as in Figure 1. The positioning of the camera on the long side of the arena was chosen to minimise the amount of the test in which parts of the horse would not be visible to the camera. Tests were recorded from when the horse entered the arena to when they left. Test scores were recorded, and 28 competitors completed a questionnaire detailing the horse, its tack, the rider, and the training experience of the horse and the rider (full questionnaire presented in the Supplementary Materials). The questionnaire was completed by the competitor either as an online form or as a paper copy after they completed the dressage test. Informed consent was obtained from all subjects involved in the study.

### 2.2. Behaviour Scoring

A table of ridden conflict behaviours was initially created based upon the behaviours listed in the Ridden Horse Pain Ethogram [23] (Table 2). During initial observations of the footage collected, however, it was found that some of the behaviours were either not discernible from the footage (e.g., intense stare) or not easily identifiable (e.g., bilateral hind toe drag) and therefore were removed. Some conflict behaviours additional to those listed in the Ridden Horse Pain Ethogram [23] were observed and were thus added to the list of recorded conflict behaviours. These were lip smacking [24], head tossed or lifted out of frame [8,9], and jaw twisting [9,25]. Lip smacking has not previously been defined as a conflict behaviour, but its occurrence has been positively correlated with other conflict behaviours [26]; thus, it was included in this instance. Incorrect canter lead [23] has also not previously been defined as a conflict behaviour; however, it is a result of circumstances previously included as conflict behaviours (e.g., un-cued behaviour [10]), and it can arise from indistinguishable rider aids or incorrect use of negative reinforcement [27] and therefore was also included in this instance. All behaviours were defined and given specific criteria for their performance (Table 2).

For the duration of the dressage test, behaviours were recorded as transient events (average number of occurrences of the behavioural event per minute) or continuous states (percentage time of dressage test performing the behaviour). Some behaviours were recorded as both an event and a state (see Table 2). A video was created with examples of some conflict behaviours to support their ethogram definitions, and it is available at https://youtu.be/3P_U86MvvjI (accessed on 13 May 2025).

The behavioural scoring began when the first hoof entered the arena and ended when the rider finished saluting. Videos were scored by viewing them on a computer. Tests were paused throughout to accurately record each behaviour and, where necessary, sections of tests were rewatched multiple times to ensure accurate scoring. Where there was a particularly high prevalence of specific behaviours, the test was rewatched in its entirety to count high volume behaviours individually and then score other behaviours to ensure accuracy and that high volume behaviours did not obscure the scoring of other behaviours. A subset of ten randomly selected videos was scored by a second blinded observer to test for unconscious bias in the original observations.

**Figure 2:**
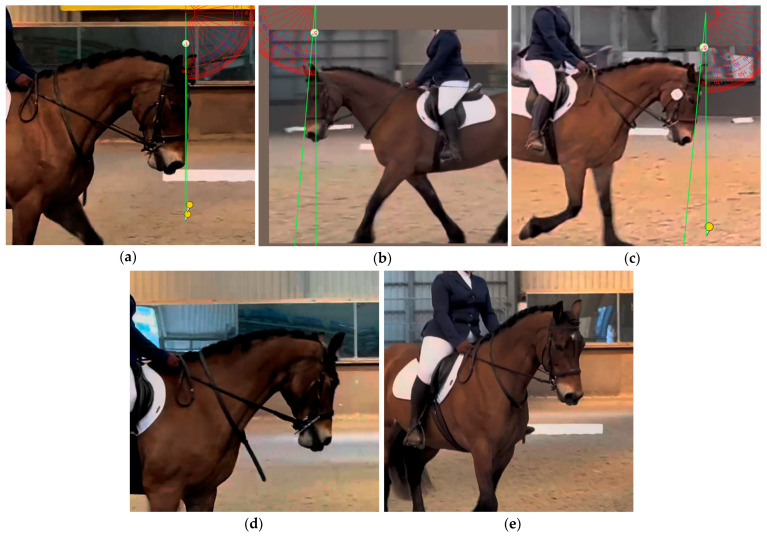
(**a**) Angle measurement of the head from the poll to the nasal plane using online protractor software [28] measuring at 1°. (**b**) An example of a positive head angle that measures at 5°. (**c**) An example of a negative head angle that measures at −5°. (**d**) Any amount of rotation away from the camera distorted the measurement of the angle of the nasal plane more than 5%, and therefore this image is unsuitable for angle measurement. (**e**) This horse is rotated towards the camera at an angle of more than 15°, which distorts its measurement by more than 5% and therefore is unsuitable to be used for angle measurement.

The angle of the nasal plane was measured to the nearest degree throughout the video using online protractor software [28], as in Figure 2. Angle measurements were collected from images taken at 10 s intervals, omitting any images that were not accurately measurable (on average, 38% of the images were suitable for measurement) (Figure 2) for the duration of the test. In the instance that images at 10 s intervals did not provide enough viable images, images were taken at 5 s intervals. The rotation of the horse influenced the accuracy of measuring the head angle, with an angle of rotation towards the camera of more than 15° and any amount of rotation away from the camera distorting the measurement of the head angle by more than 5% (Figure 2) and therefore being unsuitable to measure. All nasal plane measurements were taken with the head angle between the withers and the poll (in relation to the vertical) between 69 and 92° (measurement calculated using angles during a trot). An average angle for each horse was calculated. The percentage of the test spent with the head in angle ranges of 5° intervals ranging from 20° to −20° was calculated from the angle measurements. These were later condensed into two groups of the percentage of the test where the horse’s head was at angles of 0° to −10° and more than −10°. Only the average head angles were included in the initial Bayesian regression models (BRMs) in order to reduce the number of variables within the models and avoid a lack of independence between the angle categories affecting the model’s fit. The condensed angle categories were later used in a separate analysis using BRMs, as only the average angle was suitable to use in the whole dataset’s BRMs.

The competition level, bridle type, competition type/location (indoor/outdoor), rider’s gender, presence of spurs or ear bonnets, cheekpiece on the snaffle/bridoon bit, type of noseband, and any elimination/retirements that occurred were recorded as categorical variables (Table 3). The questionnaire variables that are included within the discussion are detailed in Table 3; additional questionnaire results and discussion are located within the Supplementary Materials, Table S1.

### 2.3. Statistical Analysis

A Principal Component Analysis (PCA) with varimax rotation (*n* = 135) was conducted using SPSS (v29.0.1.1, IBM Statistics, Armonk, NY, USA) to reduce the behaviour results into weighted components and scores, ensuring the determinant value was >0.00001, Kaiser–Meyer–Olkin (KMO) was >0.5, and Barlett’s test was <0.05. The weighted behaviours for each component were chosen based on an eigen value > 0.3 as the minimum component loading from the rotated component matrix. This generated a component score for each individual, for each component.

All remaining analyses were conducted in R v. 4.4.1 (v. 4.4.1, R core team, Vienna, Austria). Multilevel models were fitted within a Bayesian framework. Bayesian regressions return a distribution of possible effect sizes rather than point estimates (as is the case in frequentist regression), with the credible interval (CrI) being the range of this distribution containing a particular percentage of probable values. In this case, we used a 95% CrI. Where a CrI does not contain zero, this suggests an effect is evident.

The data and R scripts used in this study are available at https://doi.org/10.17605/OSF.IO/Y2DCH.

#### 2.3.1. Whole Dataset Analyses

Pearson correlation coefficients between pairs of all continuous predictor variables, and Cramer’s V coefficients between pairs of all categorical predictor variables, were calculated. Where these exceeded 0.80, only one of the variables was included in the models to avoid multicollinearity [29]. As a result, the competition location was removed from analyses due to a strong association with the competition type (Cramer’s V = 1.0). Bridle type and noseband type were similarly strongly associated (Cramer’s V = 0.87). Given the importance of bridle type for our aims, its inclusion was deemed of higher importance; thus, it was noseband type that was not included in analyses. Visual examination of box plots generated from all possible pairs of continuous and categorical variables did not reveal any associations.

A series of Bayesian regression models (BRMs) were fitted using the brm function in the brms package [30,31], with one for each component identified through the PCA, bar component 9. Component 9 only had one weighted behaviour, kicking, which was performed in one instance by a single horse and thus was omitted as it was not informative of behaviours of the whole sample. For each model, the dependent variable was the component of interest. Independent variables were all other variables collected, namely, bridle type, average head angle, competition level, competition type, rider’s gender, ear bonnet, cheekpiece type, patting frequency, and test score. We were also interested in how the interaction between bridle type and the other variables predicted each component. It was not possible to test for all potential interactions, as models would not converge with such a large number of predictors. We therefore selected the three factors of highest interest, namely, average head angle, competition level, and competition type, in which to examine the interaction with bridle type.

BRMs were fitted with a Gaussian distribution, default priors, a maximum tree depth of 10, and an adapt delta of 0.8. To ensure that Gaussian distribution was the best fit for the data, initially, models were also fitted with a Poisson distribution; however, Gaussian models were consistently a better fit for the data. More information about model diagnostics can be found in the Supplementary Material. Each BRM was run for two chains of 10,000 iterations, discarding the first 500 as warmup. Statistical inference was determined by examining whether the 95% CrI of the population-level effect for each predictor in a given model overlapped with zero or not. If less than 10% of the total difference between upper and lower CrI was above or below zero, the association was classified as a trend. For categorical variables, the hypothesis() function in the brms package was used to generate comparisons between pairs of groups not included in the standard model output summary. CrIs for pair-wise comparisons are of the distribution of the difference of the means of the two groups, and statistical inference was determined in the same way as population-level effects, described above. Conditional effects plots were generated in brms and visualised with ggplot2 [32]. These display model-predicted coefficients with 95% CrIs.

#### 2.3.2. Questionnaire Data

Due to the limited size of this dataset, it was not possible to generate complex multi-level models that included all possible predictors. Instead, for each component, we ran a series of models, with one for each of the questionnaire predictors that was suitable for analysis (namely, professional bridle fit, rider maintenance treatments, time spent training in a double bridle, length of time since starting to wear a double bridle, the rider’s highest level trained at home, the horse’s highest level trained at home, the length of time the horse spent training before first competing, time since the horse’s last dental examination, bit category, and the frequency of dental examinations). BRMs were fitted using the same configuration as full dataset models. In each model, we initially included any variables found to predict, or trend to predict, the component of interest in full dataset analyses, plus an interaction effect between bridle type and the questionnaire variable being examined. In cases where this initial model would not converge, the interaction term was removed, and the model was re-run. If this also failed to converge, predictors were systematically removed until the model did converge. As bit category was found to be strongly associated with bridle type (Cramer’s V = 1.0), bridle type was not included in bit category models. Statistical inference was derived as described above, as were outcomes of the remaining pair-wise comparisons.

Only the questionnaire results that are pertinent to results in the main dataset and provide additional context surrounding the potential effects of bridle type are presented in this study. The remaining questionnaire results and their corresponding discussion can be found in the Supplementary Materials (Tables S2–S9).

#### 2.3.3. Exploratory Analyses of Head Angle Effects

As average head angle was found to predict component 4 and hyperflexion has been found to be detrimental to the horse and associated with conflict behaviours [8,9,11,18,33,34,35], we decided to run exploratory analyses to examine the effect of negative head angles on each component. To reduce the number of 0 s in the data, negative head angle groups were combined into two larger groups: <−10° and −10–0°. We then ran BRMs, one for each component, using the methods described previously. Independent variables were the two angle categories and interactions between the angle categories and the bridle type, plus any variables found to predict, or trend to predict, the component of interest in full dataset analyses.

#### 2.3.4. Exploratory Analyses of Noseband Types

As it was not possible to include noseband types in the whole dataset analysis due to its high correlation with bridle type, additional BRMs were run for components 4, 6, and 7 (components for which bridle type was predictive) to identify any variation based on noseband type within snaffle wearers (as all double bridles were worn with a cavesson noseband) to help establish if noseband types could possibly contribute to these predictive bridle type results. These models were fitted using the same variables as bridle type models, replacing bridle type with noseband type, and with only one interaction term (noseband type * average head angle), as models would not converge with three interaction terms.

#### 2.3.5. Interpretation of BRM Results

Reported for each predictive result are the estimate (Est.) and the 95% credible intervals (CrIs). The estimate is the mean of a given variable’s posterior distribution, and it provides an estimate of the overall effect of the variable. Positive values denote a positive effect, and negative values denote a negative effect. The two-sided CrIs are based on the quartiles of this posterior distribution. We can be 95% certain that the standard deviation of the posterior mean falls within this interval. Thus, where a CrI does not span 0 (i.e., upper and lower CrIs are both positive or both negative), this suggests that an effect is evident, and thus the result is termed ‘predictive’. If less than 10% of the total difference between upper and lower CrIs was above or below zero, the result was classed as a ‘trend’ or ‘trended towards being predictive’. Estimates and credible intervals for all results, including non-predictive results, are presented in the Supplementary Materials (Tables S8–S10).

Interaction results are denoted by an asterisk (*) between the two interacting variables. In the case of defining which categories are interacting within these variables, an asterisk is used between the two categories (e.g., double*medium would denote double bridle wearers at medium level; in the case of describing these categories’ sizes, the category initials are used, e.g., D*M).

#### 2.3.6. Inter-Observer Scoring

Inter-observer scoring compared the raw behavioural scores of a random selection of 10 horses with scoring by a secondary blinded observer. This was analysed using SPSS (v29.0.1.1, IBM Statistics, Armonk, NY, USA). Hypothesis testing was carried out on the raw behavioural scores to assess suitability for parametric testing. Inter-observer scoring between the original behavioural scores and those of the secondary observer was tested for concordance with Kendall’s W test for each behaviour to assess if any behaviours were scored differently by the two observers. Behavioural scoring was compared between observers using a paired sample *t*-test for all behavioural scores for all horses together and all behavioural scores for the two bridle types separately to assess if there was a significant difference in behavioural scoring between observers for either bridle type. Individual behaviours were also compared between observers to assess if any behaviours were scored significantly differently by either observer. A ‘scoring difference’ was calculated by subtracting the secondary observer behaviour scores from the original observer behaviour scores. The scoring difference was compared between double bridle and snaffle bridle wearers using an independent *t*-test to test for bias in the original scoring to ensure that the original observer scores were not higher for one bridle type than the other when compared to the scoring of the secondary blinded observer.

## 3. Results

### 3.1. Behavioural Descriptions

The percentage of horses performing and the average performance for each behaviour is presented in Table 4. On average, horses spent 27.4% of their test performing conflict state behaviours (standard error of the mean: 1.97, range: 0.00–97.47). Mouth opening was the most common conflict behaviour performed during the test, with 100% of horses performing it as an event and 95% as a state, followed by tail swishing, which was performed by 87% of horses during the test. Mouth opening occurred on average once every 5 s (ranging from 0.2 to 67 times per minute), and, on average, horses spent 22% (ranging from 0 to 97%) of their test in a mouth open state. Tail swishing occurred on average every 8 s (ranging from 0 to 53 times per minute). Kicking only occurred once for a single horse.

### 3.2. Principal Component Analysis

The determinant (0.095), KMO (0.505), and Bartlett’s Test (298.464, df = 171, *p* < 0.001) met the assumptions of the Principal Component Analysis. Table 5 shows component loadings for the nine components extracted from the conflict behaviour data, highlighting eigen values > ±0.3. Table 6 describes each component, with its component name, the weighted behaviours (positive and negative) with eigen value > ±0.3, and the percentage of the total variance explained by each component.

### 3.3. Bayesian Regression Models

#### 3.3.1. Component 1—Mouth-Related Conflict Behaviours 1

All predictive and trend results from the main dataset for component 1 are presented in Table 7, as well as relevant questionnaire results. Additional questionnaire results are presented in the Supplementary Materials (Table S2).

**Figure 3:**
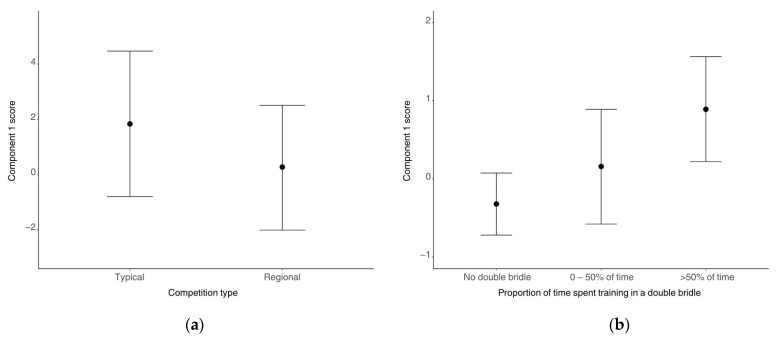
(**a**) Component 1 (mouth-related conflict behaviours 1) scores at typical (*n* = 11 horses) and regional (*n* = 107 horses) competition types. Higher component 1 scores were predicted at the typical competition than at the regional competition. (**b**) Component 1 scores for the proportion of training horses spent wearing a double bridle (*n*: = no double bridle = 17, 0–50% of time = 5, >50% of time = 6). Higher component 1 scores were predicted when the double bridle was worn for more than half of training rides compared to horses that did not wear a double bridle. There was a trend towards higher component 1 scores being predicted when horses wore a double bridle for more than half of training rides compared to those who wore a double bridle for less than half of training rides.

Competition type predicted component 1 scores (Est. = −1.57, CrIs [−3.03, −0.08]). Component 1 scores were lower at regional competitions than typical competitions (Figure 3). Competition level predicted component 1 with greater component 1 scores at inter 1 (Est. = 1.31, CrIs [0.47, 2.15]), and PSG (Est. = −1.02, Crls [−1.83, −0.23]) levels than at medium level, as well as trends towards higher component 1 scores at advanced medium (Est. = −1.19, CrIs [−2.48, 0.07]) than at medium and inter 1 than elementary (Est. = −0.63, CrIs [−1.34, 0.08]). The interaction between bridle type and competition level was predictive of component 1. When wearing a snaffle bridle, there were higher component 1 scores at medium (Est. = −1.16, CrIs [−2.18, −0.15]) and PSG (Est. = −2.47, CrIs [−4.44, −0.50]) than at elementary and higher component 1 scores at PSG than at inter 1 (Est. = −3.06, CrIs [−5.87, −0.21]). With a snaffle bridle, there were also trends towards higher component 1 scores at PSG than at advanced medium (Est. = 2.51, CrIs [−0.18, 5.20]) and at medium than inter 1 (Est. = −1.75, CrIs [−3.80, 0.31]). There was a trend towards time in the <−10° angle group predicting component 1 with a negative relationship (Est. = 0.0106, CrIs [−0.0025, 0.0238]).

The amount of time spent training in a double bridle predicted component 1, with higher component 1 scores for horses ridden in double bridles for more than half of training rides compared to horses who did not wear double bridles (Est. = 1.21, CrIs [0.42, 2.00]). There was also a trend towards horses who wore a double bridle for more than half of training rides having a higher component 1 score than those who wore a double bridle for less than half of training rides (Est. = −0.73, CrIs [−1.55, 0.09]) (Figure 3). There was a trend towards higher component 1 scores when riders received regular maintenance treatments (Est. = 0.67, CrIs [−0.04, 1.38]). No other variables in the main dataset models predicted component 1 (Table S10).

#### 3.3.2. Component 2—Full Body Conflict Behaviours 1

All predictive and trend results from the main dataset for component 2 are presented in Table 8. Additional questionnaire results are presented in the Supplementary Materials (Table S3).

The interaction between bridle type and competition level predicted component 2 scores. When wearing a snaffle bridle, inter 1 had higher component 2 scores than elementary (Est. = −0.90, CrIs [−1.80, −0.01]) and medium level (Est. = 1.16, CrIs [0.24, 2.09]), and inter 1 trended towards being higher than advanced medium (Est. = 1.17, CrIs [−0.03, 2.36]). These differences were not seen in horses wearing double bridles. There was a trend towards horses not wearing an ear bonnet having higher component 2 scores than those who wore an ear bonnet (Est. = −0.16, CrIs [0.34, 0.02]). There was also a trend towards a negative correlation between test scores and component 2 scores, with test scores being lower with higher component 2 scores (Est. = 0.03, CrIs [0.07, 0.02]).

No other variables in the main dataset models predicted component 2 scores (Table S11).

#### 3.3.3. Component 3—Mouth-Related Conflict Behaviours 2

No main dataset variables were predictive of component 3 scores (Table S12). The predictive and trend results from the questionnaire models are presented in the Supplementary Material (Table S4).

#### 3.3.4. Component 4—Full Body Conflict Behaviours 2

All predictive and trend results from the main dataset for component 4 scores are presented in Table 9. Additional questionnaire results are presented in the Supplementary Materials (Table S5).

**Figure 4:**
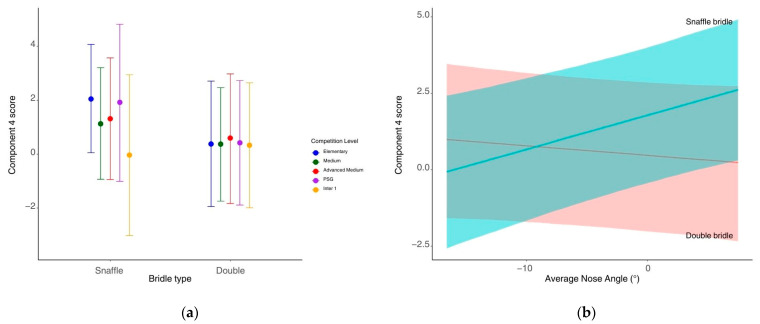
(**a**) The interaction between bridle type and competition level was predictive of component 4 (full body conflict behaviours 2) (*n* =: D*E = 11, S*E = 39, D*M = 9 S*M = 15, D*A = 4, S*A = 4, D*P = 20, S*P = 1, D*I = 14, S*I = 1; D = double bridle, S = snaffle bridle, E = elementary, M = medium, A = advanced medium, P = Prix St. George, I = intermediate I; * denotes category interaction; Colours: blue = elementary; green = medium, red = advanced medium, purple = Prix St. George, yellow = intermediate I). Component 4 scores were higher at elementary level than medium when wearing a snaffle bridle. (**b**) Component 4 scores show a different relationship with average head angle between bridle types (*n* =: S = 60, D = 58; Colours: blue = snaffle bridle, red = double bridle). There is a slight negative relationship for double bridles, whereas there is a positive relationship for snaffle bridles.

The interaction between bridle type and the average angle of the horse’s head predicted component 4 (Est. = 0.14, CrIs [0.05, 0.24]) (Figure 4). There was a positive relationship between head angle and component 4 for those wearing a snaffle bridle, whereas this relationship was negative for those wearing a double bridle. The interaction between bridle type and competition level predicted component 4 scores (Figure 4), with higher component 4 scores at elementary than at medium levels (Est. = 0.91, CrIs [0, 1.82]) or inter 1 level (Est. = 2.05, CrIs [0.22, 3.87]) when the horse was wearing a snaffle bridle. These differences between competition level were not seen in horses wearing double bridles (Table S4). There was a trend towards the number of times the horse was patted at the end of the test predicting component 4 scores, with those who were patted more having a higher component 4 score (Est. = 0.10, CrIs [−0.00, 0.03]). There was an interaction between the <−10° angle group and bridle type being predictive of component 4 scores (Est. = −0.0279, CrIs [−0.0502, −0.0057]) with a negative relationship between the <−10° angle group and component 4 for snaffle bridles and a slight positive relationship for double bridles.

No other variables in the main dataset models predicted component 4 scores (Table S13).

#### 3.3.5. Component 5—Full Body Conflict Behaviours 3

All predictive and trend results from the main dataset for component 5 scores are presented in Table 10. Additional questionnaire results are presented in the Supplementary Material (Table S6).

Component 5 scores were higher at advanced medium level than at other levels (A-E (Est. = −1.64, CrIs [−2.83, −0.45]), A-I (Est. = −1.39, CrIs [−2.53, −0.28]), A-M (Est. = −1.72, CrIs [−2.95, −0.53]), A-P (Est. = −1.22, CrIs [−2.31, −0.13])). The interaction between bridle type and competition level predicted component 5 scores. Horses at PSG level trended towards greater component 5 scores than those at advanced medium when wearing a snaffle bridle (Est. = 2.75, CrIs [0.20, 5.27]). There were trends towards higher component 5 scores for horses at advanced medium level than elementary (Est. = 1.36, CrIs [−0.17, 2.89]) or medium (Est. = 1.51, CrIs [−0.10, 3.10]) when wearing a double bridle. There was a trend towards the interaction between bridle type and competition type predicting component 5 scores, with higher component 5 scores at regional competitions when horses were wearing a double bridle (Est. = −1.32, CrIs [−2.87, 0.23]). There was a positive trend towards time in the <−10° angle group predicting higher component 5 scores (Est. = 0.0106, CrIs [−0.0025, 0.0238]).

No other variables in the main dataset models predicted component 5 scores (Table S14).

#### 3.3.6. Component 6—Training-Related Conflict Behaviours

All predictive and trend results from the main dataset for component 6 scores are presented in Table 11, as well as relevant questionnaire results. Additional questionnaire results are presented in the Supplementary Materials (Table S5).

**Figure 5:**
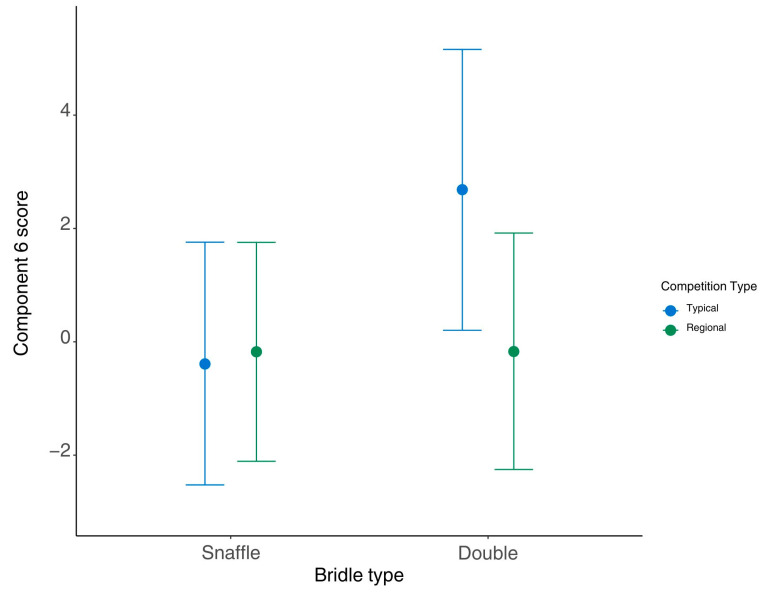
The interaction between bridle type and competition type was predictive of component 6 (training-related conflict behaviours) (*n* =: S*T = 8 D*T = 3 S*R = 52 D*R = 55; S = snaffle bridle, D = double bridle, T = typical competition, R = regional competition; * denotes category interaction; Colours: blue = typical competition, green = regional competition). Higher component 6 scores were predicted at the typical competition when wearing a double bridle.

Bridle type predicted higher component 6 scores with a double bridle than a snaffle bridle (Est. = −2.52, CrIs [−4.64, −0.38]). Competition level predicted component 6 scores, with higher component 6 scores at inter 1 than medium (Est. = 1.00, CrIs [0.19, 1.79]) and PSG levels (Est. = 0.73, CrIs [0.34, 0.16]). The interaction between bridle type and competition level trended towards predicting component 6 scores. Advanced medium level had higher scores than inter 1 when wearing a snaffle bridle (Est. = −2.25, CrIs [−4.81, 0.31]), inter 1 had higher component 6 scores than PSG level, and there was a trend towards medium having higher component 6 scores than inter 1 when wearing a snaffle bridle (Est. = −1.93, CrIs [−3.92, 0.06]). Competition type predicted higher component 6 scores at the typical competition (Est. = −2.86, CrIs [−4.29, −1.43]). The interaction between bridle type and competition type predicted component 6 scores, with higher component 6 scores at typical competitions than regionals when the horse was wearing a double bridle (Est. = 1.43, CrIs [4.68, 3.07]) (Figure 5). There was a trend towards horses with male riders having higher component 6 scores (Est. = 0.47, CrIs [−0.08, 1.01]). The cheekpiece type of the snaffle bit or bridoon bit within the double bridle predicted component 6 scores. Horses who wore bits with hanging cheek cheekpieces had higher component 6 scores than those who wore loose ring bits (Est. = 2.25, CrIs [0.46, 4.05]). There was a trend towards horses with a hanging cheek cheekpiece having higher component 6 scores than those with a D-ring (Est. = 2.43, CrIs [−0.40, 5.23]). There was a trend towards time in the <−10° angle group predicting component 6 with a negative relationship (Est. = −0.0114, CrIs [−0.0255, 0.0027]).

The amount of time horses spent training in double bridles predicted component 6 scores, with those training in a double bridle for more than half of training rides having higher component 6 scores than those who did not wear a double bridle (Est. = 1.29, CrIs [0.15, 2.41]).

No other variables in the main dataset models predicted component 6 (Table S15).

#### 3.3.7. Component 7—Spook-Related Conflict Behaviours

All predictive and trend results from the main dataset for component 7 scores are presented in Table 12. Additional questionnaire results are presented in the Supplementary Materials (Table S8).

The interaction between bridle type and competition type predicted component 7 scores, with higher component 7 scores at typical competitions than regional competitions when wearing a snaffle bridle (Est. = −1.95, CrIs [−3.60, −0.30]). There was a very strong trend of bridle type predicting component 7, with snaffles having higher component 7 scores (Est. = 2.19, CrIs [−0.01, 4.37]).

No other variables in the main dataset models predicted component 7 scores (Table S16).

#### 3.3.8. Component 8—Full Body Conflict Behaviours 4

All predictive and trend results from the main dataset for component 8 scores are presented in Table 13. Additional questionnaire results are presented in the Supplementary Materials (Table S9).

The cheekpiece type of the snaffle bit or bridoon bit within the double bridle predicted component 8 scores, with eggbutt cheekpieces predicting higher component 8 scores than loose ring cheekpieces (Est. = 0.92, CrIs [0.45, 1.39]). There was a trend towards the interaction between time in the <−10° angle group and bridle type being predictive of component 8 (Est. = 0.0215, CrIs [−0.0001, 0.0432]), with a positive correlation between time in the <−10° angle group and component 8 for snaffle bridles and a negative correlation for double bridles.

No other variables in the main dataset models predicted component 8 scores (Table S17).

### 3.4. Noseband Type Models

The predictive and trend results for the noseband BRM models are presented in Table 14.

In the component 4 noseband BRM, noseband type predicted higher component 4 scores with a flash noseband than with a cavesson (Est. = 0.91, CrIs [0.36, 1.48]). The interaction between noseband type and average head angle predicted a positive relationship between component 4 scores and average head angle for flash nosebands compared to a negative relationship for cavesson nosebands (Est. = 0.13, CrIs [0.03, 0.22]). In the component 6 noseband BRM, noseband type showed predictively higher component 6 scores with a cavesson noseband than with a flash noseband (Est. = −0.66, CrIs [−1.29, −0.02]). In the component 7 noseband BRM, noseband type was not predictive of component 7 (Table S18).

### 3.5. Inter-Observer Scoring

There was significant concordance between observer scores for mouth open events, tongue exposure events, tail swishing, spooking, and rearing behaviours (Table 15). The results were not significantly concordant for all other behaviours. However, there was no significant difference between behavioural scoring difference (original score minus secondary score) when compared between snaffle bridles and double bridles (independent *t*-test, df = 162.335, T = −1.169, *p* = 0.244). There was a significant difference between the two observer scores when compared between each conflict behaviour (paired *t*-test, df = 169, T = −2.2287, *p* = 0.023). When comparing the two observer scores between each conflict behaviour within the separate bridle type samples, there was a significant difference between the two observer scores for horses wearing snaffle bridles (paired *t*-test, df = 101, T = −2.075, *p* = 0.041) but was no significant between observer scores for horses in double bridles only (paired *t*-test, df = 67, T = −0.965, *p* = 0.338). Comparing the observer scores for individual behaviours between the two observers showed a significant difference in behaviour scoring for mouth opening event behaviours only, with significantly higher mouth open event scores for the secondary observer than the original observer (paired *t*-test, df = 9, T = −3.736, *p* = 0.005).

## 4. Discussion

The Principal Component Analysis produced nine components weighted for different combinations of conflict behaviours. The weighting structures highlight both the co-occurrence but also the mutual exclusivity of some conflict behaviour combinations (Table 5). This suggests that certain situations and stimuli evoke specific combinations of behaviour but potentially also that if the horse is engaged in one specific type of conflict behaviour, it cannot engage in another. The following sections identify the primary factors associated with each combination of conflict behaviours and also highlight where certain behaviours appear to be mutually exclusive.

### 4.1. Overall Prevalence of Conflict Behaviours

As found in previous studies, mouth opening (performed by 100% of horses as an event [11.27 times a minute] and by 94.8% as a state [21.66% of the test]) and tail swishing (performed by 86.7% of horses [7.2 times a minute]) were the most commonly performed conflict behaviours [7,16,17]. Additionally, head tossing was performed by a high percentage of horses (69.6%) but not performed frequently (0.41 times a minute), and lip smacking was performed by 50.4% of horses (5.26% of the test). The prevalence of conflict behaviour in this study is higher than previously reported [7,8,9,10]. In some studies, conflict behaviour ethograms score the overall severity of the performance of behaviour (e.g., [10]) whereas other ethogram studies require a specific length of time for a behaviour to be performed before it is recorded (e.g., [9]). These approaches reduce the opportunity for appropriate statistical analysis of different variable effects but also tend to underestimate the total performance of the behaviour. The approach taken here of classifying some conflict behaviours as both events and continuous states is considered to provide a more complete quantification of the behaviour in this respect. The high prevalence of conflict behaviours at dressage competitions reported here and in other studies [7,8,9,10] raises potential welfare concerns [7,8,9,10]. It particularly raises the question of why, when this has previously been well-documented by the scientific community and relayed to the governing body of the sport, these high levels of stress-related behaviours are still being observed across all levels of dressage competition.

### 4.2. Component 1—Mouth-Related Conflict Behaviours 1

As documented in the previous literature [16,17,36], mouth opening (as part of component 1) was the most common conflict behaviour being performed by horses during the dressage tests, with 100% of horses performing this behaviour as a behavioural event and 94.8% of horses as a behavioural state.

Statistical analyses of factors affecting component 1 scores highlight a potential impact of rider skill on the performance of mouth behaviours. For both bridle types, lower component 1 scores were predicted at the regional competition than at the typical competition, which, due to the need to qualify for the regional competition, may be associated with more skilled riders. However, the analyses also presented a more complex picture of an inter-relationship between bridle type and rider skill at the different competition levels (predominantly within regional competitions). For example, when wearing a snaffle bridle, component 1 scores were higher at medium than at elementary levels, but, when wearing a double bridle, this was reversed. The component 1 scores for those at elementary level were also lower for snaffle bridle wearers than for those in a double bridle. Elementary level is the first level where horses are allowed to compete in a double bridle, so this result potentially highlights increased mouth behaviours being associated with those who are newer to the use of a double bridle, newer to competing in it, or possibly less skilled in its use. Multiple factors could contribute to this increase in mouth behaviours at elementary level, including incorrect fit of the double bridle [17,19,37] and the overuse or conflicting use of rein aids from the rider [38], especially if either horse or rider are less experienced using a double bridle. These issues are less likely to occur at elementary level for horses wearing a snaffle bridle because both horse and rider will be more experienced and established in the use of this bridle, having competed at lower levels up to this point. This is not the case for those wearing a double bridle, as its use is prohibited before elementary level and thus both horse and rider may be less experienced in using the double bridle in this situation.

Above medium competition level, for both bridle types, there was a general trend for component 1 scores to increase. The demand for higher-level movements at these higher competition levels may contribute to the rider using the reins in a way that increases component 1 behaviours, especially if horse or rider are not achieving these movements correctly [20]. For example, mouth opening was frequently observed during the movement of ‘rein back’, which has previously been attributed to excessive use of the reins to achieve the backwards steps [16,39]. As previously stated, the regional competition had lower component 1 scores compared to typical competitions, and it would be anticipated, therefore, that as the skill of both horse and rider increased with competition level, the amount of mouth-related conflict behaviours would decrease. This was found not to be the case and potentially reflects the greater demand of precise movements at these higher levels resulting in a discrepancy between riding skill and competition demand and, subsequently, an increase in conflict behaviours [7].

Interestingly, riders who regularly received maintenance treatments like physiotherapy also had higher component 1 scores. It is not possible to draw firm conclusions about this result, but it does suggest a potential connection between restriction in the rider’s body, the effect that this may have on the horse, and mouth-related conflict behaviours. The stability of the rider’s body has been found to be particularly affected in the suspension phase of trot [40], especially during sitting trot [41], and this may result in a greater expression of mouth-related conflict behaviours if the body (and hand and rein) are unstable due to rider stiffness or pain. The possibility of interactions between mouth-related behaviours and the influence of the rider’s body was supported by observations made during this study, in that horses who were more prone to high amounts of mouth opening had reductions in this behaviour during lengthened paces (medium and extended trot/canter), when rein tension was reduced. Previous studies have explored the differing rein tensions between bridle types at walk, collected trot, and canter for experienced horse and rider combinations, finding lower overall rein tension for the double bridle than the snaffle bridle [22,42]. However, this research only reflects rein tension for experienced riders, and the effects for less experienced riders have yet to be investigated [22,42]. Exploring how the effects of rein instability change between a greater range movements and how they differ between bridle types, particularly for riders with varying degrees of experience, is an area requiring further study.

Results from the questionnaire’s dataset showed that increased amounts of time spent training in a double bridle increased a horse’s component 1 score. Those who wore a double bridle for more than half of their training rides (riding sessions at home) had higher component 1 scores than those who did not wear a double bridle. Additionally, those who wore a double bridle for more than half of their training rides trended towards higher component 1 scores than those who wore a double bridle for less than half of their training rides. Potential causes of increased mouth behaviours with more time in a double bridle may reflect poorly fitted double bridles, such as, for example, if they are too large for the oral cavity and prevent the horse from closing its mouth fully, which may lead to restricted breathing [19], compression of the tongue [37], and oral lesions [17,19]. The impact of the amount of time a horse spends wearing a double bridle is an area requiring further study.

Finally, the amount of time the horse spent with its head more than 10° behind the vertical (hyperflexed) trended towards a negative relationship with component 1 scores, whilst horses ridden at 0–10° behind the vertical did not. This is an interesting finding given that the previous literature has associated hyperflexion with increased oral conflict behaviours [8,9,18]. These studies were, however, predominantly conducted at higher-level competitions, where more riders were likely to be professional compared to the greater proportion of amateur riders in the present study. In this context, there may be a significant interaction between rider ability and head angle that was not highlighted within the analyses of this study.

### 4.3. Component 2—Full Body Conflict Behaviours 1

Results showed that test scores trended towards a negative relationship with component 2 scores. This was the only component that was associated with test scores, suggesting that whole body behaviours, such as reluctance to move, head tossing, and spooking, were more likely to affect judge scoring than more subtle conflict behaviours [10]. Interestingly, component 2 scores trended towards being lower for horses wearing ear bonnets during competition. Some ear bonnets are known to dampen sound, and thus this may be helping to prevent responses to external stimuli during the dressage test [43].

The interaction between bridle type and competition level was also predictive of scores for component 2, with higher component 2 scores at inter 1 when wearing a snaffle bridle than at elementary, medium, or advanced medium level. However, due to very low samples of snaffle bridles at inter 1 level, the accuracy of the results cannot be confirmed. Further research with snaffle bridles at higher levels will help to confirm if there are true predictive results. If further research reveals that the double bridle is associated with lower component 2 scores at higher levels than the use of a snaffle bridle, it could relate to the double bridle having greater precision to control both spooking and head tossing behaviours [21,44]. It is possible that the snaffle alone may provide more freedom of movement and therefore behavioural expression, leading to an increase in certain behaviours. This is further discussed in component 7.

### 4.4. Component 3—Mouth-Related Conflict Behaviours 2

Component 3 scores were not predicted by any of the main dataset variables. There were some predictive results within the questionnaire dataset; however, the category sizes for these variables were small, and therefore it was not considered possible to draw strong conclusions from these results. These results are presented and discussed in the Supplementary Materials (Table S4).

### 4.5. Component 4—Full Body Conflict Behaviours 2

Component 4 was weighted positively for head tilting, head tossing, and tripping but negatively for tail swishing. Snaffle bridle wearers predicted a positive relationship between component 4 scores and average head angle, whilst double bridle wearers predicted a negative relationship between component 4 scores and average head angle. Component 4 behaviours can potentially arise from balance issues or musculoskeletal discomfort. Anecdotally, they are associated with a horse being ‘hollow’, where the horse’s posture becomes disengaged and the curve of the spine dips below a neutral position, decreasing the distance between the spinal processes across the back and the neck and reducing the horse’s ability to move freely [45]. Whilst it is possible for other factors to be influencing this result, a potential explanation is that the bridle type may affect the mechanics behind a disengaged posture. Typically, when a horse becomes hollow, its head comes up [46], as was observed here for the snaffle bridle, where higher component 4 scores were associated with more positive head angles. The reverse was observed for the double bridle (more negative head angles predicted higher component 4 scores), suggesting that these horses were disengaging by placing the head behind the vertical. Raising the head whilst wearing a double bridle would increase the rotational force and therefore the leverage on the curb bit, tightening the curb chain and increasing the pressure on the chin, the cheekpieces of the bridle, the poll, and also the tongue once the curb chain is fully engaged. Conversely, lowering the head behind the vertical would reduce these elements of pressure whilst still allowing the horse to be disengaged. Theoretically, the action of the double bridle should encourage the horse to lower its head in order to achieve correct posture [21]. If, however, the horse is in a disengaged posture with its head behind the vertical, this is obviously not being achieved [46]. This position with the head behind the vertical may not be obvious to less experienced or skilled riders than with the head in a raised position. Interestingly, for snaffle bridles, there was a negative relationship between component 4 scores and the percentage of the test they spent with a head angle of more than 10° behind the vertical (opposite to what was observed for double bridles). This is an unexpected result, and a possible explanation is that being ridden in hyperflexion when wearing a snaffle may be resulting in muscle development [47] that supports a hyperflexed posture, leading to decreased expression of component 4 behaviours. Further exploration of the effect of bridle types on horses’ posture, especially at hyperflexed head angles, is required.

Those who wore flash nosebands had higher component 4 scores than those wearing cavesson nosebands. Although all double bridles were worn with a cavesson noseband, there was not a predictive effect of bridle type for component 4 scores. This suggests that wearing flash nosebands for snaffle bridles alone was sufficient to cause an increase in head tilting, head tossing, and tripping behaviours. Previous studies have reported that flash nosebands are over-tightened more than cavesson nosebands [48], which could be leading to this result. There was a positive relationship between component 4 scores and average head angle for snaffle bridle wearers, mirrored by a positive relationship between component 4 behaviours and average head angle for flash noseband wearers.

The interaction between noseband type and average head angle for component 4 scores mirrored the previous result for the interaction between bridle type and average head angle, with a positive relationship for flash nosebands compared to a negative relationship for cavesson nosebands. This could suggest that the type of noseband the horse is wearing could potentially contribute to the previously described connection between head placement and component 4 scores. Further research to investigate the effects of equipment items on posture and head position should take the type of noseband into account as a factor to investigate further alongside bridle type.

The interaction between bridle type and competition level was predictive of component 4. Snaffle bridle wearers predicted higher component 4 scores at elementary level than at medium or inter 1 level, whereas component 4 scores were similar across all levels when wearing a double bridle. This suggests that when wearing a snaffle bridle, component 4 behaviours reduce as the skill of the rider increases at higher competition levels. The same relationship does not apply to the double bridle and may reflect the greater degree of skill required for a rider to achieve the same level of balance in a snaffle bridle as a double bridle [49]. Results for component 4 are the opposite to results for component 1, where mouth-related conflict behaviours decreased as the skill of the rider increased when using a double bridle. This highlights a potential discrepancy between the level at which a rider is competing and their degree of skill in using a double bridle in a way that allows them to achieve greater control of the horse without causing an increase in conflict behaviours, i.e., it is possible that riders may be able to improve the horse’s balance (thus reducing component 4 behaviours) whilst using the double bridle in a way that may still increase mouth-related conflict behaviours (component 1).

Interestingly, higher component 4 scores were positively predicted by how much the horse was patted at the end of the test. This may relate to less experienced horses, who therefore show more weakness and consequently more component 4 behaviours, still being rewarded by their rider for their effort.

### 4.6. Component 5—Full Body Conflict Behaviours 3

Component 5 weighted positively for tail swishing and sclera exposure and negatively for spontaneous gait change and lip smacking. Competition level and its interaction with bridle type were predictive for component 5 scores, with advanced medium level generally predicting higher component 5 scores than all other competition levels for both bridle types. This increase in conflict behaviours in the middle range of the competition level is difficult to explain, but it may reflect a sub-group of riders and horses who are using the double bridle for the first time. Interestingly, there was also an interaction between competition type and bridle type where double bridles predicted a higher component 5 score than snaffle bridles at the regional competition only. Higher physical and psychological demand in regional (compared to typical) competition would be anticipated for both horse and rider, which may, in the context of the double bridle, lead to increased tension in the reins and added leverage of the curb bit [44] and thus cause conflicting signals for the horse [38]. Component 5 scores were also higher for horses that were ridden with their head more than 10° behind the vertical for more of their test, again suggesting that this form of riding may be stressful for the horse. It is interesting to note that component 5 was weighted positively for tail swishing and sclera exposure and negatively for spontaneous gait change and lip smacking, suggesting that these sets of conflict behaviours are mutually exclusive. Indeed, where lip smacking and sclera exposure are weighted in other components (e.g., components 2 and 6), these are again exclusive to tail swishing and sclera exposure (components 4 and 8). This may suggest different forms of stressors inducing different forms of stress responses (conflict behaviours) and also that individual horses may respond differently to ridden states of acute stress through the expression of different combinations of conflict behaviours. This individual variation was casually observed during the behavioural observation of this study (e.g., horses more oriented towards certain groups of conflict behaviours, like mouth-related conflict behaviours, versus full body conflict behaviours) and thus may have existed as ‘noise’ in the statistical analyses of the behavioural data. Further research, as outlined in Section 4.13, using more controlled studies will help elucidate these individual responses and identify types of horses more prone to performing certain groups of conflict behaviours.

### 4.7. Component 6—Training-Related Conflict Behaviours

The weighted behaviours of component 6 (positively for incorrect canter lead and spontaneous gait change and negatively for tongue exposure) appear to relate to the training and conditioning of the horse. It is known that poor conditioning can increase the likelihood of mistakes with the canter lead and gait [39]. Whilst canter lead mistakes can also be related to lameness [50], the majority of mistakes observed during this study were unwanted flying changes. There were many predictive results for component 6, making it a complex component, but the most influential appeared to be competition level and rider skill, particularly in the use of the double bridle.

As previously discussed for other factors, higher competition level predicted higher component 6 scores, again suggesting that increased demand on the horse and/or rider led to more ridden mistakes. However, higher component 6 scores were also predicted by the typical (compared to regional) competition, which was the opposite of the result reported for component 5. This may suggest that training-related conflict behaviours are not only the result of mistakes due to increased psychological and physical demand but may simply be down to less experienced riders and/or horses making errors during the dressage test.

Double bridles predicted higher component 6 scores than snaffles, which was contrary to expectation, as the correct use of a double bridle should result in greater balance of the horse (as observed for component 4). It may be possible that despite the double bridle allowing the rider to give more specific aids, it also allows the rider to present more confounding aids if used incorrectly [38], leading to errors, such as incorrect canter lead and spontaneous gait change. Indeed, the interaction between competition type and bridle type showed higher component 6 scores when using a double bridle at the typical competition. This result again highlights how double bridles may lead to reductions in some behaviours (as seen for component 4) but also increase other behaviours (component 1). Comparing results between a competition and a more controlled environment may help to identify which of these differences between behavioural components relate directly to the double bridle and which are more likely related to the rider’s ability. The noseband type results mirrored the results of the bridle type, with higher component 6 scores for cavesson nosebands (the only type worn with a double bridle) than flash nosebands. This suggests that the noseband type could be a contributing factor to the bridle type results for component 6, and further research into the effects of bridle types may find it beneficial to consider the effects of noseband types.

Horses that spent more than half of their training rides wearing a double bridle predicted higher component 6 scores than those who did not wear a double bridle. This association with component 6 is interesting, as it might be expected that increased time wearing a double bridle would increase tongue behaviours due to the additional space they take up in the mouth. Possibly, the double bridle’s greater number of points of contact result in less pressure on the tongue directly, as rein tension across both reins for the double is less than that for the single reins of the snaffle bridle [22,42]. This highlights the need to study the long-term effects of double bridle use, as the horse may adapt to its use over time in a way that might or might not be impacting its welfare.

There was an unexpected predictive result of a negative relationship between component 6 scores and the percentage of the test with the horse’s head angle more than 10° behind the vertical. Those spending large proportions of their test within this posture may be trained in a more hyperflexed position [8,9], leading to conditioning of muscles [47] that results in less training-related mistakes (component 6) despite its negative effects on the horse’s body [8,9,11,18,33,34,35].

Component 6 was the only component for which rider gender was predictive, with higher scores for male riders than female riders. On average, a male rider will be stronger, taller, and heavier [51], as well as having a different centre of gravity, as male and female riders naturally sit differently within the saddle [52], which may all contribute to the movements of the rider’s body being more influential on the horse. Therefore, any loss of balance from the rider that may result in component 6 behaviours will have a greater effect on the horse and these behaviours for a male rider.

### 4.8. Component 7—Spook-Related Conflict Behaviours

Rearing can be caused by confusing and conflicting signals from the rider [53], which may occur whilst trying to control a spook, as well as underlying physiological issues that require veterinary intervention in more extreme cases [54]. Snaffle bridle wearers predicted higher component 7 scores than double bridle wearers. The action of the double bridle allows for more specific control of the horse [21,44] and therefore may be more effective at controlling spooks. If it is used correctly to promote correct posture and self-carriage [55], the horse may be less able to spook when its body is engaged. The double bridle may also deter the horse from lifting its head to either spook or rear due to a higher head angle increasing pressure within the bridle system, as explained in component 4. In addition, horses in a double bridle may have more competition experience and a more established partnership with their rider, whereby the rider is more able to predict and prevent potential spooks. The noseband type results showed no predictive results for component 7, suggesting that the bridle type results are not being influenced or determined by the type of noseband.

Higher component 7 scores were also predicted by wearing a snaffle bridle at the typical competition only. This again suggests that regardless of competition experience, the double bridle is generally linked to reduced spooks and rears. It also suggests that horses with more competition experience will also have acclimated or habituated to the competition environment [56] and therefore be less likely to spook or rear.

### 4.9. Component 8—Full Body Conflict Behaviours 4

Component 8 behaviours were tail swishing, tripping, and head nodding, and they may relate to balance and excessive tension in the body or potentially indicate musculoskeletal issues [57,58].

The type of cheekpiece on the snaffle or bridoon bit was predictive of component 8 scores, with eggbutt cheekpieces higher than loose ring cheekpieces. The eggbutt is more likely to be chosen for horses who require more support in their balance due to the greater stability of the bit, pressure on the horse’s cheek, and its more direct translation of the rein’s pressure to the mouth [59], leading to an association between this bit and higher component 8 behaviours. The loose ring cheekpiece, on the other hand, will absorb a small amount of inconsistency in the rider’s rein due to its more indirect translation of rein aids [59,60], which could reduce confounding signals to the horse [38] as well as balance issues arising from hand instability of the rider alongside instability in the tongue–bridle connection [61], thus reducing component 8 behaviours.

The interaction between bridle type and the percentage of the test when the horse’s head was 10° or more behind the vertical trended towards being predictive of component 8 scores. For those horses wearing a snaffle bridle, increased time spent with the head 10° or more behind the vertical was predictive of higher component 8 scores, whereas for those wearing a double bridle it was the reverse. As was discussed for component 4, extreme head angles in the context of the double bridle may decrease the expression of behaviours like head nodding due to the pressure and leverage action of the curb bit. The opposite result for the snaffle bridle potentially reflects the true effect of hyperflexion on component 8 behaviours, which was masked by behavioural restriction for those wearing double bridles. Hyperflexion is considered to act as both a physical and psychological stressor [8,11,18,33,34,35] and would thus lead to an increase in component 8 behaviours.

### 4.10. Additional Findings from the Questionnaire’s Statistical Analysis

The questionnaire’s analysis produced some results that were directly related to the behavioural effects of the use of different bridle types; however, due to the limited sample size of the questionnaire and, subsequently, the small category sizes, the true direction of these results is not clear. The full results and discussion points are available in the Supplementary Materials (Tables S2–S9). The type of bit the horse wore (i.e., the type of mouthpiece and cheekpieces on both snaffle and double bridles) had predictive results for components 1, 2, 4, 5, 6, and 8, with different bit types being predictive across different components. Additionally, the length of time since a horse first started wearing a double bridle also had trend and predictive results across components 1, 3, 5, and 6. The amount of time since a horse’s last dental examination was predictive for component 1, and its interaction with bridle types had trend and predictive results for components 1, 2, 3, 4, and 8. Whilst the sample sizes for these results do not allow conclusions to be drawn about their effects on the horse’s behaviour, it is heavily suggested that they may be influential on the behaviours in response to the use of different bridle types. As such, these areas of different types of bits, length of time the horse has been wearing a double bridle, and the interaction of different bridle types with the horse’s dentition are good candidate factors for further research to gather more information on their interactions with bridle types.

### 4.11. Inter-Observer Scoring

The following discussion is of the results comparing the original raw behavioural scores of 10 randomly selected horses with the behavioural scoring of a blinded secondary observer. There was no significant difference in ‘scoring difference’ (secondary observer behavioural scores subtracted from original observer behavioural scores) between snaffle and double bridles, suggesting that the original scoring was not biased towards higher scores for one bridle type than the other compared to the behavioural scoring of the blinded secondary observer.

There was a significant difference between the original behavioural scores and the secondary behavioural scores, meaning that the behaviours were scored differently by each observer. When comparing this difference within bridle types, there was a significant difference between observer behavioural scores for snaffle bridles only. However, as the ‘scoring difference’ was not significantly different between bridle types, the cause of the difference in behavioural scoring is unlikely to be due to bias towards a certain bridle type. When investigating further, comparing differences in scoring between bridle types for individual behaviours, only mouth opening behaviours were scored significantly differently between observers, being significantly higher in the secondary observer’s scoring than the original scores. The concordance testing showed significant concordance between the original and secondary behavioural scores for mouth open events, tongue exposure events, tail swishing, spooking, and rearing behaviours. The concordance result means that the ranking of the number of mouth opening events performed by each horse was the same between observers, further suggesting the difference in scoring was not due to a bias in the original scoring but due to a difference in the precision of mouth behaviour scoring. As discussed in the limitations section, the scoring of mouth behaviours may have been more subjective than for other behaviours that are easier to distinguish. The quality of the video footage due to limited zoom, especially when the horse was further away from the camera, may have made it more difficult to distinguish an open mouth from a closed one, resulting in different counts between scorers. Additionally, the secondary observer was not as practiced at scoring and therefore may have been less accurate at recognising pink markings around the mouth or changes in the amount of foam around the mouth. This may have caused additional counting of mouth behaviours when the horse was further away from the camera. This could be mitigated in the future by using higher-quality footage. Concordance testing also shows that more distinct behaviours are more likely to be scored consistently between observers. Behaviours that were rarer, such as sclera exposure, head tossing, or head tilting, were not scored concordantly, and this is likely because the secondary observer had not encountered scoring these behaviours as frequently and as such was not scoring them as consistently. These differences in behavioural scoring highlight some of the potential issues in making comparisons between different conflict behaviour studies where behaviours may be interpreted and scored differently. This also highlights the potential value of video documenting (and making publicly available) all forms of equine conflict behaviour in order to help standardise behavioural studies going forward (discussed further below).

### 4.12. Limitations

The resolution of the camera limited the discernment of some behaviours and may have caused inaccuracy in the measurement of mouth opening, particularly with the presence of foam or pink markings around the mouth. Error was reduced during scoring by noting markings and the extent of any foam before scoring began, as well as monitoring foam throughout the test to note if it changed the look of the mouth. It was possible to do this accurately when the horse was closer to the camera. Some behaviours that occurred at the front of the horse would not have been visible at all angles, such as those occurring at the mouth, so their true frequencies will have been higher. Some observed behaviours were not added to the ethogram due to high subjectivity and lack of measurability, such as tension and arrhythmia. The lack of concordance for some behaviours between primary and secondary observers also suggests subjectivity in behaviour interpretation. As previously discussed, this is potentially problematic when comparing results between studies on equine conflict behaviours. Going forward, there may be a significant advantage in providing an online training facility to help standardise the interpretation and scoring of this class of behaviours. This has been previously done for animal facial expressions, including the horse [62], and it has greatly helped the consistency with which these behaviours are now interpreted and scored.

The questionnaire’s dataset was small (*n* = 28), and therefore some category sizes were very limited for some variables, as previously discussed, and therefore it was not possible to obtain strong results. However, the frequency of predictive results for some of the questionnaire’s variables suggest that they are strong candidates for future research. There was also a limitation of the number of snaffle bridles at PSG and inter 1 levels, as snaffle bridles are less commonly used at these levels, and the number of each bridle type was relatively unbalanced across most levels. In order to assess the impact of different bridle types at higher levels adequately, more research with snaffle bridles and higher-level horses is required, as discussed in the future directions section.

### 4.13. Future Directions

The discussion has identified some key areas for further research. Firstly, comparison between the effects of double and snaffle bridles at higher levels of competition is necessary. Due to the mandatory use of double bridles at levels above FEI CDI2*, it is rare for snaffle bridles to be used at PSG level and above. With public pressure to reconsider the mandatory use of double bridles [6], it is vital to understand how the effects of snaffle and double bridles differ at these higher levels to ascertain if a change to rules surrounding the mandatory use of double bridles is viable and in the best interest of the horses’ welfare or only in the interest of public perception of welfare. The results of the current study suggest that skill (both horse and rider) may be influential with regard to conflict behaviours in connection to bridle type. A comparison of behaviour between bridle types at higher competition levels may provide more context to the differences between bridle types with more skilled horses and riders.

Other considerations are the environment within which the behavioural observations occurred. By using a competition environment, the current study considered the applied use of the bridle type in conjunction with a range of additional factors that provided some level of inference of rider skill. Further research is needed in a more controlled environment (controlling the fit of the bridle, the skill of the rider, the horse’s acclimatisation to the riding environment, etc.) in order to identify more specifically the differences in behaviour that can be attributed solely to bridle type.

Both the long-term effects of wearing a double bridle and the effects of how frequently horses are wearing a double bridle also warrant further investigation, both from a physical and psychological position. How the horse’s dentition interacts differently with each bridle type also appears to influence behaviour and thus warrants further exploration in order to assess the effects of ongoing or unnoticed dental issues. The effects of hand stability on rein tension have previously been explored [40], but understanding how the resulting forces the horse experiences from rein instability in different bridle types, as well as how different movements may affect how the rider’s rein tension changes and the subsequent impact this has on the horse’s behaviour, also merit further investigation.

The horse’s posture in relation to its head appeared to differ between bridle types (see component 4). Further exploration of postural differences with different bridle types is warranted. Additionally, comparing the rider’s perception of a horse’s level of engagement to its actual posture would help provide additional information about rider skill, disengaged postures, and head angle when ridden.

## 5. Conclusions

A high prevalence of conflict behaviours was recorded during this study, particularly for mouth opening (performed by 100% of horses as an event [11.27 times a minute] and by 94.8% as a state [for 21.66% of the test]) and tail swishing (performed by 86.7% of horses [7.2 times a minute]). This study not only demonstrates that double and snaffle bridles affect equine behaviour differently but highlights the complexity of additional influencing factors. An overall theme of the study was different behavioural effects of bridle type within and between components. For example, the level of component 4 scores (head tilting, head tossing, and tripping, with low levels of tail swishing) was similar across competition levels for the double bridle but increased with competition level for the snaffle bridle. Conversely, component 6 scores (incorrect canter lead, gait change, and negative tongue exposure) were higher when using a double bridle compared to a snaffle bridle, especially at the typical competition compared to the regional. Similarly, component 1 scores (mouth open, tongue exposure, jaw twisting) were higher at lower competition levels for double bridles only but increased at higher competition levels for both the snaffle bridle and the double bridle. These differing effects of bridle type at different competition levels for the different groups of conflict behaviours suggest that the effect of bridle type on conflict behaviours is complex. A similarly complex pattern was observed for the interaction between bridle type and the head angle with which the horse was ridden. Component 4 scores (head tilting, head tossing, tail swishing, and tripping) were predicted to be higher for snaffle bridles when the horse was ridden with a higher average head angle but also when it was ridden with a lower average head angle with the double bridle. Equally, component 8 scores (tail swish, tripping, and head nodding) were predicted to be lower with a double bridle compared to a snaffle bridle but only when the horse was again ridden with a head angle of more than 10° behind the vertical for a greater percentage of its test. These results demonstrate a multifaceted interaction between bridle type, inferred rider skill level, and riding style. These results also demonstrate that in many instances, particularly in the context of bridle type and ridden head angle, certain groups of conflict behaviours are not expressed. This may be due to (1) different forms of stressors eliciting different behavioural responses, (2) some of the conflict behaviours not actually representing momentary or ongoing states of acute stress, i.e., they are not conflict behaviours, or (3) certain combinations of bridle types and ridden head angles preventing the horse from expressing some conflict behaviours but not others. Further hypothesis-driven research to assess the impacts of individual and combined forms of affecting factors on conflict behaviour, as identified in this paper, is needed in order to fully understand the complex interaction of bridle type, rider skill, and ridden head angle and its impact on the welfare of the ridden horse.

## Figures and Tables

**Table 1:** The number of horses ridden in each bridle type (double or snaffle) across each competition level.

Competition Level	Number of Double Bridles	Number of Snaffle Bridles	Percentage of Double Bridles	Percentage of Snaffle Bridles
Elementary	12	41	22.6%	77.4%
Medium	11	16	40.7%	59.3%
Advanced Medium	4	5	44.4%	55.6%
Prix St. George (PSG)	25	2	92.6%	7.4%
Intermediate I	18	1	94.7%	5.3%
All Levels	70	65	51.9%	48.1%

**Table 2:** Ethogram of recorded event and state behaviours.

Behaviour	Description
**Event Behaviours**	
Head tilt	Tilt of the head where the poll or nose was not in line with the centre of the horse’s body, e.g., when the body leant around a corner, this was not a head tilt, as the head was in line with the body’s centre. Each instance in which the horse tilted its head out of alignment with the centre of its body was counted as a single event, regardless of the length of time during which it was tilted.
Head tossed/lifted out of frame	A loss of frame, either through the lifting or tossing of the head. This included head lifting in transitions and coming ‘above the bit’. Any lift or toss of the head out of frame was counted as an event, regardless of the length of time the head was out of frame.
Ears pinned	Ears rotated and flattened backwards; does not include rotation only. Each instance of ear rotation was counted as an event regardless of the length of time during which the ears were flattened.
Sclera exposed	Exposure of the whites of the eyes. Each instance of the white of the eye becoming visible was counted as a single event, regardless of the length of time during which the sclera was visible. It must be noted that for some horses, the sclera is always visible, and therefore it is not possible to accurately determine when the eyes change in such a way that would expose the sclera on a horse with a differently proportioned eye. However, this was not the case for any horses in the current study, so this does not impact the recording of this behaviour in this instance.
Mouth opening	Single opening events of the mouth, including a single opening of the lips alone and all opening with separation of the teeth. Each instance of the mouth opening was recorded as a single event, regardless of the length of time during which the mouth was open. When the mouth was continuously opening and closing, each instance of separation of the teeth was counted as an individual event, and the length of time during which the continuous movement of the mouth occurred was recorded as a state. The opening of the lips was counted as a mouth opening event in the case of a single opening of the lips occurring at one time. If there were two seconds or more between individual performances of opening of the lips only, this was counted as individual mouth opening events. If the lips alone were moving continuously (less than two seconds in between each performance of lip movement) for more than a single opening of the lips without any separation of the teeth, this was classed as a lip smacking state. Refer to the video material provided for a visual representation of mouth opening and lip smacking to further clarify this distinction.
Tongue exposed	Exposure of the tongue where the tongue left the boundaries of the mouth, including protruding forward or sideways or bulging beyond the mouth. This did not include visualisation of the tongue when the mouth was open, or the jaw was twisted if the tongue remained within the boundaries of the mouth. Each instance of visualisation of the tongue was counted as an event behaviour regardless of the time during which the tongue was visible. The length of time was recorded as a state behaviour.
Jaw twisting	Twisting of the jaw, where the lower jaw was no longer in line with the upper jaw. Each instance of jaw twisting was recorded as an event regardless of the length of time during which the jaw was in a twisted position; the length was recorded as a state. In the event that the jaw was twisted to one side and then twisted across to the other side, an event was recorded for each crossing out of line of the upper jaw (i.e., if the jaw twisted to the right and then immediately twisted to the left, this was counted as two events of jaw twisting). Refer to the video material provided for a visual representation of jaw twisting.
Tail swish	Movement of the tail outside of its natural movement with the horse. Tail swishing was identified from the movement of the dock, not the movement of the tail hairs themselves, to prevent the natural movement of the tail being confused with a tail swish, and it included both side to side, up and down, and circular tail movements. Each change in direction of the dock was counted as a tail swish event if the tail was swishing side to side repeatedly. If the tail was swishing continuously, each individual movement of the tail away from its position when neutral was counted as an event. Refer to the video material provided for a visual representation of counting tail swishing events.
Incorrect canter lead	Spontaneous change of canter lead or incorrect canter lead when making a canter transition or when canter became disunited. Corrections of the rider to the correct canter were not included. Each canter lead mistake outside of the rider’s corrections was counted as an individual event, regardless of the length of time during which the lead change or incorrect/disunited canter was performed.
Change of gait	Spontaneous change between gaits, either up or down, or failure to perform a necessary change of gait. During a walk to canter or canter to walk transition, there was an allowance of two steps of trot before an incorrect gait event was counted. Corrections back to the correct gait were not included. Each instance of an incorrect gait, regardless of the length of time for which the incorrect gait was performed (with the exception of the aforementioned walk to canter transition), was counted as an individual event.
Stumble/trip	Any trip or stumble. Each instance in which a stumble or trip occurred was counted as an individual event regardless of the length of time during which the stumble or trip took place.
Spook	Any spook or sudden change of direction against the rider’s direction. Backing up as part of the spook was not counted as reluctance to move. Once the horse had become stationary or had begun moving forward again following the occurrence of a spook, reluctance to move was counted if the horse stopped and began to back up again. Each instance of a spook was counted as an individual event. In the instance that a horse was to spook twice in a row and it was counted as two events, the spooks were differentiated by the horse either becoming fully stationary for more than a full second or moving forward in the required direction for the test as directed by the rider for at least four steps between the two spooks. If this stationary/forward movement was not performed between the times of spooking, it remained one continuous spook and was counted as a single event.
Reluctance to move	Reluctance to move forward, spontaneous stop, backing up, or napping. This did not include the horse stopping to defecate or backing up during the performance of a spook behaviour. Each instance of a horse performing this behaviour was counted as a single event. For a reluctance to move event to be recorded following a spook, the horse would have had to either become stationary for more than a full second or move forward in the required direction for the test as directed by the rider for at least four steps after the spooking event and then either stop, become reluctant to move, back up, or nap. Two reluctance to move events had to be separated by the same stationary or forward movement period to be counted as sperate events.
Rearing	Front legs simultaneously left the ground. The context of the horse’s movement was considered; for example, the moment of front leg suspension in a canter pirouette when both front legs leave the floor was not counted as a rear because it was an intentional part of the movement. Any unintended simultaneous lifting of both front legs off the ground that was not part of a test movement was counted as a single rearing event. The height of the rear did not change the outcome of how it was counted as an event.
Bucking/kicking	Both hind legs left the ground simultaneously or a single hind leg struck out. Each simultaneous lift of the hind legs or singular leg strike out was counted as an event. Any simultaneous movement of the hind limbs that was clearly part of the test movement (for example, if during a canter pirouette the horse loses rhythm of its hind legs and performed a ‘jump’ behind with their two hind legs simultaneously) was not counted as an event.
**State Behaviours**	
Lip smacking	Lips were continually moving, either smacking together, a single upper or lower lip was moving, the lips appeared to reach out, or the lips were quivering. A continuous lip movement was counted as lip smacking if it occurred for more than one second and if there were less than two seconds between each movement of the lips. If there were more than two seconds between each opening and closing movement of the lips and the movement lasted for less than one second, this was classed as a mouth opening event. Refer to the video material provided for a visual representation of mouth opening and lip smacking to further clarify this distinction and to see the variations in lip movement classed under lip smacking.
Mouth opening	The mouth was in a state of continually being open or continuously opening and closing, including separation of the teeth. A state behaviour was recorded for the length of time the mouth was either continually open or opening and closing if it occurred for more than one second. For opening and closing to be continuous, there must have been less than two seconds between when the mouth closed and then reopened.
Head nodding	Head nodding became exaggerated compared to typical way of going or the head nod was no longer in rhythm with the pace. The length of time for which exaggerated or out of rhythm head nodding was performed was recorded as a state behaviour.
Tongue exposed	Exposure of the tongue where the tongue left the boundaries of the mouth, including protruding forward, sideways, or bulging beyond the mouth. This did not include visualisation of the tongue when the mouth was open, or the jaw was twisted if the tongue remained within the boundaries of the mouth. This was recorded as a state behaviour when the tongue left the boundaries of the mouth continuously for more than one second or when the tongue was moving in and out of the mouth continually for more than one second. If there were two seconds or more between individual instances of tongue exposure, the gap between these events did not count towards the length of a tongue state behaviour.
Jaw twisting	Twisting of the jaw, where the lower jaw was no longer in line with the upper jaw. The length of time during which the jaw was held in a twisted position was recorded. Each instance of jaw twisting that lasted longer than one second was counted as a state behaviour and an event behaviour. In the instance that the jaw twist lasted less than one second, it was only counted as an event behaviour. If the jaw repeatedly moved from a twisted to an untwisted position with less than two seconds between each twist, this was counted as a state from the first jaw twist to the end of the last, as well as each individual twist being counted as an event. If the jaw was twisted and then immediately changed to being twisted to the other side, the count of the length of time of the state behaviour would begin when the jaw initially became twisted, continue through the change inside of the twist, and then end when the jaw returned to a neutral position after being twisted to the other side. Please refer to the video material provided for a visual representation of the jaw twisting state.
Reluctance to move	Reluctance to move forward, spontaneous stop, backing up, or napping. If the behaviour lasted less than one second, it was only counted as an event behaviour; if it lasted one second or more, it was counted as both an event and a state behaviour, and the length of time until the horse moved forward towards the designated test direction was counted. This did not include the horse stopping to defecate or backing up during the performance of a spook behaviour. The horse had to either become stationary for more than a full second or move forward in the required direction for the test as directed by the rider for at least four steps after a spooking event and then either stop, become reluctant to move, back up, or nap for it to count as reluctance to move following a spook.

**Table 3:** Variable titles, initials, and descriptions for variables of the whole dataset and for those in the questionnaire that are discussed. Additional questionnaire variables and their results are presented within the Supplementary Materials (Table S1).

Variable	Categories	Initial	Description
**Whole dataset categorical data**			
Bridle Type	Snaffle bridle	S	A bridle with only one bit.
	Double bridle	D	A bridle with two bits, a bridoon (snaffle), and a curb bit (often called a Weymouth), which is a leverage bit. The angle of the curb bit is positioned using a curb chain.
Competition Level	Elementary	E	Elementary level—walk, trot, and canter, lengthened strides, lateral movements, including leg yield, and collected trot and canter.
	Medium	M	Medium level—as previous, with increased difficulty of lateral movements, extended paces, walk pirouette, and halfpass in trot/canter.
	Advanced medium	A	Advanced Medium leve l—as previous, with flying changes.
	Prix St. George (PSG)	P	Prix St. George level—small tour—as previous, with tempi changes (multiple flying changes), canter pirouettes, and increased difficulty of movements.
	Inter I	I1	Intermediate I level—small tour—as previous, with increased complexity of movements.
Competition Type	Regional	R	A British Dressage regional competition where riders must qualify to compete. Qualification requires gaining enough points by performing tests at ‘typical’ competitions (more points are gained by achieving higher scores) and achieving a minimum score. This competition was held outdoors.
	Typical	T	A regular day to day British Dressage competition in which any affiliated rider may enter. This was held in an indoor arena.
Rider’s Gender	Male	Mr	Riders were male.
	Female	Fr	Riders were female.
Use of Spurs	Spurs	S	The rider wore spurs.
	No spurs	nS	The rider did not wear spurs.
Use of Ear Bonnet	Ear bonnet	EB	The horse wore an ear bonnet.
	No ear bonnet	nEB	The horse did not wear an ear bonnet.
Snaffle/ Bridoon Cheekpiece Type	Loose ring	Lr	The round cheekpiece of the bit freely rotated.
	Eggbutt	Eb	The rounded rings of the cheekpiece were fixed. Provides some pressure on the cheek when turning.
	Hanging cheek	Hc	A lower case ‘b’-shaped cheekpiece, which was fixed. The arm attached to the cheekpiece reduces poll pressure at lower rein tensions and increases poll pressure at higher rein tensions.
	D-ring	Dr	The cheekpieces were fixed and D-shaped. Provides more cheek pressure during turning than an eggbutt.
Noseband Type	Cavesson	Cn	A plain noseband that fastened around the upper portion of the nose. It was not possible to distinguish between regular cavessons and crank (Swedish) nosebands, so both were classed under cavesson.
	Flash	Fn	Worn with a cavesson noseband and has an additional lower strap that fastened below the bit under the chin.
	Drop	Dn	A plain noseband that fastened around the lower portion of the nose and fastened below the bit.
	Four-ring-drop	Rn	A drop noseband that had an additional strap positioned under the mandible at the position where the underneath portion of a cavesson noseband would sit. As such, it has a similar effect to the horse wearing both a drop and a cavesson noseband together. The four ‘rings’ allow for some change in angle of the straps, and therefore this may not be fastened as low on the nose as a traditional drop noseband.
**Whole dataset continuous data**			
Patting Frequency			The number of times the horse was patted between the rider saluting at the end of their test and exiting the arena. Each contact of the hand is counted as a single pat. If patted with 2 hands simultaneously, a single pat is counted for each simultaneous contact of the hands with the horse.
Average Head Angle			The average angle of the horse’s head throughout the test, where 0° is vertical and perpendicular to the ground. The average was calculated from measurements taken at 10 s intervals throughout the test.
Test Score			The score awarded to the test by the competition judges. This was scored by a single judge positioned at ‘C’ at the typical competition and an average score across 3 judges positioned at ‘C’, ‘H’, and ‘B’ at the regional competition.
Angle Categories	0° to −10°		The percentage of the test the horse spent with its head in the angle range of 0° to −10°.
	<−10°		The percentage of the test the horse spent with its head in the angle range of less than −10°.
**Questionnaire dataset categorical variables**			
Rider Maintenance Treatments	Regular maintenance treatments	MT	The rider regularly received maintenance treatments, such as physiotherapy or chiropractic treatments.
	No maintenance treatments	nMT	The rider did not regularly receive maintenance treatments.
Time Spent Training in a Double Bridle	Does not wear double bridle	nDB	The horse did not wear a double bridle.
	Less than half of training rides	LH	The horse wore a double bridle for less than half of training rides at home.
	More than half of training rides	MH	The horse wore a double bridle for more than half of training rides at home.

**Table 4:** Raw behavioural data descriptions, including the percentage of horses (*n* = 135) who performed each conflict behaviour during their dressage test, which ranged from elementary level to intermediate I level, as well as the average performance of the behaviour and the standard error of the mean and range that correspond to that average.

Behaviour	Percentage of Horses Performing Behaviour	Average Performance of the Behaviour	Standard Error of the Mean for the Average	Range of Performance
**Event Behaviours**		**Average behaviours per minute**		
Head tilt	39.3	0.21	0.05	0.00–5.09
Head tossed/lifted out of frame	69.6	0.41	0.04	0.00–3.98
Ears pinned	0.0	0.00	0.00	0.00–0.00
Sclera exposed	29.6	0.24	0.06	0.00–6.14
Mouth opening	100.0	11.27	0.93	0.18–67.04
Tongue exposed	25.9	0.25	0.13	0.00–17.43
Jaw twisting	19.3	0.09	0.02	0.00–1.78
Tail swish	86.7	7.15	0.88	0.00–52.72
Incorrect canter lead	5.2	0.01	0.00	0.00–0.34
Change of gait	26.7	0.07	0.01	0.00–1.14
Stumble/trip	8.1	0.02	0.01	0.00–0.62
Spook	8.1	0.02	0.01	0.00–0.71
Reluctance to move	1.5	0.00	0.00	0.00–0.35
Rearing	2.2	0.00	0.00	0.00–0.23
Bucking/kicking	0.7	0.00	0.00	0.00–0.20
**State Behaviours**		**Average percentage of test during which behaviour is performed**		
Lip smacking	50.4	5.26	0.98	0.00–60.50
Mouth open	94.8	21.66	1.93	0.00–97.47
Head nodding	2.2	0.03	0.02	0.00–1.63
Tongue exposed	5.9	0.34	0.18	0.00–18.71
Jaw twisting	6.7	0.10	0.05	0.00–5.82
Reluctance to move	0.74	0.02	0.02	0.00–2.94

**Table 5:** Principal component analysis behaviour loadings calculated using the raw conflict behaviour data (*n* = 135). Eigen values > ±0.3 are shown in bold and denote ‘weighted’ component loadings.

Behaviours	Component 1	Component 2	Component 3	Component 4	Component 5	Component 6	Component 7	Component 8	Component 9
**Event Behaviours**									
Head tilting	0.06	0.05	−0.04	**0.73**	0.19	−0.11	0.13	−0.05	0.02
Head tossed	−0.06	** 0.32 **	−0.07	** 0.54 **	−0.08	−0.03	0.09	−0.12	0.13
Sclera exposed	0.06	0.05	−0.04	0.03	** 0.79 **	−0.03	−0.06	−0.14	−0.11
Mouth open	** 0.86 **	0.01	−0.06	−0.02	0.17	0.01	0.06	−0.07	−0.05
Tongue exposed	** 0.50 **	0.07	−0.05	0.04	−0.30	** −0.39 **	−0.21	−0.30	−0.04
Jaw twisting	** 0.32 **	−0.05	** 0.67 **	−0.10	−0.02	0.02	0.02	−0.01	−0.04
Tail swish	0.14	0.27	−0.12	** −0.35 **	** 0.34 **	−0.04	−0.13	** 0.46 **	0.13
Incorrect canter lead	0.09	−0.04	−0.05	−0.15	0.16	** 0.59 **	−0.03	−0.10	0.01
Gait change	0.03	0.16	−0.10	0.08	** −0.32 **	** 0.74 **	−0.04	−0.04	−0.08
Trip	−0.06	−0.13	−0.06	** 0.59 **	−0.13	0.05	−0.24	** 0.30 **	−0.17
Spook	0.06	** 0.75 **	0.00	0.17	0.06	0.08	** 0.43 **	−0.03	−0.02
Reluctance to move	−0.03	** 0.88 **	0.01	0.03	0.04	0.05	−0.12	0.06	0.01
Rearing	0.01	0.06	−0.05	0.05	−0.08	−0.05	** 0.92 **	−0.01	−0.03
Buck/kick	−0.03	−0.01	−0.04	0.00	−0.08	−0.04	−0.03	−0.03	** 0.93 **
**State Behaviours**									
Lip smack	−0.18	** 0.40 **	−0.16	−0.17	** −0.35 **	−0.25	0.00	−0.24	−0.26
Mouth open	** 0.81 **	−0.06	0.13	−0.02	0.00	0.21	0.01	0.19	0.05
Head nodding	0.00	−0.03	−0.04	0.04	−0.16	−0.11	0.02	** 0.79 **	−0.05
Tongue exposed	−0.10	−0.02	** 0.68 **	0.00	0.04	−0.09	−0.04	−0.06	0.00
Jaw twisting	−0.04	0.02	** 0.83 **	−0.03	−0.05	−0.02	−0.02	0.01	0.01

**Table 6:** Principal component analysis behavioural groupings showing weighted behaviours (eigen value > ±0.3) for each component.

Component	Component Name	Weighted Behaviours for Component	Percentage of Total Variance Explained by Component
Component 1	Mouth-related conflict behaviours 1	Mouth Open Event (+ve)	11%
		Tongue Exposure Event (+ve)	
		Jaw Twisting Event (+ve)	
		Mouth Open State (+ve)	
Component 2	Full body conflict behaviours 1	Head Tossing Event (+ve)	10%
		Spook Event (+ve)	
		Reluctance to Move Event (+ve)	
		Lip Smacking State (+ve)	
Component 3	Mouth-related conflict behaviours 2	Jaw Twisting Event (+ve)	8%
		Tongue Exposure State (+ve)	
		Jaw Twisting State (+ve)	
Component 4	Full body conflict behaviours 2	Head Tilting Event (+ve)	7%
		Head Tossed Event (+ve)	
		Tail Swish Event (−ve)	
		Trip Event (+ve)	
Component 5	Full body conflict behaviours 3	Sclera Exposure Event (+ve)	6%
		Tail Swish Event (+ve)	
		Gait Change Event (−ve)	
		Lip Smacking State (−ve)	
Component 6	Training-related conflict behaviours	Tongue Exposure Event (−ve)	6%
		Incorrect Canter Lead Event (+ve)	
		Gait Change Event (+ve)	
Component 7	Spook-related conflict behaviours	Spook Event (+ve)	6%
		Rearing Event (+ve)	
Component 8	Full body conflict behaviours 4	Tail Swish Event (+ve)	5%
		Trip Event (+ve)	
		Head Nodding State (+ve)	
Component 9	Kicking	Kick Event (+ve)	5%

**Table 7:** Component 1 (mouth-related conflict behaviours 1) predictive and trend Bayesian regression model results. Predictive results are shown in bold to differentiate from trend results. (D = double bridle, S = snaffle bridle; E = elementary, M = medium; A = advanced medium; P = Prix St. George; I = intermediate I; T = typical competition, R = regional competition; MT = rider receives maintenance treatments, nMT = rider does not receive maintenance treatments; nDB = no double bridle, LH = less than half of training rides, MH = more than half of training rides; * denotes category interaction).

Model	Variable	Sample Size	Category Size	Hypothesis	Estimate	Lower CrI	Upper CrI
Main dataset model	Competition level	118	E = 50 M = 24 A = 8 P = 21 I = 15	E > I1	−0.63	−1.34	0.08
		118		M > A	−1.19	−2.48	0.07
		118		**I > M**	**1.31**	**0.47**	**2.15**
		118		**M > P**	**−1.02**	**−1.83**	**−0.23**
	Competition type	118	T = 11 R = 107	**R > T**	**−1.57**	**−3.03**	**−0.08**
	Bridle type * competition level	118	D*E = 11 S*E = 39 D*M = 9 S*M = 15 D*A = 4 S*A = 4 D*P = 20 S*P = 1 D*I = 14 S*I = 1	S > D*E > M	**−1.16**	**−2.18**	**−0.15**
		118		S > D*P > A	2.51	−0.18	5.20
		118		S > D*E > P	**−2.47**	**−4.44**	**−0.50**
		118		S > D*I1 > M	−1.75	−3.80	0.31
		118		S > D*I1 > P	**−3.06**	**−5.87**	**−0.21**
Questionnaire rider maintenance treatment	Rider maintenance treatment	28	MT = 20 nMT = 8	MT > nMT	0.67	−0.04	1.38
Questionnaire time training in double bridle	Training in double bridle	28	nDB = 17 LH = 5 MH = 6	**MH > nDB**	**1.21**	**0.42**	**2.00**
		28		LH > MH	−0.73	−1.55	0.09
Behind the vertical angle categories	<−10 degrees	135			−0.0117	−0.0255	0.0022

**Table 8:** Component 2 (full body conflict behaviours 1) predictive and trend Bayesian regression model results. Predictive results are shown in bold to differentiate from trend results. (D = double bridle, S = snaffle bridle; E = elementary, M = medium; A = advanced medium; P = Prix St. George; I = intermediate I; EB = ear bonnet, nEB = no ear bonnet; * denotes category interaction).

Model	Variable Name	Sample Size	Category Size	Hypothesis	Estimate	Lower CrI	Upper CrI
Main dataset model	Bridle Type * Competition Level	118	D*E = 11 S*E = 39 D*M = 9 S*M = 15 D*A = 4 S*A = 4 D*P = 20 S*P = 1 D*I = 14 S*I = 1	S > D*E > I1	**−0.90**	**−1.80**	**−0.01**
		118		S > D*I1 > M	**1.16**	**0.24**	**2.09**
		118		S > D*I1 > A	1.17	−0.03	2.36
	Ear Bonnet	118	EB = 55 nEB = 63	EB > nEB	−0.16	−0.34	0.02
	Test Score	118			−0.03	−0.07	0.00

**Table 9:** Component 4 (full body conflict behaviours 2) predictive and trend Bayesian regression model results. Predictive results are shown in bold to differentiate from trend results. (D = double bridle, S = snaffle bridle; E = elementary, M = medium; A = advanced medium; P = Prix St. George; I = intermediate I; * denotes category interaction).

Model	Variable Name	Sample Size	Category Size	Hypothesis	Estimate	Lower CrI	Upper CrI
Main dataset model	Bridle type * average head angle	118	S = 60 D = 58	**S > D**	**0.14**	**0.05**	**0.24**
	Patting score	118			0.01	−0.00	0.03
	Bridle type * competition level	118	D*E = 11 S*E = 39 D*M = 9 S*M = 15 D*A = 4 S*A = 4 D*P = 20 S*P = 1 D*I = 14 S*I = 1	**E > I1**	**2.05**	**0.22**	**3.87**
		118		**E > M**	**0.91**	**0.00**	**1.82**
Behind the vertical angle categories	Bridle type * <−10 degrees	121	S = 60 D = 58	**S > D**	**−0.0279**	**−0.0502**	**−0.0057**

**Table 10:** Component 5 (full body conflict behaviours 5) predictive and trend Bayesian regression model results. Predictive results are shown in bold to differentiate from trend results. (D = double bridle, S = snaffle bridle; E = elementary, M = medium; A = advanced medium; P = Prix St. George; I = intermediate I; T = typical competition, R = regional competition; * denotes category interaction).

Model	Variable Name	Sample Size	Category Size	Hypothesis	Estimate	Lower CrI	Upper CrI
Main dataset model	Competition level	118	E = 50 M = 24 A = 8 P = 21 I = 15	**E > A**	**−1.64**	**−2.83**	**−0.45**
		118		**I1 > A**	**−1.39**	**−2.53**	**−0.28**
		118		**M > A**	**−1.72**	**−2.95**	**−0.53**
		118		**P > A**	**−1.22**	**−2.31**	**−0.13**
	Bridle type * competition level	118	D*E = 11 S*E = 39 D*M = 9 S*M = 15 D*A = 4 S*A = 4 D*P = 20 S*P = 1 D*I = 14 S*I = 1	S > D*E > A	1.36	−0.17	2.89
		118		S > D*M > A	1.51	−0.10	3.10
		118		S > D*P > A	2.75	−0.20	5.27
		118		S > D*R > T	−1.32	−2.87	0.23
	Bridle type * competition type	118	S*T = 8 D*T = 3 S*R = 52 D*R = 55	S > D*R > T	**−1.32**	**−2.87**	**0.23**
Behind the vertical angle categories	<−10 degrees	135			0.0106	−0.0025	0.0238

**Table 11:** Component 6 (training related conflict behaviours) predictive and trend Bayesian regression model results. Predictive results are shown in bold to differentiate from trend results. (D = double bridle, S = snaffle bridle; T = typical competition, R = regional competition; Fr = female rider, Mr = male rider; E = elementary, M = medium; A = advanced medium; P = Prix St. George; I = intermediate I; Lr = loose ring cheekpiece, Eb = eggbutt cheekpiece, Dr = D-ring cheekpiece, Hc = hanging cheek cheekpiece; nDB = no double bridle, LH = less than half of training rides, MH = more than half of training rides; * denotes category interaction).

Model	Variable Name	Sample Size	Category Size	Hypothesis	Estimate	Lower CrI	Upper CrI
Main dataset model	Bridle type	118	S = 60 D = 58	**S > D**	**−2.52**	**−4.64**	**−0.38**
	Competition type	118	T = 11 R = 107	**R > T**	**−2.86**	**−4.29**	**−1.43**
	Rider gender	118	Fr= 97 Mr = 21	Mr > Fr	0.47	−0.08	1.01
	Competition level	118	E = 50 M = 24 A = 8 P = 21 I = 15	**I1 > M**	**1.00**	**0.19**	**1.79**
		118		**I1 > P**	**0.73**	**0.16**	**1.29**
	Bridle type * competition type	118	S*T = 8 D*T = 3 S*R = 52 D*R = 55	S > D*R > T	**3.07**	**1.43**	**4.68**
	Snaffle cheekpiece	118	Lr = 98 Eb = 18 Dr = 1 Hc = 1	Hc > Dr	2.43	−0.40	5.23
	Bridle type * competition level	118	D*E = 11 S*E = 39 D*M = 9 S*M = 15 D*A = 4 S*A = 4 D*P = 20 S*P = 1 D*I = 14 S*I = 1	S > D*I1 > A	−2.25	−4.81	0.31
		118		S > D*I1 > P	−1.93	−3.92	0.06
		118		S > D*I1 > M	**−1.93**	**−3.92**	**0.06**
Questionnaire time training in double bridle	Training in double bridle	28	nDB = 17 LH = 5 MH = 6	**MH > nDB**	**1.29**	**0.15**	**2.41**
Behind the vertical angle categories	<−10 degrees	135			−0.0114	−0.0255	0.0027

**Table 12:** Component 7 (spook-related conflict behaviours) predictive and trend Bayesian regression model results. Predictive results are shown in bold to differentiate them from trend results. (S = snaffle bridle, D = double bridle; T = typical competition, R = regional competition; * denotes category interaction).

Model	Variable Name	Sample Size	Category Size	Hypothesis	Estimate	Lower CrI	Upper CrI
Main dataset model	Bridle type * competition type	118	S*T = 8 D*T = 3 S*R = 52 D*R = 55	S > D*R > T	−1.95	−3.60	0.30
	Bridle type	118	D = 58 S = 60	S > D	2.19	−0.01	4.37

**Table 13:** Component 8 (full body conflict behaviours 4) predictive and trend Bayesian regression model results. Predictive results are shown in bold to differentiate them from trend results. (Lr = loose ring cheekpiece, Eb = eggbutt cheekpiece, Dr = D-ring cheekpiece, Hc = hanging cheek cheekpiece; D = double bridle, S = snaffle bridle).

Model	Variable Name	Sample Size	Category Size	Hypothesis	Estimate	Lower CrI	Upper CrI
Main dataset model	Snaffle cheekpiece	118	Lr = 98 Eb = 18 Dr = 1 Hc = 1	**Eb > Lr**	**0.92**	**0.45**	**1.39**
Behind the vertical angle categories	Bridle type * <−10 degrees	135	D = 58 S = 60	S > D	0.0215	−0.0001	0.0432

**Table 14:** Predictive results for noseband Bayesian regression models for noseband type for components 6 (training-related behaviours) and 4 (full body conflict behaviours 2). Predictive results are shown in bold to differentiate them from trend results. (Cn = cavesson noseband, Dn = drop noseband, Fn = flash noseband, Rn = four-ring-drop noseband).

Model	Variable Name	Sample Size	Category Size	Hypothesis	Estimate	Lower CrI	Upper CrI
Component 6 Noseband Model	Noseband type	114	Cn = 62 Dn = 3 Fn = 44 Rn = 5	**Fn > Cn**	**−0.66**	**−1.29**	**−0.02**
Component 4 Noseband Model	Noseband type	114	Cn = 62 Dn = 3 Fn = 44 Rn = 5	**Fn > Cn**	**0.91**	**0.36**	**1.48**
	Noseband type * average head angle	114	Cn = 62 Dn = 3 Fn = 44 Rn = 5	**Fn > Cn**	**0.13**	**0.03**	**0.22**

**Table 15:** Kendall’s W test results for inter-observer scoring. Bold shows significant concordance between the behavioural scoring of the original observer and the blinded secondary observer.

Behaviour	N	W	*p*
**Event Behaviours**			
Head Tilt	2	0.500	0.437
Head Toss	2	0.682	0.198
Sclera	2	0.444	0.534
Mouth Open	2	0.970	**0.042**
Tongue	2	1.000	**0.035**
Jaw Twist	2	0.874	0.073
Tail Swish	2	0.976	**0.041**
Canter Lead	2	0.500	0.437
Gait Change	2	0.875	0.072
Spook	2	1.000	**0.035**
Rear	2	1.000	**0.035**
**State Behaviours**			
Lip Smacking	2	0.918	0.057
Mouth Open	2	0.901	0.062
Nodding	2	0.500	0.437
Tongue	2	0.500	0.437
Jaw Twist	2	0.500	0.437

## Data Availability

Supplementary Information accompanies this paper. Datasets analysed and presented in the current study are available at https://doi.org/10.17605/OSF.IO/Y2DCH.

## Supplementary Materials

The following supporting information can be downloaded at https://www.mdpi.com/article/10.3390/ani15121782/s1, 1. Model Diagnostics, 2. Variables, Table S1. All variables for the main dataset and the questionnaire data set, the categories for categorical variables, their abbreviations, and definitions. 3. Questionnaire Results (components 1–8), Table S2. Component 1 (mouth-related conflict behaviours 1) predictive and trend Bayesian regression model results. Predictive results are shown in bold; Table S3. Component 2 (full body conflict behaviours 1) predictive and trend Bayesian regression model results. Predictive results are shown in bold; Table S4. Component 3 (mouth-related conflict behaviours 2) predictive and trend Bayesian regression model results. Predictive results are shown in bold; Table S5. Component 4 (full body conflict behaviours 2) predictive and trend Bayesian regression model results. Predictive results are shown in bold; Table S6. Component 5 (full body conflict behaviours 3) predictive and trend Bayesian regression model results. Predictive results are shown in bold; Table S7. Component 6 (training-related conflict behaviours) predictive and trend Bayesian regression model results. Predictive results are shown in bold; Table S8. Component 7 (spook-related conflict behaviours) predictive and trend Bayesian regression model results. Predictive results are shown in bold; Table S9. Component 8 (full body conflict behaviours 4) predictive and trend Bayesian regression model results. Predictive results are shown in bold. 4. Discussion of Questionnaire Results (components 1–8), 5. Full Result Output Tables, Table S10. Component 1 (mouth-related conflict behaviours 1) Bayesian Regression Model Results. Predictive/trend results are show in bold; Table S11. Component 2 (full body conflict behaviours 1) Bayesian Regression Model Results. Predictive/trend results are show in bold; Table S12. Component 3 (mouth-related conflict behaviours 2) Bayesian Regression Model Results. Predictive/trend results are show in bold; Table S13. Component 4 (full body conflict behaviours 2) Bayesian Regression Model Results. Predictive/trend results are show in bold; Table S14. Component 5 (full body conflict behaviours 3) Bayesian Regression Model Results. Predictive/trend results are show in bold; Table S15. Component 6 (training-related conflict behaviours) Bayesian Regression Model Results. Predictive/trend results are show in bold; Table S16. Component 7 (spook-related conflict behaviours) Bayesian Regression Model Results. Predictive/trend results are show in bold; Table S17. Component 8 (full body conflict behaviours 4) Bayesian Regression Model Results. Predictive/trend results are show in bold; Table S18. Noseband type Bayesian Regression Model Results. Predictive/trend results are show in bold; 6. Full Questionnaire. References [63,64,65,66,67,68,69,70,71,72,73,74,75] are cited in the Supplementary Materials.
