# Supplementary material for: Influences of Double Versus Snaffle Bridles on Equine Behaviour at Dressage Competitions and Factors That Interact with Their Effect
Source: Animals (Basel). 2025 Jun 17;15(12):1782. doi: 10.3390/ani15121782 (PMC12189879; doi:10.3390/ani15121782)
Supplement: Supplementary file 1 [file animals-15-01782-s001.zip › animals-3531266-supplementary.pdf]

Supplementary Information for:

Influences of Double Versus Snaffle Bridles on Equine Behaviour at Dressage Competitions and Factors That Interact with Their Effect

R. Faithfull, K. Lewis, E. Drury, S. McBride

Corresponding author: Sebastian McBride

Email: sdm@aber.ac.uk; rifkafaithfull@gmail.com

This PDF file includes:

- Model Diagnostics
- Full Variables Table
- Full results section for all predictive and trend results, including results tables for each component
- Discussion of all questionnaire results not included in the paper
- Tables of all result outputs from all Bayesian regression models
- Questionnaire
- References

1. Model Diagnostics

A number of diagnostic techniques were used for each model, to ensure a high level of model fit throughout. The 'summary' argument provided Rhat values for models. Rhat is a measure of chain convergence, with a value of 1.0 denoting complete convergence. Caterpillar plots of the two chains were also created using the 'plot' argument, to provide a further check of chain convergence. In all models, we used Rhat ≤ 1.00 and oscillating caterpillar plots to indicate chain convergence. Posterior prediction plots were then generated using the 'pp_check' argument. Models were accepted if posterior predictions largely picked up on the pattern of the observed y values. Finally, posterior checks of collinearity were undertaken using the 'pairs' argument. Plots were visually examined for collinearity in predictor variables. Although there were some weak associations noted between intercepts and predictor variables in a handful of cases, no strong relationships between pairs of predictor variables were observed in any model.

2. Variables

Table S1. All variables for the main dataset and the questionnaire data set, the categories for categorical variables, their abbreviations, and definitions.

Variable	Categories	Initial	Description
			**Whole dataset categorical data**
Bridle Type	Snaffle Bridle	S	A bridle with only one bit
	Double Bridle	D	A bridle with two bits, a bridoon (snaffle), and a curb bit (often called a Weymouth) which is a leverage bit. The angle of the curb bit is positioned using a curb chain.

Competition Level	Elementary	E	Elementary level - Walk, trot and canter, lengthened strides, lateral movements including leg yield, and collected trot and canter.
	Medium	M	Medium Level - As previous with increased difficulty of lateral movements, extended paces, walk pirouette, and halfpass in trot/canter.
	Advanced Medium	A	Advanced Medium Level- As previous with flying changes.
	Prix St. George (PSG)	P	Prix St. George Level - Small tour - as previous with tempi changes (multiple flying changes), canter pirouettes and increased difficult of movements
	Inter I	I1	Intermediate I Level - Small tour - as previous with increased complexity of movements.
Competition Type	Regional	R	A British Dressage regional competition where riders must qualify to compete. Qualification requires gaining enough points by performing tests at 'typical' competitions (more points are gained by achieving higher scores) and achieving a minimum score. This competition was held outdoors.
	Typical	T	A regular day to day British Dressage competition in which any affiliated rider may enter. This was held in an indoor arena.
Rider Gender	Male	Mr	Riders were male.
	Female	Fr	Riders were female.
Use of Spurs	Spurs	S	The rider wore spurs.
	No Spurs	nS	The rider did not wear spurs.
Use of Ear Bonnet	Ear Bonnet	EB	The horse wore an ear bonnet.
	No Ear Bonnet	nEB	The horse did not wear an ear bonnet.
Snaffle/bridoon cheekpiece type	Loose ring	Lr	The round cheekpiece of the bit freely rotated.
	Eggbutt	Eb	The rounded rings of the cheekpiece were fixed. Provides some pressure on the cheek when turning.
	Hanging cheek	Hc	A lower case 'b'-shaped cheekpiece, which was fixed. The arm attached to the cheekpiece reduces poll pressure at lower rein tensions and increases poll pressure at higher rein tensions.
	D-ring	Dr	The cheekpieces were fixed and D-shaped. Provides more cheek pressure during turning than an eggbutt.
Noseband Type	Cavesson	Cn	A plain noseband that fastened around the upper portion of the nose. It was not possible to distinguish between regular cavessons and crank (Swedish) nosebands, so both were classed under cavesson.
	Flash	Fn	Worn with a cavesson noseband and has an additional lower strap that fastened below the bit under the chin.
	Drop	Dn	A plain noseband that fastened around the lower portion of the nose and fastened below the bit.
	Four-ring-drop	Rn	A drop noseband that had an additional strap positioned under the mandible at the position where the underneath portion of a cavesson noseband would sit. As such, it has a similar effect to the horse wearing both a drop and a cavesson noseband together. The four 'rings' allow for some change in angle of the straps, and therefore this may not be fastened as low on the nose as a traditional drop noseband.
**Whole dataset continuous data**			
Patting Frequency			The number of times the horse was patted between the rider saluting at the end of their test and exiting the arena. Each contact of the hand is counted as a single pat. If patted with 2 hands simultaneously, a single pat is counted for each simultaneous contact of the hands with the horse.
Average nose angle			The average angle of the horses head throughout the test where 0° is vertical and perpendicular to the ground. The average was calculated from measurements taken at 10 second intervals throughout the test.
Test Score			The score awarded to the test by the competition judged. This was a scored by a single judge positioned at "C" at the typical competition

			and an average score across 3 judges positioned at "C", "H", and "B" at the regionals competition.
Angle categories	0° to -10°		The percentage of the test the horse spend with it's head in the angle range of 0° to -10°.
	>-10°		The percentage of the test the horse spend with it's head in the angle range of more than -10°.
**Questionnaire dataset categorical variables**			
Professional bridle fit	Professionally Fitted	PF	The bridle had not been fitted by a professional bit and bridle fitter.
	Not professionally fitted	nPF	The bridle had been fitted by a professional bit and bridle fitter.
Rider maintenance treatments	Regular maintenance treatments	MT	The rider regularly received maintenance treatments such as physiotherapy or chiropractic treatments.
	No maintenance treatments	nMT	The rider did not regularly receive maintenance treatments.
Time spent training in a double bridle	Does not wear double bridle	nDB	The horse did not wear a double bridle.
	Less than half of training rides	LH	The horse wore a double bridle for less than half of training rides at home.
	More than half of training rides	MH	The horse wore a double bridle for more than half of training rides at home.
Time wearing double bridle	Does not wear double bridle	nDB	The horse did not wear a double bridle.
	<3 months	3m	The horse had been ridden in a double bridle for less than 3 months.
	3 months to 1 year	1y	The horse had been ridden in a double bridle for 3 months to 1 year.
	1-2 years	2y	The horse had been ridden in a double bridle for 1-2 years.
	>5 years	5y	The horse had been ridden in a double bridle for more than 5 years.
Rider's highest level trained at home	Elementary/medium	EM	The rider had trained at home up to movements equating that of elementary or medium level.
	Advanced Medium	A	The rider had trained at home up to movements equating that of advanced medium level.
	Prix St. George (PSG)	P	The rider had trained at home up to movements equating that of PSG level.
	Intermediate II (inter 2)	I2	The rider had trained at home up to movements equating that of inter 2 level.
	Grand Prix	G	The rider had trained at home up to movements equating that of grand prix level.
Horse's highest level trained at home	Elementary	E	The horse had trained at home up to movements equating that of elementary level.
	Medium	M	The horse had trained at home up to movements equating that of medium level.
	Advanced Medium	A	The horse had trained at home up to movements equating that of advanced medium level.
	Prix St. George (PSG)	P	The horse had trained at home up to movements equating that of PSG level.
	Grand Prix	G	The horse had trained at home up to movements equating that of grand prix level.
Time spent training before first competing	>6 months	6m	The horse trained for less than 6 months before it first began competing.
	6 months to 1 year	1y	The horse trained for 6 months to 1 year before it first began competing.
	1-2 years	2y	The horse trained for 1-2 years before it first began competing.
	2-5 years	5y	The horse trained for 2-5 years before it first began competing.
Time since last dental examination	<1 month	1m	The horses last examination by an equine dentist was less than 1 month ago.
	1-3 months ago	3m	The horses last examination by an equine dentist was 1-3 months ago.

	3-6 months ago	6m	The horses last examination by an equine dentist was 3-6 months ago.
	6-12 months ago	12m	The horses last examination by an equine dentist was 6-12 months ago.
Bit category	Plain Double jointed snaffle	DJS	A snaffle bit with 2 joints in the mouthpiece.
	Anatomical double jointed snaffle	ADJS	A snaffle bit with 2 joints in the mouthpiece, and a curved shaped mouthpiece that follows the curve of the mouth.
	Eggbutt snaffle	ES	A snaffle bit with eggbutt cheek pieces.
	Straightbar Weymouth double bridle	SW	A double bridle with a straight bar weymouth and a bridoon bit.
	Ported/Myler Weymouth double bridle	PW	A double bridle with a ported weymouth or a myler weymouth (which is ported with a roller) and a bridoon bit.
Frequency of dental examinations	Every 6 months	6m	The horse typically has its teeth examined by an equine dentist every 6 months.
	Every 7.5 months	7.5m	The horse typically has its teeth examined by an equine dentist every 7.5 months.
	Every 12 months	12m	The horse typically has its teeth examined by an equine dentist every 12 months.

3. Questionnaire Results

3.1. Component 1 - Mouth Related Conflict Behaviours 1

All predictive and trend results for component 1 are presented in Table S2.

Table S2. Component 1 (Mouth Related Conflict Behaviours 1) predictive and trend Bayesian regression model results. Predictive results are shown in bold. (D=double bridle, S=snaffle bridle; E=elementary, M=medium; A=advanced medium; P=Prix St. George; I=intermediate I; T=typical competition, R=regional competition; MT=rider receives maintenance treatments, nMT=rider does not receive maintenance treatments; nDB=no double bridle, LH=less than half of training rides, MH=more than half of training rides; 3m=<3 months, 1y=3 month to 1 year, 2y=1-2 years, 5y=>5 years; E=Elementary, M=Medium, A=Advanced Medium, P=Prix St. George, G=Grand Prix; 1m=>1 month, 3m=1-3 months ago, 6m=3-6 months ago, 12m=6-12 months ago; DJS=Double jointed snaffle, ADJS=Anatomical double jointed snaffle, ES=Eggbutt snaffle, SW=Straightbar Weymouth double bridle, PW=Ported/Myler Weymouth double bridle; * denotes category interaction).

Model	Variable	Sample Size	Category Size	Hypothesis	Estimate	Lower CrI	Upper CrI
Main Data Set	Competition Level	118	E=50 M=24 A=8	E>I1	-0.63	-1.34	0.08
		118	P=21 I=15	M>A	-1.19	-2.48	0.07
		118		**I>M**	**1.31**	**0.47**	**2.15**
		118		**M>P**	**-1.02**	**-1.83**	**-0.23**
	Competition Type	118	T=11 R=107	**R>T**	**-1.57**	**-3.03**	**-0.08**
	Bridle Type * Competition Level	118	D*E=11 S*E=39	**S>D*E>M**	**-1.16**	**-2.18**	**-0.15**
		118	D*M=9 S*M=15	**S>D*E>P**	**-2.47**	**-4.44**	**-0.50**
		118	D*A=4 S*A=4	S>D*P>A	2.51	-0.18	5.20
		118	D*P=20 S*P=1	S>D*I1>M	-1.75	-3.80	0.31
		118	D*I=14 S*I=1	**S>D*I1>P**	**-3.06**	**-5.87**	**-0.21**
Questionnaire Rider Maintenance Treatment	Rider Maintenance Treatment	28	MT=20 nMT=8	MT>nMT	0.67	-0.04	1.38
Questionnaire Time Training	Training in Double Bridle	28	nDB=17 LH=5 MH=6	**MH>nDB**	**1.21**	**0.42**	**2.00**
		28		LH>MH	-0.73	-1.55	0.09

in Double Bridle							
Questionnaire Time Wearing Double Bridle	Time Wearing Double Bridle	28	nDB=17 3m=2 1y=1 2y=6 5y=2	**3m>2y**	**-1.62**	**-2.66**	**-0.59**
		28		2y>nDB	1.44	**0.72**	**2.16**
		28		2y>5y	1.03	0.63	-0.01
Questionnaire Horse Highest Level Trained	Horse Highest Level Trained	28	E=4 M=12 A=4 P=4 G=4	G>A	1.11	-0.14	2.37
		28		**E>M**	**0.01**	**0.89**	**0.85**
Questionnaire Time Since Last Dental Examination	Last Dental Examination	28	1m=4 3m=12 6m=5 12m=7	12m>1m	-1.47	-3.07	0.19
		28		**3m>12m**	**1.86**	**0.98**	**2.74**
		28		**6m>12m**	**2.22**	**1.05**	**3.38**
	Bridle Type*Last Dental	28	S*1m=3 D*1m=1	S>D*3m>6m	-0.95	-1.98	0.09
		28	S*3m=6 D*3m=6	**S>D*3m>12m**	**-2.12**	**-3.20**	**-1.03**
		28	S*6m=3 D*6m=2	S>D*6m>12m	-1.17	-2.65	0.29
		28	S*12m=5 D*12m=2	S>D*12m>1m	1.71	-0.13	3.52
Questionnaire Bit Category	Bit Category	26	DJS=7 ADJS=4 ES=4 SW=7 PW=4	**SW>ADJS**	**1.19**	**0.23**	**2.13**
		26		**DJS>SW**	**-0.86**	**-1.53**	**-0.20**
		26		**ES>SW**	**-0.87**	**-1.69**	**-0.04**
		26		**PW>SW**	**-0.76**	**-1.50**	**-0.02**
Behind the Vertical Angle Categories	<-10 degrees	135			-0.0117	-0.0255	0.0022

Competition type predicted component 1 scores (Est.=-1.57, CrIs [-3.03, -0.08]) Component 1 scores were lower at regional competitions than typical competitions. Competition level predicted component 1 with greater component 1 scores at inter 1 (Est.=1.31, CrIs [0.47, 2.15]), and PSG (Est.=-1.02, CrIs [-1.83, -0.23]) levels than at medium level, as well as trends towards higher component 1 scores at advanced medium (Est.=-1.19, CrIs [-2.48, 0.07]) than at medium, and inter 1 than elementary (Est.=-0.63, CrIs [-1.34, 0.08]). The interaction between bridle type and competition level was predictive of component 1. When wearing a snaffle bridle, there were higher scores at medium (Est.=-1.16, CrIs [-2.18, -0.15]) and PSG (Est.=-2.47, CrIs [-4.44, -0.50]) than at elementary, and higher scores at PSG than at inter 1 (Est.=-3.06, CrIs [-5.87, -0.21]). With a snaffle bridle, there were also trends towards higher component 1 scores at PSG than at advanced medium (Est.=2.51, CrIs [-0.18, 5.20]) and at medium than inter 1 (Est.=-1.75, CrIs [-3.80, 0.31]). There was a trend towards time in the <-10° angle group predicting a component 1 with a negative relationship (Est.=0.0106, CrIs [-0.0025, 0.0238]).

The amount of time spent training in a double bridle predicted component 1, with higher component 1 scores for horses ridden in double bridles for more than half of training compared to horses who did not wear double bridles (Est.=1.21, CrIs [0.42, 2.00]). There was also a trend towards horses who wore a double bridle for more than half of training rides having a higher component 1 score than those who wore a double bridle for less than half of training rides (Est.=-0.73, CrIs [-1.55, 0.09]). The length of time since a horse first started wearing a double bridle also predicted component 1 scores. Those who had been wearing a double bridle for 1-2 years had higher component 1 scores than those who did not wear a double bridle (Est.=1.44, CrIs [0.72, 2.16]) or who had been wearing one for less than 3 months (Est.=-1.62m CrIs [-2.66, -0.59]). Those who had been wearing a double bridle for more than 5 years had lower component 1 scores than those who had been wearing a double bridle for 1-2 years (Est.=1.03, CrIs [-0.01, 2.05]). The category of bit the horse was wearing predicted component 1. There were higher component 1 scores for double bridles with straight-bar weymouths than double jointed anatomical snaffles (Est.=1.19, CrIs [0.23, 2.13]), plain double jointed snaffles (Est.=-0.86, CrIs [-1.53, -0.20]), eggbutt snaffles (Est.=-0.87, CrIs [-1.69, -0.04]), and double bridles with ported weymouths (Est.=-0.76, CrIs [-1.50, -0.02]). The time since the horses last dental examination also predicted component 1. Horses who were examined by a dentist 1-3 months ago (Est.=1.86, CrIs [0.98, 2.74]) and 3-6 month ago (Est.=2.22, CrIs [1.05, 3.38]) had higher

component 1 scores than those who had a dental examination 6-12 months ago. There was a trend towards horses whose teeth were examined in the last month having higher component 1 scores than those who were examined 6 months to 1 year ago (Est.=-1.47, CrIs [-3.07, 0.09]). There was a trend towards horses who had trained to a highest level of Grand Prix having a higher component 1 score than those who trained to a highest level of Advanced Medium (Est.=1.11, CrIs [-0.14, 2.37]). The interaction between bridle type and a horse's last dental examination also trended towards being predictive of component 1. For horses wearing a double bridle, those who were examined 1-3 months ago had higher component 1 scores than those examined 12 months ago (Est.=2.12, CrIs [-3.20, -1.03]), and there were trends towards those examined less than a month ago (Est.=1.71, CrIs [-0.13, 3.52]) or 3-6 months ago (Est.=1.17, CrIs [-2.65, 0.29]) having higher component 1 scores than those examined 6-12 months ago. When wearing a snaffle bridle, there was a trend towards those examined 3-6 months ago having higher component 1 scores than those examined 1-3 months ago (Est.=-0.95, CrIs [-1.98, 0.09]). There was a trend towards higher component 1 scores when riders received regular maintenance treatments (Est.=0.67, CrIs [-0.04, 1.38]).

No other variables in the models predicted component 1 (Table S10).

3.2. Component 2 – Full Body Conflict Behaviours 1

All predictive and trend results for component 2 are presented in Table S3.

Table S3. Component 2 (Full Body Conflict Behaviours 1) predictive and trend Bayesian regression model results. Predictive results are shown in bold. (D=double bridle, S=snaffle bridle; E=elementary, M=medium; A=advanced medium; P=Prix St. George; I=intermediate I; EB=ear bonnet, nEB=no ear bonnet; E=Elementary, M=Medium, A=Advanced Medium, P=Prix St. George, G=Grand Prix; DJS=Double jointed snaffle, ADJS=Anatomical double jointed snaffle, ES=Eggbutt snaffle, SW=Straightbar Weymouth double bridle, PW=Ported/Myler Weymouth double bridle; * denotes category interaction).

Model	Variable Name	Sample Size	Category Size	Hypothesis	Estimate	Lower CrI	Upper CrI
Main Data Set Model	Bridle Type * Competition Level	118	D*E=11 S*E=39	**S>D*E>I1**	**-0.90**	**-1.80**	**-0.01**
		118	D*M=9 S*M=15	**S>D*I1>M**	**1.16**	**0.24**	**2.09**
		118	D*A=4 S*A=4 D*P=20 S*P=1 D*I=14 S*I=1	S>D*I1>A	1.17	-0.03	2.36
	Ear Bonnet	118	EB=55 nEB=63	EB>nEB	-0.16	-0.34	0.02
	Test Score	118			-0.03	-0.07	0.00
Questionnaire Horse Highest Level Trained	Horse Highest Level Trained	28	E=4 M=12 A=4 P=4 G=4	E>G	-0.66	-1.32	0.00
		28		E>P	-0.48	-1.04	0.09
		28		G>M	0.42	-0.09	0.94
Questionnaire Bit Category	Bit Category	26	DJS=7 ADJS=4 ES=4 SW=7 PW=4	SW>ADJS	-0.51	-1.03	0.01

The interaction between bridle type and competition level predicted component 2. When wearing a snaffle bridle, inter 1 had higher component 1 scores than elementary (Est.=-0.90, CrIs [-1.80, -0.01]) and medium level (Est.=1.16, CrIs [0.24, 2.09]), and advanced medium trended towards being higher than inter 1 (Est.=1.17, CrIs [-0.03, 2.36]). These differences were not seen in horses wearing double bridles. There was a trend towards horses not wearing an ear bonnet having higher component 2 scores than those who wore an ear bonnet (Est.=-0.16, CrIs [0.34, 0.02]). There was also a trend towards a negative correlation between test scores and component 2 scores, with test scores being lower with higher component 2 scores (Est.=0.03, CrIs [0.07, 0.02]).

The highest level the horse had trained to trended towards predicting component 2. Horses who were trained to Grand Prix at home trended towards higher component 2 scores than those who had

trained to either elementary (Est.=-0.66, CrIs [-1.32, 0.00]) or medium level (Est.=-0.48, CrIs [-1.04, 0.09]). Those who trained to PSG trended towards higher component 2 scores than those who trained to elementary (Est.=-0.48, CrIs [-1.04, 0.09]). The category of bit the horse was wearing trended towards predicting component 2 with higher component 2 scores for those wearing double jointed anatomical snaffles than double bridles with straight-bar weymouths (Est.=-0.51, CrIs [-1.03, 0.01]).

No other variables in the models predicted component 2 (Table S11).

3.3. Component 3 – Mouth-Related Conflict Behaviours 2

All predictive and trend results for component 3 are presented in Table S4.

Table S4. Component 3 (Mouth-Related Conflict Behaviours 2) predictive and trend Bayesian regression model results. Predictive results are shown in bold. (S=snaffle bridle, D=double bridle; nDB=no double bridle, LH=less than half of training rides, MH=more than half of training rides; EM=elementary and medium level, A=advanced medium, P=Prix St. George, I2=intermediate II, G=grand prix; E=Elementary, M=Medium, A=Advanced Medium, P=Prix St. George, G=Grand Prix; 1m=>1 month, 3m=1-3 months ago, 6m=3-6 months ago, 12m=6-12 months ago; 6m=6 monthly examinations, 7.5m=7.5 monthly examinations, 12m=12 monthly examinations; * denotes category interaction)

Model	Variable Name	Sample Size	Category Size	Hypothesis	Estimate	Lower CrI	Upper CrI
Questionnaire Time Wearing Double Bridle	Time Wearing Double Bridle	28	nDB= 3m= 1y= 2y= 5y=	**3m>1y**	**-2.53**	**-4.56**	**-0.50**
		28		**1y>2y**	**2.24**	**0.46**	**3.98**
		28		**1y>5y**	**2.76**	**0.76**	**4.76**
		28		**1y>nDB**	**2.24**	**0.20**	**4.30**
Questionnaire Rider Highest Level Trained	Rider Highest Level Trained	25	EM=4 A=4 P=6 I2=5 G=9	EM>I2	1.15	-0.03	2.31
Questionnaire Horse Highest Level Trained	Horse Highest Level Trained	28	E=4 M=12 A=4 P=4 G=4	**E>A**	**1.80**	**0.40**	**3.22**
		28		**E>G**	**1.62**	**0.29**	**2.96**
		28		**E>M**	**1.59**	**0.66**	**2.53**
		29		E>P	0.95	-0.15	2.06
Questionnaire Time Since Last Dental Examination	Bridle Type*Last Dental	28	S*1m=3 D*1m=1 S*3m=6 D*3m=6 S*6m=3 D*6m=2 S*12m=5 D*12m=2	S>D*6m>12m	-1.97	-4.21	0.31
Questionnaire Dental Examination Frequency	teeth frequency	28	6m=16 7.5m=1 12m=11	**6m>7.5m**	**-2.42**	**-4.14**	**-0.75**
		**25**		**7.5m>12m**	**2.34**	**0.22**	**4.50**

The amount of time since a horse first started wearing a double bridle predicted component 3. Those who had been wearing a double bridle for 3 months to 1 year had higher component 3 scores than those who do not wear a double bridle (Est.=2.24, CrIs [0.20, 4.30]), had been wearing a double bridle for 1-2 years (Est.=2.24, CrIs [0.46, 3.98]), and had been wearing a double bridle for more than 5 years (Est.=2.76, CrIs [0.76, 4.76]). There was a trend towards those who had worn a double bridle for less than 3 months having lower component 3 scores than those who had been wearing one for 3 months to 1 year (Est.=-2.53, CrIs [-4.56, -0.50]). The highest level a rider trained to predicted component 3, with those who had trained to elementary or medium level having a higher component score than those who had trained to inter 2 level (Est.=1.15, CrIs [-0.03, 2.31]). The highest level the horse had trained to predicted component 3. Horses trained to the highest level of elementary had higher component 3 scores than those trained to medium (Est.=1.59, CrIs [0.66, 2.53]), advanced medium (Est.=1.80, CrIs [0.40, 3.22]), and grand prix (Est.=1.62, CrIs [0.29, 2.96]), and there was a trend towards those trained to elementary having higher component 3 scores than those trained to PSG level (Est.=0.95, CrIs [-0.15, 2.06]). The frequency with which a horse's teeth

were examined predicted component 3, with those who were examined every 7.5 months having higher component 3 scores than those who were every 6 (Est.=-2.42, CrIs [-4.14, -0.75]) or 12 months (Est.=2.34, CrIs [0.22, 4.50]). The interaction between bridle type and when the horses last dental examination trended towards predicting component 3. When wearing a double bridle, horses whose teeth were examined 3-6 months ago had higher component 3 scores than those examined 6 months to 1 year ago but when wearing a snaffle bridle, those who were examined 6 months to 1 years ago had higher component 3 scores than 3-6 months ago (Est.=-1.97, CrIs [-4.21, 0.31]).

No other variables in the models predicted component 3 (Table S12).

3.4. Component 4 – Full Body Conflict Behaviours 2

All predictive and trend results for component 4 are presented in Table S5.

Table S5. Component 4 (Full Body Conflict Behaviours 2) predictive and trend Bayesian regression model results. Predictive results are shown in bold. (D=double bridle, S=snaffle bridle; E=elementary, M=medium; A=advanced medium; P=Prix St. George; I=intermediate I; EM=elementary and medium level, A=advanced medium, P=Prix St. George, I2=intermediate II, G=grand prix; 1m=>1 month, 3m=1-3 months ago, 6m=3-6 months ago, 12m=6-12 months ago; DJS=Double jointed snaffle, ADJS=Anatomical double jointed snaffle, ES=Eggbutt snaffle, SW=Straightbar Weymouth double bridle, PW=Ported/Myler Weymouth double bridle; * denotes category interaction).

Model	Variable Name	Sample Size	Category Size	Hypothesis	Estimate	Lower CrI	Upper CrI
Main Data Set Model	Bridle Type * Average Nose Angle	118	S=60 D=58	**S>D**	**0.14**	**0.05**	**0.24**
	Patting Score	118			0.01	-0.00	0.03
	Bridle Type * Competition Level	118<br>118	D*E=11 S*E=39 D*M=9 S*M=15 D*A=4 S*A=4 D*P=20 S*P=1 D*I=14 S*I=1	**E>I1**<br>**E>M**	**2.05**<br>**0.91**	**0.22**<br>**0.00**	**3.87**<br>**1.82**
Questionnaire Rider Highest Level Trained	Rider Highest Level Trained	28	EM=4 A=4 P=6 I2=5 G=9	I2>P	-0.86	-1.78	0.07
Questionnaire Time Since Last Dental Examination	Bridle Type*Last Dental	28<br>28	S*1m=3 D*1m=1 S*3m=6 D*3m=6 S*6m=3 D*6m=2 S*12m=5 D*12m=2	S>D*3m>6m<br>**S>D*6m>1y**	-1.32<br>**1.59**	-2.67<br>**0.04**	0.06<br>**3.14**
Questionnaire Bit Category	Bit Category	26<br>26	DJS=7 ADJS=4 ES=4 SW=7 PW=4	**DJS>ADJS**<br>**DJS>ES**	**-0.74**<br>**-0.69**	**-1.42**<br>**-1.26**	**-0.05**<br>**-0.11**
Behind the Vertical Angle Categories	Bridle type * <-10 degrees	121	S=60 D=61	**S>D**	**-0.0279**	**-0.0502**	**-0.0057**

The interaction between bridle type and the average angle of the horses head predicted component 4 (Est.=0.14, CrIs [0.05, 0.24]). There was a positive relationship between nose angle and component 4 for those wearing a snaffle bridle, whereas this relationship was negative for those wearing a double bridle. The interaction between bridle type and competition level predicted component 4 with higher component 4 scores at elementary than at medium (Est.=0.91, CrIs [0, 1.82]) or inter 1 level (Est.=2.05, CrIs [0.22, 3.87]) when the horse was wearing a snaffle bridle. These differences between competition level were not seen in horses wearing double bridles (Table S4). There was a trend towards the number of times the horse was patted at the end of the test predicting component 4, with those who were patted more having a higher component 4 score (Est.=0.10, CrIs [-0.00, 0.03]). The interaction between the <-10° angle group and bridle type was predictive of

component 4 (Est.=-0.0279, CrIs [-0.0502, -0.0057]) with a negative correlation between the <-10° angle group and component 4 for snaffle bridles and a slight positive correlation for double bridles.

The category of bit the horse was wearing predicted component 4. There were higher component 4 scores for double jointed anatomical snaffles (Est.=-0.74, CrIs [-1.42, -0.05]) and eggbutt snaffles (Est.=-0.69, CrIs [-1.26, -0.11]) compared to plain double jointed snaffles. The interaction between bridle type and when the horse's teeth were last examined predicted component 4. For snaffle bridles, those who were examined 3-6 months ago had higher component 4 scores than those who were examined 6 months to 1 year ago (Est.=-1.32, CrIs [-2.67, 0.06]). There was a trend towards higher component 4 scores for those had their teeth examined 3-6 months ago than 3 months ago (Est.=1.59, CrIs [0.04, 3.14]). These differences between dental examination times were not seen in horses wearing double bridles (Table S4). Horses ridden by riders who had trained to a highest level of inter 2 trended towards having higher component 4 scores than horses whose riders trained to PSG (Est.=-0.86, CrIs[-1.78, 0.07]).

No other variables in the models predicted component 4 (Table S13).

3.5. Component 5 – Fully Body Conflict Behaviours 3

All predictive and trend results for component 5 are presented in Table S6.

Table S6. Component 5 (Fully Body Conflict Behaviours 3) predictive and trend Bayesian regression model results. Predictive results are shown in bold. (D=double bridle, S=snaffle bridle; E=elementary, M=medium; A=advanced medium; P=Prix St. George; I=intermediate I; T=typical competition, R= regional competition; 3m=<3 months, 1y=3 month to 1 year, 2y=1-2 years, 5y=>5 years; E=Elementary, M=Medium, A=Advanced Medium, P=Prix St. George, G=Grand Prix; DJS=Double jointed snaffle, ADJS=Anatomical double jointed snaffle, ES=Eggbutt snaffle, SW=Straightbar Weymouth double bridle, PW=Ported/Myler Weymouth double bridle; * denotes category interaction).

Model	Variable Name	Sample Size	Category Size	Hypothesis	Estimate	Lower CrI	Upper CrI
Main Data Set Model	Competition Level	118	E=50 M=24 A=8 P=21 I=15	**E>A**	**-1.64**	**-2.83**	**-0.45**
		118		**I1>A**	**-1.39**	**-2.53**	**-0.28**
		118		**M>A**	**-1.72**	**-2.95**	**-0.53**
		118		**P>A**	**-1.22**	**-2.31**	**-0.13**
	Bridle Type * Competition Level	118	D*E=11 S*E=39	S>D*E>A	1.36	-0.17	2.89
		118	D*M=9 S*M=15	S>D*M>A	1.51	-0.10	3.10
		118	D*A=4 S*A=4	S>D*P>A	2.75	-0.20	5.27
		118	D*P=20 S*P=1 D*I=14 S*I=1	S>D*R>T	-1.32	-2.87	0.23
	**Bridle Type * Competition Type**	118	S*T=8 D*T=3 S*R=52 D*R=55	**S>D*R>T**	**-1.32**	**-2.87**	**0.23**
Questionnaire Time Wearing Double Bridle	Time Wearing Double Bridle	29	nDB=17 3m=2 1y=1 2y=6 5y=2	3m>5y	-1.07	-2.26	0.13
		29		1y>5y	-1.43	-2.90	0.04
		28		**5y>nDB**	**1.30**	**0.23**	**2.40**
		28		**2y>5y**	**-1.27**	**-2.24**	**-0.30**
Questionnaire Horse Highest Level Trained	Horse Highest Level Trained	28	E=4 M=12 A=4 P=4 G=4	**E>G**	**-0.99**	**-1.96**	**-0.02**
		28		**G>M**	**1.15**	**0.39**	**1.91**
				G>P	0.79	-0.12	1.69
		28		**G>A**	**1.31**	**0.28**	**2.35**
Questionnaire Bit Category	Bit Category	26	DJS=7 ADJS=4 ES=4 SW=7 PW=4	DJS>ES	0.68	-0.05	1.42
		26		ES>PW	-0.87	-1.75	0.03
Behind the vertical angle categories	<-10 degrees	135			0.0106	- 0.0025	0.0238

Component 5 scores were higher at advanced medium level than at other levels (A-E (Est.=-1.64, CrIs [-2.83, -0.45]), A-I (Est.=-1.39, CrIs [-2.53, -0.28]), A-M (Est.=-1.72, CrIs [-2.95, -0.53]), A-P (Est.=-1.22,

CrIs [-2.31, -0.13]). The interaction between bridle type and competition level predicted component 5. Horses at PSG level trended towards greater component 5 scores than those at advanced medium, when wearing a snaffle bridle (Est.=2.75, CrIs [0.20, 5.27]). There were trends towards higher component 5 scores for horses at advanced medium level than at elementary (Est.=1.36, CrIs [-0.17, 2.89]), or medium (Est.=1.51, CrIs [-0.10, 3.10]) when wearing a double bridle. There was a trend towards the interaction between bridle type and competition type predicting component 5 with higher component 5 scores at regional competitions when horses were wearing a double bridle (Est.=-1.32, CrIs [-2.87, 0.23]). There was a trend towards time in the <-10° angle group predicting component 5 scores with a positive relationship (Est.=0.0106, CrIs [-0.0025, 0.0238]).

The length of time since a horse first started wearing a double bridle predicted component 5. Horses who had been wearing a double bridle for more than 5 years had higher factor 5 scores than either horses who did not wear a double bridle (Est.=1.30, CrIs [0.23, 2.40]) or had been wearing a double bridle for 1-2 years (Est.=-1.27, CrIs [-2.24, -0.3]). There was a trend towards horses who had been wearing a double bridle for more than 5 years having higher factor 5 scores than those who had been wearing a double bridle for either less than 3 months (Est.=-1.07, CrIs [-2.26, 0.13]) or 3 months to 1 year (Est.=-1.43, CrIs [-2.90, 0.04]). The highest level a horse had trained to predicted component 5, with those who had trained to grand prix level having higher component 5 scores that those who had trained to elementary (Est.=-0.99, CrIs [-1.96, -0.002]), medium (Est.=1.15, CrIs [0.39, 1.91]), or advanced medium (Est.=1.31, CrIs [0.28, 2.35]) and trending towards being higher than those who trained to PSG level (Est.=0.79, CrLs[-0.12, 1.69]. There was a trend towards the bit category the horse was wearing predicting component 5. There were higher component 5 scores for those wearing plain double jointed snaffle bits (Est.=0.68, CrIs [-0.05, 1.42]) and double bridles with ported/myler weymouths (Est.=-0.87, CrIs [-1.75, 0.03]) than eggbutt snaffles.

No other variables in the models predicted component 5 (Table S14).

3.6. Component 6 – Training-Related Conflict Behaviours

All predictive and trend results for component 6 are presented in Table S7.

Table S7. Component 6 (Training-Related Conflict Behaviours) predictive and trend Bayesian regression model results. Predictive results are shown in bold. (D=double bridle, S=snaffle bridle; T=typical competition, R=regional competition; Fr=female rider, Mr=male rider; E=elementary, M=medium; A=advanced medium; P=Prix St. George; I=intermediate I; Lr=loose ring cheekpiece, Eb=eggbutt cheekpiece, Dr=D-ring cheekpiece, Hc=hanging cheek cheekpiece; nDB=no double bridle, LH=less than half of training rides, MH=more than half of training rides; 3m=<3 months, 1y=3 month to 1 year, 2y=1-2 years, 5y=>5 years; EM=elementary and medium level, A=advanced medium, P=Prix St. George, I2=intermediate II, G=grand prix; 6m=>6 months, 1y=6 months to 1 year, 2y=1-2 years, 5y=2-5 years; 1m=>1 month, 3m=1-3 months ago, 6m=3-6 months ago, 12m=6-12 months ago; DJS=Double jointed snaffle, ADJS=Anatomical double jointed snaffle, ES=Eggbutt snaffle, SW=Straightbar Weymouth double bridle, PW=Ported/Myler Weymouth double bridle; * denotes category interaction)

Model	Variable Name	Sample Size	Category Size	Hypothesis	Estimate	Lower CrI	Upper CrI
Main Data Set Model	Bridle Type	118	S=60 D=58	**S>D**	**-2.52**	**-4.64**	**-0.38**
	Competition Type	118	T=11 R=107	**R>T**	**-2.86**	**-4.29**	**-1.43**
	Rider Gender	118	Fr= 97 Mr=21	Mr>Fr	0.47	-0.08	1.01
	Competition Level	118	E=50 M=24 A=8	**I1>M**	**1.00**	**0.19**	**1.79**
		118	P=21 I=15	**I1>P**	**0.73**	**0.16**	**1.29**
	Bridle Type * Competition Type	118	S*T=8 D*T=3 S*R=52 D*R=55	**S>D*R>T**	**3.07**	**1.43**	**4.68**

	Snaffle Cheek Piece	118	Lr=98 Eb=18 Dr=1 Hc=1	Hc>Dr	2.43	-0.40	5.23
	Bridle Type * Competition Level	118	D*E=11 S*E=39	**S>D*I1>A**	-2.25	-4.81	0.31
		118	D*M=9 S*M=15	S>D*I1>P	-1.93	-3.92	0.06
		118	D*A=4 S*A=4 D*P=20 S*P=1 D*I=14 S*I=1	**S>D*I1>M**	**-1.93**	**-3.92**	**0.06**
Questionnaire Time Training in Double Bridle	Training in Double Bridle	28	nDB=17 LH=5 MH=6	**MH>nDB**	**1.29**	**0.15**	**2.41**
Questionnaire Time Wearing Double Bridle	Time Wearing Double Bridle	28	nDB=17 3m=2	3m>2y	-1.29	-2.88	0.30
		28	1y=1 2y=6 5y=2	5y>nDB	0.18	-1.57	1.93
		28		2y>5y	1.40	-0.18	2.99
Questionnaire Rider Highest Level Trained	Rider Highest Level Trained	28	EM=4 A=4 P=6 I2=5 G=9	G>A	1.30	-0.24	2.85
Questionnaire Time Horse Trained Before Starting to Compete	Training Before Competing	28	6m=6 1y=6 2y=12 5y=4	**2y>5y**	**-1.58**	**-3.00**	**-0.15**
Questionnaire Bit Category	Bit Category	26	DJS=7 ADJS=4 ES=4	SW>ADJS	1.29	-0.13	2.71
		26	SW=7 PW=4	**DJS>SW**	**-1.70**	**-2.65**	**-0.75**
		26		**ES>SW**	**-1.95**	**-3.15**	**-0.76**
Behind the Vertical Angle Categories	<-10 degrees	135			-0.0114	-0.0255	0.0027

Bridle type predicted higher component 6 scores with a double bridle than a snaffle bridle (Est.=-2.52, CrIs [-4.64, -0.38]). Competition level predicted component 6 with higher component 6 scores at inter 1 than medium (Est.=1.00, CrIs [0.19, 1.79]) and PSG level (Est.=0.73, CrIs [0.34, 0.16]). The interaction between bridle type and competition level trended towards predicting component 6. Advanced medium level had higher scores than inter 1 when wearing a snaffle bridle (Est.=-2.25, CrIs [-4.81, 0.31]), inter 1 had higher component 6 scores than PSG level, and there was a trend towards medium having higher component 6 scores than inter 1 when wearing a snaffle bridle (Est.=-1.93, CrIs [-3.92, 0.06]). There was a trend towards horses with male riders having higher component 6 scores (Est.=0.47, CrIs [-0.08, 1.01]). Competition type predicted higher component 6 scores at the typical competition (Est.=-2.86, CrIs [-4.29, -1.43]). The interaction between bridle type and competition type predicted component 6 scores with higher scores at typical competitions than regionals when the horse is wearing a double bridle (Est.=1.43, CrIs [4.68, 3.07]). The cheekpiece type of the snaffle bit or bridoon bit within the double bridle predicted component 6 scores. Horses who wore hanging-cheek bits had higher component 6 scores than those who wore loose ring bits (Est.=2.25, CrIs [0.46, 4.05]). There was a trend towards horses with a hanging-cheek having higher component 6 scores than those with a D-ring (Est.=2.43, CrIs [-0.40, 5.23]). There was a trend towards time in the <-10° angle category predicting a component 6 with a negative relationship (Est.=-0.0114, CrIs [-0.0255, 0.0027]).

The category of bit the horse was wearing predicted component 6. There were higher component 6 scores for double bridles with straightbar weymouths than plain double jointed snaffles (Est.=-1.70, CrIs [-2.65, -0.75]) or eggbutt snaffles (Est.=-1.95, CrIs [-3.15, -0.76]). There was a trend towards higher component 6 scores for double bridles with straightbar weymouths than double jointed anatomical snaffles (Est.=1.29, CrIs [-0.13, 2.71]). The amount of time horses spent training in double bridles predicted component 6 with those training in a double bridle for more than half of training rides having higher component 6 scores than those who did not wear double bridles (Est.=1.29, CrIs [0.15, 2.41]). The length of time since a horse first started wearing a double bridle predicted component 6. Horses who had been wearing a double bridle for 1-2 years had higher component 6 scores than those who did not wear double bridles (Est.=1.58, CrIs[0.46, 2.69]). There was a trend towards horses who had been wearing a double bridle for 1-2 years having higher component 6

scores than those who had either been wearing a double bridle for less than 3 months (Est.=-1.29, CrIs[-2.88, 0.30]) or more than 5 years (Est.=1.40, CrIs [-0.18, 2.99]). The highest level a horse's rider had trained to trended towards predicting component 6, with those whose riders had trained to grand prix level having higher component 6 scores than those who's riders trained to advanced medium (Est.=1.30, CrIs [-0.24, 2.85]). The length of time horses spent training before they first started competing trended towards predicting component 6, with those who trained for 2-5 years having higher component 6 scores than those who trained for 1-2 years (Est.=-1.58, CrIs [-3.00, -0.15]).

No other variables in the models predicted component 6 (Table S15).

3.7. Component 7 – Spook-Related Conflict Behaviours

All predictive and trend results for component 7 are presented in Table S8.

Table S8. Component 7 (Spook-Related Conflict Behaviours) predictive and trend Bayesian regression model results. Predictive results are shown in bold. (S=snaffle bridle, D=double bridle; T=typical competition, R=regional competition; E=elementary, M=medium, A=advanced medium, P=Prix St. George, G=Grand Prix; *denotes category interaction).

Model	Variable Name	Sample Size		Hypothesis	Estimate	Lower CrI	Upper CrI
Main Data Set Model	Bridle Type * Competition Type	118	S*T=8 D*T=3 S*R=52 D*R=55	S>D*R>T	-1.95	-3.60	0.30
	Bridle Type	118	D=58 S=60	S>D	2.19	-0.01	4.37
Questionnaire Horse Highest Level Trained	Horse Highest Level Trained	29	E=4 M=12 A=4 P=4 G=4	**E>P**	**-1.53**	**-2.94**	**-0.15**
		29		**M>P**	**-1.24**	**-2.36**	**-0.12**
		28		P>A	1.39	-0.29	3.12

The interaction between bridle type and competition type predicted component 7 with higher component 7 scores at typical competitions than regionals when wearing a snaffle bridle (Est.=-1.95, CrIs [-3.60, -0.30]). There was a very strong trend of bridle type predicting component 7 with snaffles having higher component 7 scores (Est.=2.19, CrIs [-0.01, 4.37]).

The highest level a horse had trained to predicted component 7 with higher component 7 scores for horses who were trained up to PSG level than those who had trained up to elementary (Est.=-1.53, CrIs [-2.94, -0.15]) and medium (Est.=-1.24, CrIs [-2.36, -0.12]), and a trend towards higher component 7 scores at PSG than advanced medium (Est.=1.39, CrIs [-0.29, 3.12]).

No other variables in the models predicted component 7 (Table S16).

3.8. Component 8 – Full Body Conflict Behaviours 4

All predictive and trend results for component 8 are presented in Table S9.

Table S9. Component 8 (Full Body Conflict Behaviours 4) predictive and trend Bayesian regression model results. Predictive results are shown in bold. (Lr=loose ring cheekpiece, Eb=eggbutt cheekpiece, Dr=D-ring cheekpiece, Hc=hanging cheek cheekpiece; D=double bridle, S=snaffle bridle; EM=elementary and medium level, A=advanced medium, P=Prix St. George, I2=intermediate II, G=grand prix; 6m=>6 months, 1y=6 months to 1 year, 2y=1-2 years, 5y=2-5 years; 1m=>1 month, 3m=1-3 months ago, 6m=3-6 months ago, 12m=6-12 months ago; DJS=Double jointed snaffle, ADJS=Anatomical double jointed snaffle, ES=Eggbutt snaffle, SW=Straightbar Weymouth double bridle, PW=Ported/Myler Weymouth double bridle; * denotes category interactions).

Model	Variable Name	Sample Size	Category Size	Hypothesis	Estimate	Lower CrI	Upper CrI
Main Data Set Model	Snaffle cheek piece	118	Lr=98 Eb=18 Dr=1 Hc=1	**Eb>Lr**	**0.92**	**0.45**	**1.39**
Questionnaire Rider Highest Level Trained	Rider Highest Level Trained	29	EM=4 A=4 P=6 I2=5 G=9	**I2>P**	**-1.47**	**-2.85**	**-0.08**
Time Horse Trained Before Starting to Compete (TBC)	Training Before Competing	28	6m=6 1y=6 2y=12 5y=4	**1y>2y**	**1.21**	**0.08**	**2.35**
Questionnaire Time Since Last Dental Examination	Bridle Type*Last Dental	28<br>28	S*1m=3 D*1m=1 S*3m=6 D*3m=6 S*6m=3 D*6m=2 S*12m=5 D*12m=2	S>D*3m>1m<br>S>D*6m>1m	-2.21<br>**2.83**	-4.44<br>**0.26**	0.03<br>**5.40**
Questionnaire Bit Category	Bit Category	26<br>26<br>26<br>26	DJS=7 ADJS=4 ES=4 SW=7 PW=4	**ES>ADJS**<br>**DJS>ES**<br>**ES>PW**<br>**ES>SW**	**2.40**<br>**-1.69**<br>**1.87**<br>**1.88**	**0.95**<br>**-2.77**<br>**0.59**<br>**0.77**	**3.88**<br>**-0.61**<br>**3.15**<br>**2.98**
Behind the Vertical Angle Categories	Bridle type * <-10 degrees	135	D=70 S=65	S>D	0.0215	-0.0001	0.0432

The cheekpiece type of the snaffle bit or bridoon bit within the double bridle predicted component 8 scores, with eggbutt cheekpieces predicting higher component 8 scores than loose ring cheekpieces (Est.=0.92, CrIs [0.45, 1.39]). There was a trend towards the interaction between time in the <-10° angle group and bridle type being predictive for component 8 (Est.=0.0215, CrIs [-0.0001, 0.0432]) with a positive correlation between time in the <-10° angle group and component 8 for snaffle bridles and a negative correlation for double bridles.

The highest level a horse's rider had trained to predicted component 8, with those who's riders trained to PSG having higher component 8 scores than those who's riders trained to Inter 2 (Est.=-1.47, CrIs [-2.85, -0.08]). The length of time horses spent training before they first started competing predicted component 8, with those who trained for 6 months to 1 year having higher component 8 scores than those who trained for 1-2 years (Est.=-1.21, CrIs [0.08, 2.35]). The category of bit the horse was wearing predicted component 8. Eggbutt snaffles predicted higher component 8 scores than all other bit categories (ES-DJA Est.=2.40, CrIs [0.95, 3.88], ES-DJS Est.=-1.69, CrIs [-2.77, -0.61], ES-SBPM Est.=1.87, CrIs [0.59, 3.15], ES-SBSW Est.=1.88, CrIs [0.77, 2.98]). The interaction between when a horse's last dental examination was, and the bridle type predicted component 8. When wearing a snaffle, horses who had their teeth examined 3-6 months ago had higher component 8 scores than horses whose were examined either 1-3 months ago (Est.=-2.21, CrIs [-4.44, 0.03]), or 6 months to 1year ago (Est.=2.38, CrIs [0.26, 5.40]).

No other variables in the models predicted component 8 (Table S17).

4. Discussion of questionnaire results

4.1. Component 1 –Mouth Related Conflict Behaviours 1

Component 1 scores were predicted by the highest level a horse had trained to at home. There was a trend towards those who trained to grand prix having higher component 1 scores than those who trained to advanced medium. At higher levels of test, movements are more technically and athletically demanding resulting in greater use of the double bridle that may lead to an increase in mouth-related conflict behaviours, particularly if the horse is not sufficiently prepared for this level [1]. The length of time since the horse first started wearing a double bridle also predicted the level of

component 1 scores. Those who wore a double bridle for 1-2 years predicted higher component 1 scores compared to those who did not wear a double bridle or who had been wearing one for less than 3 months. These effects may reflect poorly fitted double bridles, for example, if they are too large for the oral cavity and prevent the horse closing its mouth fully. This may lead to restricted breathing [2], compression of the tongue [3], and oral lesions [2,4]. The group who wore a double bridle for more than 5 years was too small to draw conclusions from but further research that explores the longer-term effects (behavioural and physiological) of using a double bridle is required.

Dental examinations were also predictive of component 1 scores with higher scores for those who were examined by a dentist 1-3 months ago and 3-6 months ago than those examined 6-12 months ago. This suggests that horses that have not had recent dental issues are less likely to show mouth-related conflict behaviours. Interestingly, the interaction between the time of the horse's last dental examination and bridle type was also predictive of component 1. The results for double bridles mirrored that of the overall results for dental examinations, however, snaffle bridles differed in that there were higher component 1 scores when horses were examined 3-6 months ago compared to a more recent examination of 1-3 months ago. This result is more difficult to explain, especially due to limited sample sizes but may reflect increased use of a double bridle masking the effects of dentistry on component 1 scores. Increased mobility of the bit of a snaffle bridle in the mouth [5,6] could be less likely to aggravate any recent dental treatment.

4.2. Component 2 – Full Body Conflict Behaviours 1

The bit category results showed higher component 2 scores for anatomical double-jointed snaffles than double bridles with straight bar weymouths, mirroring the whole data set result of higher component 7 scores for spook-related conflict behaviours with a snaffle bridle compared to a double bridle as presented for component 7 in the main paper. The correct use of a double bridle should also lead to increased engagement of the horse's body in self-carriage [7,8] which would reduce the ability to spook due to the level of overall body muscle engagement. The bit within the snaffle bridle may also allow more freedom of full body behavioural expression as the bridle is less restrictive, and the bit is able to move more in the horse's mouth. This differs from the bridoon bit of a double bridle, which is positioned above the curb bit and therefore it's movement within the mouth is reduced [5].

Horses who trained at home to the highest level of grand prix predicted higher component 2 scores than those who trained to elementary or medium levels and there was a trend towards those who trained to PSG level having higher component 2 scores than those who trained to elementary. Horses trained to a higher level will not only be more physically engaged but also more mentally engaged in their ridden work. Horses trained to lower levels may be more easily distracted by their surrounding during a test compared to those trained to higher levels. This could cause a larger and therefore more obvious spooking-type response to auditory stimuli for higher level horses as they are less likely to be processing the sound in the context of their surroundings. This could cause a startle response leading to an increase in component 2 behaviours [9].

4.3. Component 3 – Mouth Related Conflict Behaviours 2

The highest level a rider had trained to at home predicted higher component 3 scores for training to elementary/medium level than to inter 1, suggesting that rider skill is influential over component 3 behaviours, as also suggested for the main data set results showing decreased component 1 scores for more skilled riders for mouth-related conflict behaviours 1. Riders who had trained to higher levels may have had greater hand stability [10] and therefore more refined use of the reins resulting

in a more stable tongue-bridle connection [11]. Similar results were seen for the highest level the horse had trained to at home, predicting higher component 3 scores for those who trained to elementary than medium, advanced medium, and grand prix and a trend towards predicting higher component 3 scores when trained to elementary than PSG level. This suggests that a lack of strength and resulting tension in the body could impact tongue and jaw twisting behaviours [12]. This link between strength and oral behaviour could be explained by the connection of the masseter muscles at the mandible (which facilitate jaw twisting behaviours), through the superficial ventral line of facia and muscle (sternomandibular muscles, thoracic muscles, hip extension muscles, hind leg flexion muscles) to the movement of the hind leg [13], which is highly important for the posture required in dressage.

The use of the bit also potentially impacted component 3 behaviours through the amount of time the horse had worn a double bridle, with those horses that wore a double bridle for 3 months to 1 year having higher component 3 scores compared to horses who wore the double bridle for longer. Group sizes within these double bridle training durations were however small and thus further research with larger sample sizes are required to assess the effect of double bridle use over time.

The interaction of bridle type and the length of time since the horse's last dental examination was predictive of component 3. Horses who wore a double bridle trended towards higher component 3 scores when their teeth were examined 3-6 month ago compared to 6-12 months ago, whereas this was reversed for those wearing a snaffle bridle. Due to the possible impact of increased use of a double bridle on component 3, it is possible that the effect of double bridles on component 3 scores masked the behavioural symptoms of dental discomfort as suggested in component 1. Dental issues may increase component 3 behaviours due to the bit pressing the tongue and cheek tissues against sharp areas of the teeth [14] or the jaw twisting in an attempt to relieve areas that may be sore or rubbing, being further exacerbated by the double bridle reducing the horse's ability to escape any sore points due to the increased space it takes up in the oral cavity [3]. This may account for double bridle wearers showing higher component 3 scores with a more recent dental examination than horses wearing snaffle bridles.

4.4. Component 4 – Full Body Conflict Behaviours 2

The highest level a rider had trained to at home was predictive of higher component 4 scores for those who trained to PSG compared to those who trained to inter 2. Component 4 varied across levels of rider training without a clear overall direction which highlights the importance of the horse's strength and correctness of their training [15]. Overall component 4 data suggests that in order to reduce component 4 behaviours, both horse and rider combined must be of a high skill and performance level, whereas, for example, a skilled rider on a younger or weaker horse may not be able to reduce losses of balance to the degree they could on a more conditioned equivalent.

The interaction between bridle type and the horse's last dental examination was also predictive of component 4. Snaffle bridles predicted greater component 4 scores for 3-6 months post examination than 6-12 months and a trend towards higher component 4 scores for 3-6 months post examination than 1-3 months. The time frame of 3-6 month post routine examination is a time period in which more serious dental issues may re-arise for horses prone to them. In addition, the greater level of mobility of the snaffle in the mouth could exacerbate any untreated dental issues [16] in a way that affects the horses whole body movement [13]. With the double bridle, there was a slight increase in component 4 since the time of the last dental examination, but this was much reduced in comparison to the snaffle bridle. This suggests that the double bridle is more fixed within the mouth

and does not interact with the teeth to the same extent in a way that might lead to increased hollowness and balance related issues.

4.5. Component 5 – Full Body Conflict Behaviours 3

The highest level a horse had trained to at home was predictive of component 5 scores, with those who trained up to grand prix having higher component 5 scores than those who trained to elementary, medium, or advanced medium. Those training to higher levels may have been wearing a double bridle for longer. Double bridles have been linked to a physiological stress response [17], which could explain higher instances of fear related conflict behaviours. The higher demand of movements at these levels may also increase tension in the horses body [1].

Bit category was predictive of lower component 5 scores for eggbutt snaffles than other bit types, which could potentially relate to a greater stabilising effect of the cheekpiece [20] reducing the likelihood of behaviours like spontaneous change of gait.

4.6. Component 6 – Training-Related Conflict Behaviours

The bit category data showed higher component 6 scores associated with double bridles with straight bar weymouths than any snaffle bit type. As tongue exposure is negatively weighted for component 6, this suggests the straight bar double bridle could be connected to a reduction in tongue exposure in comparison to snaffle bits. A possible explanation for this is that double jointed snaffle bit puts the majority of its pressure over the tongue due to the two joints allowing the bit to conform to the shape of the tongue. The double bridle, however, spreads its pressure over a greater number of areas and therefore its pressure won't be as concentrated on the tongue, and may, therefore, reduce tongue behaviour in comparison to double jointed snaffles. The amount of time since a horse first started wearing a double bridle was also predictive of component 6 scores, with higher component 6 scores for those who wore a double bridle for 1-2 years than those who did not wear a double bridle or who had worn one for less than 3 months, which could reflect possible adaptations to long term use of a double bridle in the case of tongue exposure, and mirrors the effect of higher competition levels having higher component 6 scores, as well as double bridles having higher component 6 scores than snaffle bridles, so could suggest the length of time a horse has been wearing a double bridle may contribute to these effects.

The training of horse and rider also affected component 6. Horses who trained for 2-5 years before they first began competing had higher component 6 scores than those who trained for 1-2 years. This suggests that horses who are naturally more balanced due to their breeding and confirmation [21], and therefore require less initial training, have lower component 6 scores. The highest level a rider trained to at home was also predictive of component 6 scores, following a very similar pattern across levels to that of competition level. Horses who trained to grand prix level predicted higher component 6 scores than those who trained to advanced medium. This is contrary to expectation as more skilled riders should make less mistakes due to their superior ability to balance the horse [22]. Therefore, it appears that the horse's strength and ability is more influential than that of the rider's skill and even a highly trained rider is not able to mask these mistakes in a young or inexperienced horse who is weaker as they bring them through the levels.

4.7. Component 7 – Spook-Related Conflict Behaviours

Component 7 was predicted by the highest level the horse had trained to at home. Those who trained to PSG level predicted higher component 7 scores than those who trained to elementary or medium and trended towards predicting higher component 7 scores than those who trained to

advanced medium. This could have related to the temperament of horses that train to higher levels. With the ability to perform high level competition movements often comes a high energy temperament and for some horses this can manifest as greater reactivity to environmental stimuli [9]. We may not see this temperament affecting horses training to grand prix level as they likely have a larger amount of competition experience and therefore may be less sensitive to the environment.

4.8. Component 8 – Full Body Conflict Behaviours 4

The eggbutt bit type was predictive of higher component 8 scores than any other bit type. The eggbutt is more likely to be chosen for horses who require more support in their balance due to the greater stability of the bit, pressure on the horses cheek, and it's more direct translation of the rein pressure to the mouth [20], hence an association between this bit and higher component 8 behaviours.

The interaction between bridle type and the time of horses last dental examination was also predictive of component 8. When wearing a double bridle there was little variation in component 8 scores across the different dental examination time points, suggesting that double bridles did not interact with dental issues to influence component 8 behaviours. However, when wearing a snaffle bridle, those who were examined 3-6 months ago predicted higher component 8 scores than those examined 6-12 months ago and there was a trend towards those examined 3-6 months ago predicting higher component 8 scores than those who were examined 1-3 months ago. As previously discussed for other components, dental issues may be more likely to arise 3-6 months post examination and they can affect the horses whole body movement through tension in the jaw and facial muscles and their connection to muscle chains with the horse's body [13,23–25]. As also discussed, this may be more prominent in a snaffle bridle due to the bits higher degree of mobility in the horse's mouth. The curb bit decreases the mobility of the bridoon bit in comparison to the bit of a snaffle bridle as the bridoon sits above and rests on the curb bit [5,6]. This may make a snaffle bridle more likely to aggravate dental issues as it could contact sore areas or even the teeth, particularly if it is not correctly fitted [3,8] leading to inconsistencies in rein contact, loss of balance, tripping, or irregular head nodding.

The amount of time a horse trained before it began competing was predictive of component 8 scores, being higher for those who trained for 6 months to 1 year than those who trained for 1-2 years. Horses who started competing after less than 6 months of training may have been more naturally talented or naturally balanced due to their confirmation and breeding [21]. Those who trained longer than 1 year may have built more strength and balance compared to those who trained 6 months to 1 year, leading to less component 8 behaviours. The highest level the rider had trained to at home was also predictive of component 8 scores, with higher scores for those who trained to PSG than those who trained to inter 2. Over half of the riders in the PSG category were riding horses who were wearing a snaffle and had a dental examination in the last 3-6 months so this cross over due to the small dataset could have influenced this result.

5. Full Results Output

Table S10. Component 1 (mouth-related conflict behaviours 1) Bayesian Regression Model Results. Predictive/trend results are show in bold. (S=Snaffle Bridle; D=Double Bridle; E=Elementary, M=Medium, A=Advanced Medium, P=Prix St. George, I=Intermediate I; R=Regionals, T=Typical; Mr=Male, Fr=Female; EB=Ear Bonnet, nEB=No Ear Bonnet; Lr=Loose ring, Eb=Eggbutt, Hc=Hanging cheek, Dr=D-ring; PF=Professionally Fitted, nPF=Not professionally fitted, MT=Regular maintenance treatments, nMT=No maintenance treatments; nDB=Does not wear double bridle, LH=Less than half of training rides, MH=More

than half of training rides; nDB=Does not wear double bridle, 3m=<3 months, 1y=3 month to 1 year, 2y=1-2 years, 5y=>5 years; EM=Elementary or medium, A=Advanced Medium, P= Prix St. George, I2=Intermediate II, G=Grand Prix; E=Elementary, M=Medium, A=Advanced Medium, P=Prix St. George, G=Grand Prix; 6m=>6 months, 1y=6 months to 1 year, 2y=1-2 years, 5y=2-5 years; 1m=>1 month, 3m=1-3 months ago, 6m=3-6 months ago, 12m=6-12 months ago; DJS=Double jointed snaffle, ADJS=Anatomical double jointed snaffle, ES=Eggbutt snaffle, SW=Straightbar Weymouth double bridle, PW=Ported/Myler Weymouth double bridle; 6m=Every 6 months, 7.5m=Every 7.5 months, 12m=Every 12 months; *denotes category interaction).

Model	Variable Name	Sample Size	Category Size	Hypothesis	Estimate	Lower CrI	Upper CrI
Main Data Set	Bridle Type	118	S=60 D=58	S>D	-1.44	-3.64	0.75
	Average Nose Angle	118			0.01	-0.06	0.08
	**Competition Level**	118	E=50 M=24 A=8 P=21 I=15	E>M	0.68	-0.22	1.58
		118		E>A	-0.51	-1.74	0.75
		118		E>P	-0.34	-1.01	0.33
		118		**E>I1**	**-0.63**	**-1.34**	**0.08**
		118		**M>A**	**-1.19**	**-2.48**	**0.07**
		118		**M>P**	**-1.02**	**-1.83**	**-0.23**
		118		P>A	-0.17	-1.31	0.98
		118		**I1>M**	**1.31**	**0.47**	**2.15**
		118		I1>A	0.12	-1.06	1.31
		118		I1>P	0.28	-0.31	0.88
	**Competition Type**	118	T=11 R=107	**R>T**	**-1.57**	**-3.03**	**-0.08**
	Rider Gender	118	Fr=97 Mr=21	Mr>Fr	0.24	-0.31	0.80
	Ear Bonnet	118	nEB=63 EB=55	nEB>EB	0.02	-0.38	0.42
	Snaffle Cheek Piece	118	Dr=1 Eb=18 Hc=1 Lr=98	Eb>Dr	0.08	-1.98	2.14
		118		Hc>Dr	-0.70	-3.62	2.23
		118		Lr>Dr	0.11	-1.91	2.14
		118		Eb>Hc	0.78	-1.09	2.64
		118		Eb>Lr	-0.03	-0.49	0.43
		118		Hc>Lr	-0.81	-2.66	1.03
	Patting Score	118			-0.01	-0.02	0.01
	Test Score	118			-0.05	-0.13	0.03
	Bridle Type * Average Nose Angle	118	S=60 D=58	S>D	0.01	-0.10	0.12
	Bridle Type * Competition Type	118	S*T=8 D*T=3 S*R=52 D*R=55	S>D*R>T	1.17	-0.51	2.82
	**Bridle Type * Competition Level**	118	S*E=39 D*E=11 S*M=15 D*M=9 S*A=4 D*A=4 S*P=1 D*P=20 S*I=1 D*I=14	**S>D*E>M**	**-1.16**	**-2.18**	**-0.15**
		118		S>D*E>A	0.04	-1.59	1.69
		118		**S>D*E>P**	**-2.47**	**-4.44**	**-0.50**
		118		S>D*E>I1	0.59	-1.39	2.56
		118		S>D*M>A	1.20	-0.54	2.94
		118		S>D*M>P	-1.31	-3.35	0.72
		118		**S>D*P>A**	**2.51**	**-0.18**	**5.20**
		118		**S>D*I1>M**	**-1.75**	**-3.80**	**0.31**
		118		S>D*I1>A	-0.55	-3.16	2.09
		118		**S>D*I1>P**	**-3.06**	**-5.87**	**-0.21**
Questionnaire Bridle Professional Fit	Bridle Type	28	S=17 D=11	S>D	-0.59	-1.32	0.14
	Bridle Professional Fit	28	PF=8 nPF=20	PF>nPF	0.69	-0.40	1.76
	Bridle Type * Bridle Professional Fit	28	S*PF=5 S*nPF=12 D*PF=3 D*nPF=8	S>D*PF>nPF	-0.36	-1.72	0.99
	Competition Type	28	T=10 R=18	R>T	-1.27	-2.09	-0.45
	Competition Level	28	E=15 M=8 A=1 P=2 I=2	E>A	0.83	-0.80	2.45
		28		M>A	0.46	-1.13	2.04
		28		P>A	1.36	-0.72	3.43
		28		I1>A	2.05	-0.01	4.14
	Bridle Type	28	S=17 D=11	S>D	-0.29	-0.99	0.41

Questionnaire Rider Maintenance Treatment	**Rider Maintenance Treatment**	28	MT=20 nMT=8	**MT>nMT**	**0.67**	**-0.04**	**1.38**
	Competition Type	28	R=18 T=10	R>T	-1.10	-1.78	-0.41
	Competition Level	28	E=15 M=8 A=1 P=2 I=2	E>A	0.41	-1.19	2.01
		28		M>A	0.14	1.38	1.65
		28		P>A	1.15	-0.80	3.11
		28		I1>A	1.84	-0.12	3.81
Questionnaire Time Training in Double Bridle	**Training in Double Bridle**	28	nDB=17 LH=5 MH=6	LH>nDB	0.48	-0.35	1.31
		28		**MH>nDB**	**1.21**	**0.42**	**2.00**
		28		**LH>MH**	**-0.73**	**-1.55**	**0.09**
Questionnaire Time Wearing Double Bridle	Time Wearing Double Bridle	28	nDB=17 3m=2 1y=1 2y=6 5y=2	3m>nDB	-0.18	-1.33	0.95
		28		3m>1y	-0.76	-2.35	0.80
		28		3m>2y	**-1.62**	**-2.66**	**-0.59**
		28		3m>5y	-0.59	-1.85	0.68
		28		1y>nDB	0.58	-0.99	2.17
		28		1y>2y	-0.86	-2.22	0.52
		28		1y>5y	0.17	-1.39	1.72
		28		**2y>nDB**	**1.44**	**0.72**	**2.16**
		28		**2y>5y**	**1.03**	**-0.01**	**2.05**
		28		5y>nDB	0.41	-0.75	1.54
Questionnaire Rider Highest Level Trained	Bridle Type	28	S=17 D=11	S>D	-0.63	-1.51	0.26
	Rider Highest Level Trained	28	EM=4 A=4 P=6 I2=5 G=9	EM>A	0.58	-0.66	1.85
		28		G>A	0.51	-0.70	1.73
		28		I2>A	0.32	-0.89	1.58
		28		P>A	0.71	-0.36	1.77
		28		EM>G	0.07	-0.90	1.04
		28		EM>I2	0.25	-0.69	1.20
		28		EM>P	-0.13	-1.01	0.76
		28		G>I2	0.19	-0.77	1.14
		28		G>P	-0.20	-1.16	0.77
		28		I2>P	-0.38	-1.28	0.52
	Competition Type	28	T=10 R=18	R>T	-1.08	-1.92	-0.23
	Competition Level	28	E=15 M=8 A=1 P=2 I=2	E>A	0.61	-1.37	2.57
		28		I1>A	1.96	-0.47	4.44
		28		M>A	0.41	-1.47	2.32
		28		P>A	1.27	-1.18	3.72
Questionnaire Horse Highest Level Trained	Bridle Type	28	S=17 D=11	S>D	-0.71	-1.51	0.09
	**Horse Highest Level Trained**	28	E=4 M=12 A=4 P=4 G=4	E>A	0.60	-0.67	1.88
		28		**G>A**	**1.11**	**-0.14**	**2.37**
		28		M>A	0.59	-0.41	1.60
		28		P>A	0.33	-0.89	1.55
		28		E>G	-0.52	-1.70	0.68
		28		**E>M**	**0.01**	**0.89**	**0.85**
		28		E>P	0.27	-0.73	1.27
		28		G>M	0.52	-0.40	1.45
		28		G>P	0.78	-0.31	1.88
		28		M>P	0.26	-0.55	1.08
Questionnaire Time Horse Trained Before Starting to Compete	Bridle Type	28	S=17 D=11	S>D	-0.21	-1.27	0.85
	Training Before Competing	28	6m=6 1y=6 2y=12 5y=4	1y>6m	0.48	-0.48	1.45
		28		2y>6m	-0.09	-0.96	0.80
		28		5y>6m	0.54	-0.68	1.73
		28		1y>2y	0.57	-0.19	1.33
		28		1y>5y	-0.06	-1.14	1.03
		28		2y>5y	-0.63	-1.76	0.49
	Competition Type	28	T=10 R=18	R>T	-1.02	-1.78	-0.25
	Competition Level	28	E=15 M=8 A=1 P=2 I=2	E>A	1.20	-0.54	3.00
		28		I1>A	2.79	0.51	5.12
		28		M>A	0.97	-0.82	2.83
		28		P>A	2.10	-0.21	4.42
Questionnaire Time Since Last Dental Examination	Bridle Type	28	S=17 D=11	S>D	-0.45	-1.66	0.78
	**Last Dental Examination**	28	1m=4 3m=12 6m=5 12m=7	3m>1m	0.39	-0.82	1.63
		28		6m>1m	0.75	-0.57	2.08

		28		**12m>1m**	**-1.47**	**-3.07**	**0.19**
		28		**3m>12m**	**1.86**	**0.98**	**2.74**
		28		**6m>12m**	**2.22**	**1.05**	**3.38**
		28		3m>6m	-0.35	-1.11	0.39
	Competition Type	28	T=10 R=18	R>T	-0.56	-1.29	0.18
	Competition Level	28	E=15 M=8 A=1 P=2 I=2	E>A	0.34	-1.11	1.76
		28		I1>A	2.57	0.68	4.45
		28		M>A	0.21	-1.15	1.54
		28		P>A	1.89	-0.05	3.77
	**Bridle Type*Last Dental**	28	S*1m=3 D*1m=1 S*3m=6 D*3m=6 S*6m=3 D*6m=2 S*12m=5 D*12m=2	**S>D*3m>6m**	**-0.95**	**-1.98**	**0.09**
		28		**S>D*3m>12m**	**-2.12**	**-3.20**	**-1.03**
		28		**S>D*6m>12m**	**-1.17**	**-2.65**	**0.29**
		28		S>D*3m>1m	-0.41	-1.82	0.95
		28		S>D*6m>1m	0.54	-1.08	2.13
		28		**S>D*12m>1m**	**1.71**	**-0.13**	**3.52**
Questionnaire Dental Examination Frequency	Bridle Type	28	S=17 D=11	S>D	-0.44	-1.27	0.36
	Dental Examination Frequency	28	6m=16 7.5m=1 12m=11	6m>12m	0.21	-0.47	0.90
		28		7.5m>12m	0.54	-1.28	2.35
		28		6m>7.5m	-0.33	-1.72	1.07
	Competition Type	28	T=10 R=18	R>T	-1.14	-1.91	-0.34
	Competition Level	28	E=15 M=8 A=1 P=2 I=2	E>A	0.73	-1.00	2.41
		28		I1>A	2.27	0.07	4.38
		28		M>A	0.34	-1.37	2.00
		28		P>A	1.59	-0.60	3.70
Questionnaire Bit Category	**Bit Category**	26	DJS=4 ADJS=7 ES=4 PW=4 SW=7	DJS>ADJS	0.33	-0.61	1.26
		26		ES>ADJS	0.32	-0.78	1.42
		26		PW>ADJS	0.43	-0.71	1.54
		26		**SW>ADJS**	**1.19**	**0.23**	**2.13**
		26		DJS>ES	0.01	-0.79	0.80
		26		DJS>PW	-0.11	-0.92	0.71
		26		**DJS>SW**	**-0.86**	**-1.53**	**-0.20**
		26		ES>PW	-0.11	-1.06	0.85
		26		**ES>SW**	**-0.87**	**-1.69**	**-0.04**
		26		**PW>SW**	**-0.76**	**-1.50**	**-0.02**
	Competition Type	26	T=8 R=18	R>T	-0.89	-1.70	-0.09
	Competition Level	26	E=14 M=7 A=1 P=2 I=2	E>A	0.63	-1.13	2.38
		26		I1>A	2.08	-0.03	4.19
		26		M>A	0.23	-1.45	1.90
		26		P>A	1.39	-0.72	3.55
Behind the Vertical Angle Categories	Bridle Type	135	S=65 D=70	N>Y	-0.1411	-1.3528	1.0802
	**<-10 degrees**	135			**-0.0117**	**-0.0255**	**0.0022**
	-10-0 degrees	135			-0.0019	-0.0129	0.0166
	Competition Type	135	T=13 R=122	R>T	-0.5025	-1.0995	0.1050
	Competition Level	135	E=53 M=27 A=9 P=27 I=19	E>A	-0.5399	-1.2518	0.1647
		135		E>I1	0.3115	-0.5023	1.1255
		135		E>M	-0.2892	-1.0328	0.4466
		135		E>P	0.0045	-0.7740	0.7719
	Bridle Type * <-10 degrees	135	S=65 D=70	S>D	0.0138	-0.0076	0.0355
	Bridle Type * -10-0 degrees	135	S=65 D=70	S>D	-0.0036	-0.0240	0.0167

Table S11. Component 2 (Full Body Conflict Behaviours 1) Bayesian Regression Model Results. Predictive/trend results are show in bold. (S=Snaffle Bridle; D=Double Bridle; E=Elementary, M=Medium, A=Advanced Medium, P=Prix St. George, I=Intermediate I; R=Regionals, T=Typical; Mr=Male, Fr=Female; EB=Ear Bonnet, nEB=No Ear Bonnet; Lr=Loose ring, Eb=Eggbutt, Hc=Hanging cheek, Dr=D-ring; PF=Professionally Fitted, nPF=Not professionally fitted, MT=Regular maintenance treatments, nMT=No maintenance treatments; nDB=Does not wear double bridle, LH=Less than half of training rides, MH=More than half of training rides; nDB=Does not wear double bridle, 3m=<3 months, 1y=3 month to 1 year, 2y=1-2 years, 5y=>5 years; EM=Elementary or

medium, A=Advanced Medium, P= Prix St. George, I2=Intermediate II, G=Grand Prix; E=Elementary, M=Medium, A=Advanced Medium, P=Prix St. George, G=Grand Prix; 6m=>6 months, 1y=6 months to 1 year, 2y=1-2 years, 5y=2-5 years; 1m=>1 month, 3m=1-3 months ago, 6m=3-6 months ago, 12m=6-12 months ago; DJS=Double jointed snaffle, ADJS=Anatomical double jointed snaffle, ES=Eggbutt snaffle, SW=Straightbar Weymouth double bridle, PW=Ported/Myler Weymouth double bridle; 6m=Every 6 months, 7.5m=Every 7.5 months, 12m=Every 12 months; *denotes category interaction).

Model	Variable Name	Sample Size	Category Size	Hypothesis	Estimate	Lower CrI	Upper CrI
Main Data Set	Bridle Type	118	S=60 D=58	S>D	0.28	-0.69	1.28
	Average Nose Angle	118			0.00	-0.03	0.04
	Competition Level	118	E=50 M=24 A=8 P=21 I=15	E>A	-0.23	-0.79	0.34
		118		I1>A	-0.18	-0.71	0.36
		118		M>A	-0.00	-0.58	0.58
		118		P>A	-0.10	-0.62	0.41
		118		E>I1	-0.05	-0.37	0.27
		118		E>M	-0.23	-0.62	0.17
		118		E>P	-0.13	-0.43	0.17
		118		I1>M	-0.17	-0.54	0.19
		118		I1>P	-0.08	-0.34	0.18
		118		M>P	0.10	-0.26	0.45
	Competition Type	118	T=11 R=107	R>T	0.28	-0.37	0.94
	Rider Gender	118	Fr=97 Mr=21	Mr>Fr	-0.02	-0.27	0.23
	**Ear Bonnet**	118	nEB=63 EB=55	**F>nF**	**-0.16**	**-0.34**	**0.02**
	Snaffle Cheek Piece	118	Dr=1 Eb=18 Hc=1 Lr=98	Eb>Dr	0.27	-0.66	1.19
		118		Hc>Dr	0.66	-0.66	0.96
		118		Lr>Dr	0.26	-0.65	1.19
		118		Eb>Hc	-0.39	-1.23	0.47
		118		Eb>Lr	0.01	-0.20	0.22
		118		Hc>Lr	0.40	-0.44	1.23
	Patting Score	118			-0.00	-0.01	0.00
	**Test Score**	118			**-0.03**	**-0.07**	**0.00**
	Bridle Type * Average Nose Angle	118	S=60 D=58	S>D	-0.03	-0.07	0.02
	**Bridle Type * Competition Level**	118	S*E=39 D*E=11 S*M=15 D*M=9 S*A=4 D*A=4 S*P=1 D*P=20 S*I=1 D*I=14	S>D*E>A	0.26	-0.46	0.99
		118		**S>D*I1>A**	**1.17**	**-0.03**	**2.36**
		118		S>D*M>A	0.00	-0.78	0.77
		118		S>D*P>A	0.60	-0.62	1.81
		118		**S>D*E>I1**	**-0.90**	**-1.80**	**-0.01**
		118		S>D*E>M	0.26	-0.18	0.71
		118		S>D*E>P	-0.34	-1.22	0.55
		118		**S>D*I1>M**	**1.16**	**0.24**	**2.09**
		118		S>D*I1>P	0.56	-0.70	1.83
		118		S>D*M>P	-0.60	-1.51	0.31
	Bridle Type * Competition Type	118	S*T=8 D*T=3 S*R=52 D*R=55	S>D*R>T	-0.51	-1.26	0.21
Questionnaire Bridle Professional Fit	Bridle Type	25	S=14 D=11	S>D	0.43	-0.27	1.12
	Bridle Professional Fit	25	PF=7 nPF=18	PF>nPF	-0.12	-0.87	0.63
	Ear Bonnet	25	EB=15 nEB=10	EB>nEB	-0.31	-0.92	0.31
	Competition Level	25	E=13 M=8 A=0 P=2 I=2	I1>E	0.25	-0.62	1.10
		25		M>E	-0.15	-0.60	0.31
		25		P>E	0.42	-0.46	1.27
	Test Score	25			-0.03	-0.09	0.04
	Bridle Type * Bridle Professional Fit	25	S*PF=5 S*nPF=12 D*PF=3 D*nPF=8	S>D*PF>nPF	0.19	-0.99	1.34
Questionnaire Rider Maintenance Treatment	Bridle Type	25	S=14 D=11	S>D	0.48	-0.04	1.00
	Rider Maintenance Treatment	25	MT=19 nMT=6	MT>nMT	-0.03	-0.63	0.55

	Ear Bonnet	25	EB=15 nEB=10	EB>nEB	-0.38	-0.85	0.10
	Competition Level	25	E=13 M=8 A=0 P=2 I=2	I1>E	0.22	-0.57	1.01
		25		M>E	-0.16	-0.62	0.30
		25		P>E	0.39	-0.40	1.19
	Test Score	25			-0.02	-0.09	0.04
Questionnaire Time Training in Double Bridle	Training in Double Bridle	28	nDB=17, LH=5 MH=6	LH>nDB	-0.14	-0.62	0.33
		28		MH>nDB	-0.29	-0.74	0.16
		28		LH>MH	0.14	-0.33	0.61
Questionnaire Time Wearing Double Bridle	Time Wearing Double Bridle	28	Ndb=17 3m=2 1y=1 2y=6 5y=2	3m>nDB	-0.37	-1.08	0.34
		28		1y>nDB	-0.16	-1.12	0.80
		28		2y>nDB	-0.30	-0.76	0.15
		28		5y>nDB	0.15	-0.56	0.88
		28		3m>1y	-0.21	-1.17	0.75
		28		3m>2y	-0.07	-0.71	0.57
		28		3m>5y	-0.53	-1.31	0.25
		28		1y>2y	0.14	-0.70	0.99
		28		1y>5y	-0.31	-1.25	0.63
		28		2y>5y	-0.46	-1.10	0.19
Questionnaire Rider Highest Level Trained	Bridle Type	25	S=14 D=11	S>D	0.57	-0.10	1.24
	Rider Highest Level Trained	25	EM=3 A=4 P=5 I2=4 G=9	EM>A	-0.17	-1.05	0.69
		25		G>A	0.16	-0.91	1.20
		25		I2>A	-0.23	-1.07	0.61
		25		P>A	0.21	-0.58	1.01
		28		EM>G	-0.33	-1.10	0.44
		28		EM>I2	0.06	-0.62	0.74
		28		EM>P	-0.38	-1.04	0.29
		28		G>I2	0.39	-0.34	1.13
		28		G>P	-0.05	-0.74	0.65
		28		I2>P	-0.44	-1.07	0.19
	Ear Bonnet	25	EB=15 EB=10	EB>nEB	-0.52	-1.05	0.02
	Competition Level	25	E=13 M=8 A=0 P=2 I=2	I1>E	0.07	-0.84	0.98
		25		M>E	-0.25	-0.76	0.27
		25		P>E	0.23	-0.70	1.17
	Test Score	25			-0.04	-0.13	0.05
Questionnaire Horse Highest Level Trained	Bridle Type	28	S=17 D=11	S>D	0.54	0.07	1.02
	**Horse Highest Level Trained**	28	E=4 M=12 A=4 P=4 G=4	E>A	-0.25	-0.93	0.44
		28		**E>G**	**-0.66**	**-1.32**	**0.00**
		28		**E>P**	**-0.48**	**-1.04**	**0.09**
		28		G>A	0.41	-0.28	1.12
		28		M>A	-0.01	-0.56	0.52
		28		P>A	0.23	-0.45	0.91
		28		E>M	-0.24	-0.69	0.22
		28		**G>M**	**0.42**	**-0.09**	**0.94**
		28		G>P	0.19	-0.42	0.79
		28		M>P	-0.24	-0.71	0.23
	Ear Bonnet	28	EB=17 nEB=11	EB>nEB	-0.33	-0.75	0.09
Questionnaire Time Horse Trained Before Starting to Compete	Bridle Type	25	S=14 D=11	S>D	0.50	-0.39	1.39
	Training Before Competing	25	6m=4 1y=5 2y=12 5y=4	1y>6m	-0.12	-0.80	0.56
		25		2y>6m	0.27	-0.39	0.94
		25		5y>6m	0.44	-0.42	1.31
		28		1y>2y	-0.39	-0.90	0.11
		28		1y>5y	-0.56	-1.32	0.19
		28		2y>5y	-0.17	-1.04	0.70
	Ear Bonnet	25	EB=4 nEB=10	EB>nEB	-0.32	-0.81	0.18
	Competition Level	25	E=13 M=8 A=0 P=2 I=2	I1>E	0.30	-0.72	1.29
		25		M>E	-0.28	-0.72	0.16
		25		P>E	0.46	-0.52	1.45
	Test Score	25			-0.04	-0.11	0.03
Questionnaire Time Since Last Dental Examination	Bridle Type	25	S=14 D=11	S>D	0.95	-0.54	2.42
	Last Dental Examination	25	1m=3 3m=11 6m=5 12m=6	3m>1m	0.07	-1.41	1.54
		25		6m>1m	0.23	-1.28	1.75
		25		12m>1m	0.01	-1.82	1.82
		28		3m>6m	-0.15	-1.16	0.85
		28		3m>12m	0.06	-1.10	1.24
		28		6m>12m	0.22	-1.23	1.67
		28		S>D*3m>6m	-0.05	-1.19	1.10

	**Bridle Type*Last Dental**	28	S*1m=3 D*1m=1	S>D*3m>12m	-0.09	-1.47	1.32
		28	S*3m=6 D*3m=6	S>D*6m>12m	-0.03	-1.66	1.57
		25	S*6m=3 D*6m=2	**S>D*3m>1m**	**-0.51**	**-2.12**	**0.10**
		25	S*12m=5	S>D*6m>1m	-0.45	-2.28	1.36
		25	D*12m=2	**S>D*12m>1m**	**-0.42**	**2.62**	**1.74**
	Ear Bonnet	25	EB=15 nEM=10	EB>nEB	-0.38	1.07	0.31
	Competition Level	25	E=13 M=9 A=0	I1>E	0.33	-0.94	1.64
		25	P=2 I=2	M>E	-0.08	-0.70	0.55
		25		P>E	0.10	-0.77	1.84
	Test Score	25			-0.01	-0.11	0.11
Questionnaire Dental Examination Frequency	Bridle Type	25	S=14 D=11	S>D	0.53	-0.08	1.13
	Dental Examination Frequency	25	6m=15 7.5m=1	6m>12m	-0.14	-0.63	0.34
		25	12m=9	7.5m>12m	0.33	-0.88	1.53
		28		6m>7.5m	-0.46	-1.39	0.50
	Ear Bonnet	25	EB=15 nEB=10	EB>nEB	-0.42	-0.90	0.07
	Competition Level	25	E=13 M=8 A=0	I1>E	0.26	-0.60	1.10
		25	P=2 I=2	M>E	-0.10	-0.55	0.35
		25		P>E	0.43	-0.40	1.28
	Test Score	25			-0.01	-0.08	0.06
Questionnaire Bit Category	**Bit Category**	26	DJS=4 ADJS=7	DJS>ADJS	-0.32	-0.84	0.18
		26	ES=4 PW=4	ES>ADJS	-0.24	-0.83	0.36
		26	SW=7	PW>ADJS	-0.38	-0.99	0.24
		26		**SW>ADJS**	**-0.51**	**-1.03**	**0.01**
		26		DJS>ES	-0.09	-0.52	0.34
		26		DJS>PW	0.06	-0.38	0.50
		26		DJS>SW	0.18	-0.17	0.54
		26		ES>PW	0.15	-0.37	0.67
		26		ES>SW	0.27	-0.18	0.72
		26		PW>SW	0.12	-0.28	0.52
	Competition Type	26	T=8 R=18	R>T	0.38	-0.05	0.81
	Competition Level	26	E=14 M=7 A=1	E>A	-1.06	-2.03	-0.11
		26	P=2 I=2	I1>A	-0.84	-1.99	0.32
		26		M>A	-0.64	-1.56	0.27
		26		P>A	-0.66	-1.82	0.49
Behind the Vertical Angle Categories	Bridle Type	121	S=60 D=61	S>D	0.1136	-0.4725	0.6993
	<-10 degrees	121			-0.0017	-0.0082	0.0048
	-10-0 degrees	121			0.0029	-0.0041	0.0100
	Bridle Type * <-10 degrees	121	S=60 D=61	S>D	0.0068	-0.0031	0.0167
	Bridle Type * -10-0 degrees	121	S=60 D=61	S>D	-0.0021	-0.0118	0.0077
	Ear Bonnet	121	EB=57 nEB=64	EB>nEB	-0.1140	-0.2750	0.0469
	Competition Level	121	E=50 M=25 A=8	E>A	-0.0666	-0.4087	0.2678
		121	P=23 I=15	E>I1	0.0255	-0.3704	0.4227
		121		E>M	0.0010	-0.3538	0.3490
		121		E>P	0.0815	-0.2944	0.4527
	Test Score	121			-0.0394	-0.0708	-0.0079

Table S12. Component 3 (mouth-related conflict behaviours 1) Bayesian Regression Model Results. Predictive/trend results are show in bold. (S=Snaffle Bridle; D=Double Bridle; E=Elementary, M=Medium, A=Advanced Medium, P=Prix St. George, I=Intermediate I; R=Regionals, T=Typical; Mr=Male, Fr=Female; EB=Ear Bonnet, nEB=No Ear Bonnet; Lr=Loose ring, Eb=Eggbutt, Hc=Hanging cheek, Dr=D-ring; PF=Professionally Fitted, nPF=Not professionally fitted, MT=Regular maintenance treatments, nMT=No maintenance treatments; nDB=Does not wear double bridle, LH=Less than half of training rides, MH=More than half of training rides; nDB=Does not wear double bridle, 3m=<3 months, 1y=3 month to 1 year, 2y=1-2 years, 5y=>5 years; EM=Elementary or medium, A=Advanced Medium, P= Prix St. George, I2=Intermediate II, G=Grand Prix; E=Elementary, M=Medium, A=Advanced Medium, P=Prix St. George, G=Grand Prix; 6m=>6 months, 1y=6 months to 1 year, 2y=1-2 years, 5y=2-5 years; 1m=>1 month, 3m=1-3 months ago, 6m=3-6 months ago, 12m=6-12 months ago; DJS=Double jointed snaffle, ADJS=Anatomical double jointed snaffle,

ES=Eggbutt snaffle, SW=Straightbar Weymouth double bridle, PW=Ported/Myler Weymouth double bridle; 6m=Every 6 months, 7.5m=Every 7.5 months, 12m=Every 12 months; *denotes category interaction).

Model	Variable Name	Sample Size	Category Size	Hypothesis	Estimate	Lower CrI	Upper CrI
Main Data Set	Bridle Type	118	S=60 D=58	S>D	-0.89	-3.35	1.53
	Average Nose Angle	118			0.03	-0.04	0.11
	Competition Level	118	E=50 M=24 A=8 P=21 I=15	E>M	0.06	-0.93	1.05
		118		E>A	-0.97	-2.38	0.42
		118		E>P	-0.34	-1.08	0.40
		118		E>I1	-0.22	-1.02	0.57
		118		M>A	-1.03	-2.47	0.38
		118		M>P	-0.40	-1.27	0.48
		118		P>A	-0.64	-1.92	0.65
		118		I1>M	0.28	-0.64	1.21
		118		I1>A	-0.75	-2.06	0.56
		118		I1>P	-0.12	-0.77	0.53
	Competition Type	118	T=11 R=107	R>T	0.29	-1.33	1.93
	Rider Gender	118	Fr=97 Mr=21	Mr>Fr	-0.39	-1.02	0.23
	Ear Bonnet	118	nEB=63 EB=55	F>nF	0.05	-0.40	0.49
	Snaffle Cheek Piece	118	Dr=1 Eb=18 Hc=1 Lr=98	Eb>Dr	-0.03	-2.34	2.28
		118		Eb>Hc	0.35	-1.71	2.44
		118		Eb>Lr	-0.27	-0.78	0.24
		118		Hc>Dr	-0.38	-3.66	2.87
		118		Hc>Lr	-0.62	-2.68	1.41
		118		Lr>Dr	0.23	-2.06	2.51
	Patting Score	118			-0.00	-0.02	0.02
	Test Score	118			0.01	-0.08	0.09
	Bridle Type * Average Nose Angle	118	S=60 D=58	S>D	-0.01	-0.13	0.11
	Bridle Type * Competition Type	118	S*T=8 D*T=3 S*R=52 D*R=55 S*T=8 D*T=3 S*R=52 D*R=55	S>D*R>T	-0.10	-1.95	1.73
	Bridle Type * Competition Level	118	S*E=39 D*E=11 S*M=15 D*M=9 S*A=4 D*A=4 S*P=1 D*P=20 S*I=1 D*I=14	S>D*E>M	-0.06	-1.18	1.08
		118		S>D*E>A	0.89	-0.93	2.74
		118		S>D*E>P	0.72	-1.53	3.00
		118		S>D*E>I1	0.69	-1.54	2.90
		118		S>D*M>A	0.95	-0.97	2.84
		118		S>D*M>P	0.78	-1.54	3.08
		118		S>D*P>A	0.17	-2.95	3.28
		118		S>D*I1>M	-0.74	-3.05	1.58
		118		S>D*I1>A	0.20	-2.76	3.20
		118		S>D*I1>P	0.04	-3.18	3.28
Questionnaire Bridle Professional Fit	Bridle Type	28	S=17 D=11	S>D	-0.24	-1.18	0.70
	Bridle Professional Fit	28	PF=8 nPF=20	PF>nPF	0.33	-1.02	1.72
	Bridle Type * Bridle Professional Fit	28	S*PF=5 S*nPF=12 D*PF=3 D*nPF=8	S>D*PF>nPF	0.61	-1.11	2.34
Questionnaire Rider Maintenance Treatment	Bridle Type	28	S=17 D=11	S>D	0.27	-0.64	1.19
	Rider Maintenance Treatment	28	MT=20 nMT=8	MT>nMT	0.68	-0.31	1.67
Questionnaire Time Training in Double Bridle	Training in Double Bridle	28	nDB=17 LH=5 MH=6	LH>nDB	0.11	-0.96	1.18
		28		MH>nDB	0.01	-1.00	1.01
		28		LH>MH	0.10	-0.96	1.17
Questionnaire Time Wearing Double Bridle	**Time Wearing Double Bridle**	28	nDB=12 3m=3 1y=1 2y=6 5y=2	3m>nDB	-0.29	-1.76	1.17
		29		3m>1y	**-2.53**	**-4.56**	**-0.50**
		28		3m>2y	-0.30	-1.64	1.05
		28		3m>5y	0.23	-1.38	1.83
		28		1y>nDB	**2.24**	**0.20**	**4.30**
		28		1y>2y	**2.24**	**0.46**	**3.98**

		28		1y>5y	**2.76**	**0.76**	**4.76**
		28		2y>nDB	0.00	-0.92	0.94
		28		2y>5y	0.52	-0.78	1.85
		28		5y>nDB	-0.52	-1.97	0.94
Questionnaire Rider Highest Level Trained	Bridle Type	25	S=17 D=11	S>D	0.16	-1.01	1.33
	**Rider Highest Level Trained**	25	EM=4 A=4 P=6 I2=5 G=9	EM>A	1.17	-0.31	2.62
		25		EM>P	0.78	-0.37	1.94
		25		**EM>I2**	**1.15**	**-0.03**	**2.31**
		25		EM>G	0.49	-0.72	1.70
		25		P>A	0.39	-0.98	1.77
		25		G>A	0.68	-0.77	2.14
		25		I2>A	0.02	-1.37	1.44
		25		I2>P	-0.36	-1.42	0.70
		25		G>P	0.30	-0.95	1.56
		25		G>I2	0.66	-0.53	1.84
Questionnaire Horse Highest Level Trained	Bridle Type	28	S=17 D=11	S>D	-0.51	-1.39	0.39
	**Horse Highest Level Trained**	28	E=4 M=12 A=4 P=4 G=4	**E>M**	**1.59**	**0.66**	**2.53**
		28		**E>A**	**1.80**	**0.40**	**3.22**
		29		**E>P**	**0.95**	**-0.15**	**2.06**
		28		**E>G**	**1.62**	**0.29**	**2.96**
		28		M>A	0.20	-0.89	1.31
		28		M>P	-0.64	-1.54	0.26
		28		P>A	0.84	-0.48	2.17
		28		G>M	-0.03	-1.05	1.00
		28		G>A	0.18	-1.23	1.55
		28		G>P	-0.67	-1.89	0.56
Questionnaire Time Horse Trained Before Starting to Compete	Bridle Type	25	S=17 D=11	S>D	0.17	-0.85	1.19
	Training Before Competing	25	6m=6 1y=6 2y=12 5y=4	1y>6m	0.11	-1.17	1.40
		28		1y>2y	0.14	-0.82	1.09
		28		1y>5y	-0.42	-1.67	0.81
		25		2y>6m	-0.03	-1.14	1.09
		28		2y>5y	-0.56	-1.83	0.70
		25		5y>6m	0.53	-1.03	2.09
Questionnaire Time Since Last Dental Examination	Bridle Type	25	S=17 D=11	S>D	0.44	-2.10	2.98
	Last Dental Examination	25	1m=4 3m=12 6m=5 12m=7	3m>1m	0.34	-2.00	2.69
		25		3m>6m	-0.89	-2.39	0.59
		25		3m>12m	0.55	-0.92	2.04
		25		6m>1m	1.24	-1.46	3.93
		25		6m>12m	1.44	-0.38	3.26
		25		12m>1m	-0.20	-2.89	2.49
	**Bridle Type*Last Dental**	25	S*1m=3 D*1m=1 S*3m=6 D*3m=6 S*6m=3 D*6m=2 S*12m=5 D*12m=2	S>D*3m>1m	-0.86	-3.67	1.97
		28		**S>D*3m>6m**	0.57	-1.40	2.54
		28		S>D*3m>12m	-1.40	-3.24	0.44
		25		S>D*6m>1m	-1.43	-4.64	1.81
		28		**S>D*6m>12m**	**-1.97**	**-4.21**	**0.31**
		25		S>D*12m>1m	0.53	-2.58	3.65
Questionnaire Dental Examination Frequency	Bridle Type	25	S=17 D=11	S>D	0.13	-0.69	0.95
	**Dental Examination Frequency**	25	6m=16 7.5m=1 12m=11	6m>12m	-0.08	-0.88	0.71
		25		**6m>7.5m**	**-2.42**	**-4.14**	**-0.75**
		25		**7.5m>12m**	**2.34**	**0.22**	**4.50**
Questionnaire Bit Category	Bit Category	26	DJS=4 ADJS=7 ES=4 PW=4 SW=7	DJS>ADJS	1.19	-0.37	2.77
		26		ES>ADJS	0.27	-1.54	2.10
		26		PW>ADJS	0.29	-1.53	2.15
		26		SW>ADJS	1.05	-0.54	2.64
		26		DJS>ES	0.92	-0.39	2.23
		26		DJS>PW	0.90	-0.43	2.24
		26		DJS>SW	0.14	-0.93	1.21
		26		ES>PW	-0.02	-1.60	1.54
		26		ES>SW	-0.78	-2.12	0.58
		26		PW>SW	-0.76	-1.96	0.44
	Competition Type	26	T=8 R=18	R>T	0.03	-1.30	1.35
	Competition Level	26	E=14 M=7 A=1 P=2 I=2	E>A	0.21	2.73	3.16
		26		M>A	-0.68	-3.45	2.07
		26		I>A	0.00	-3.52	3.59
		26		P>A	-0.15	-3.67	3.40

Behind the Vertical Angle Categories	Bridle Type	135	S=65 D=70	S>D	-0.0221	-1.2631	1.1960
	<-10 degrees	135			-0.0065	-0.0205	0.0074
	-10-0 degrees	135			0.0048	-0.0102	0.0201
	Bridle Type * <-10 degrees	135	S=65 D=70	S>D	0.0010	-0.0206	0.0203
	Bridle Type * -10-0 degrees	135	S=65 D=70	S>D	-0.0060	-0.0265	0.0147

Table S13. Component 4 (Full Body Conflict Behaviours 2) Bayesian Regression Model Results. Predictive/trend results are show in bold. (S=Snaffle Bridle; D=Double Bridle; E=Elementary, M=Medium, A=Advanced Medium, P=Prix St. George, I=Intermediate I; R=Regionals, T=Typical; Mr=Male, Fr=Female; EB=Ear Bonnet, nEB=No Ear Bonnet; Lr=Loose ring, Eb=Eggbutt, Hc=Hanging cheek, Dr=D-ring; PF=Professionally Fitted, nPF=Not professionally fitted, MT=Regular maintenance treatments, nMT=No maintenance treatments; nDB=Does not wear double bridle, LH=Less than half of training rides, MH=More than half of training rides; nDB=Does not wear double bridle, 3m=<3 months, 1y=3 month to 1 year, 2y=1-2 years, 5y=>5 years; EM=Elementary or medium, A=Advanced Medium, P= Prix St. George, I2=Intermediate II, G=Grand Prix; E=Elementary, M=Medium, A=Advanced Medium, P=Prix St. George, G=Grand Prix; 6m=>6 months, 1y=6 months to 1 year, 2y=1-2 years, 5y=2-5 years; 1m=>1 month, 3m=1-3 months ago, 6m=3-6 months ago, 12m=6-12 months ago; DJS=Double jointed snaffle, ADJS=Anatomical double jointed snaffle, ES=Eggbutt snaffle, SW=Straightbar Weymouth double bridle, PW=Ported/Myler Weymouth double bridle; 6m=Every 6 months, 7.5m=Every 7.5 months, 12m=Every 12 months; *denotes category interaction).

Model	Variable Name	Sample Size	Category Size	Hypothesis	Estimate	Lower CrI	Upper CrI
Main Data Set	Bridle Type	118	S=60 D=58	S>D	1.32	-0.66	3.32
	Average Nose Angle	118			-0.03	-0.10	0.03
	Competition Level	118	E=50 M=24 A=8	E>A	-0.19	-1.33	0.95
		118	P=21 I=15	I1>A	-0.24	-1.33	0.84
		118		M>A	-0.21	-1.37	0.96
		118		P>A	-0.15	-1.21	0.90
		118		E>I1	0.05	-0.61	0.71
		118		E>M	0.02	-0.79	0.84
		118		E>P	-0.04	-0.65	0.57
		118		I1>M	-0.03	-0.78	0.73
		118		I1>P	-0.09	-0.63	0.45
		118		M>P	-0.06	-0.79	0.67
	Competition Type	118	T=11 R=107	R>T	-0.49	-1.18	0.83
	Rider Gender	118	Fr=97 Mr=21	Mr>Fr		-0.53	0.49
	Ear Bonnet	118	nEB=63 EB=55	F>nF		-0.63	0.10
	Snaffle Cheek Piece	118	Dr=1 Eb=18 Hc=1	Eb>Dr		-2.07	1.68
		118	Lr=98	Hc>Dr		-3.97	1.40
		118		Lr>Dr		-1.87	1.86
		118		Eb>Hc		-0.65	2.83
		118		Eb>Lr		-0.62	0.22
		118		Hc>Lr		-2.99	0.40
	**Patting Score**	118			**0.01**	**-0.00**	**0.03**
	Test Score	118			-0.03	-0.10	0.05
	**Bridle Type * Average Nose Angle**	118	S=60 D=58	**S>D**	**0.14**	**0.05**	**0.24**
	**Bridle Type * Competition Level**	118	S*E=39 D*E=11	S>D*E>A	0.93	-0.56	2.38
		118	S*M=15 D*M=9	S>D*I1>A	-1.12	-3.53	1.27
		118	S*A=4 D*A=4	S>D*M>A	0.02	-1.57	1.58
		118	S*P=1 D*P=20	S>D*P>A	0.76	-1.76	3.27
		118	S*I=1 D*I=14	**S>D*E>I1**	**2.05**	**0.22**	**3.87**
		118		**S>D*E>M**	**0.91**	**0.00**	**1.82**
		118		S>D*E>P	0.17	-1.68	2.00
		118		S>D*I>M	-1.14	-3.03	0.76
		118		S>D*I1>P	-1.88	-4.49	0.77
		118		S>D*M>P	-0.74	-2.63	1.17

	Bridle Type * Competition Type	118	S*T=8 D*T=3 S*R=52 D*R=55 S*T=8 D*T=3 S*R=52 D*R=55	S>D*R>T	-1.07	-2.58	0.41
Questionnaire Bridle Professional Fit	Bridle Type	28	S=17 D=11	S>D	0.45	0.32	1.21
	Bridle Professional Fit	28	PF=8 nPF=20	PF>nPF	-0.64	-1.80	0.51
	Average Nose Angle	28			0.09	0.01	0.17
	Patting Score	28			0.01	-0.03	0.04
	Bridle Type * Bridle Professional Fit	28	S*PF=5 S*nPF=12 D*PF=3 D*nPF=8	S>D*PF>nPF	-0.28	-1.72	1.14
Questionnaire Rider Maintenance Treatment	Bridle Type	28	S=17 D=11	S>D	0.52	-0.27	1.32
	Rider Maintenance Treatment	28	MT=20 nMT=8	MT>nMT	0.36	-0.53	1.27
	Average Nose Angle	28			0.09	0.00	0.18
	Patting Score	28			-0.01	-0.04	0.02
Questionnaire Time Training in Double Bridle	Training in Double Bridle	28	nDB=17 LH=5 MH=6	LH>nDB	-0.44	-1.40	0.52
		28		MH>nDB	-0.41	-1.31	0.48
		28		LH>MH	-0.03	-0.99	0.92
Questionnaire Time Wearing Double Bridle	Time Wearing Double Bridle	28	nDB=17 3m=2 1y=1 2y=6 5y=2	3m>nDB	-0.49	-1.96	0.98
		28		1y>nDB	-0.31	-2.38	1.73
		28		2y>nDB	-0.34	-1.29	0.60
		28		5y>nDB	-0.67	-2.14	0.80
		28		3m>1y	-0.18	-2.18	1.89
		28		3m>2y	-0.15	-1.48	1.18
		28		3m>5y	0.18	-1.48	1.82
		28		1y>2y	0.03	-1.76	1.81
		28		1y>5y	0.36	-1.64	2.36
		28		2y>5y	0.33	-0.98	1.67
Questionnaire Rider Highest Level Trained	Bridle Type	28	S=17 D=11	S>D	0.18	-0.82	1.17
	**Rider Highest Level Trained**	28	EM=4 A=4 P=6 I2=5 G=9	EM>A	0.74	-0.75	2.22
		28		G>A	0.36	-0.96	1.69
		28		I2>A	0.16	-1.09	1.43
		28		**I2>P**	**-0.86**	**-1.78**	**0.07**
		28		P>A	1.01	-0.27	2.29
		28		EM>G	-0.27	-1.28	0.72
		28		EM>I2	0.38	-0.69	1.46
		28		EM>P	-0.27	-1.28	0.72
		28		G>I2	0.20	-0.79	1.18
		28		G>P	-0.65	-1.72	0.41
	Average Nose Angle	28			0.06	-0.04	0.16
	Patting Score	28			-0.02	-0.06	0.02
Questionnaire Horse Highest Level Trained	Bridle Type	28	S=17 D=11	S>D	0.07	-0.83	0.97
	Horse Highest Level Trained	28	E=4 M=12 A=4 P=4 G=4	E>A	0.68	-1.04	2.37
		28		G>A	-0.24	-1.65	1.16
		28		M>A	0.50	-0.60	1.60
		28		P>A	0.11	-1.22	1.44
		28		E>G	0.92	-0.64	2.50
		28		E>M	0.18	-0.92	1.27
		28		E>P	0.57	-0.80	1.94
		28		G>M	-0.74	-1.79	0.30
		28		G>P	-0.35	-1.58	0.85
		28		M>P	0.39	-0.51	1.29
	Average Nose Angle	28			0.05	-0.06	0.17
	Patting Score	28			-0.02	-0.06	0.02
Questionnaire Time Horse Trained Before Starting to Compete	Bridle Type	28	S=17 D=11	S>D	0.70	-0.18	1.58
	Training Before Competing	28	6m=6 1y=6 2y=12 5y=4	1y>6m	-0.23	-1.27	0.83
		28		2y>6m	-0.55	-1.58	0.49
		28		5y>6m	0.31	-0.99	1.63
		28		1y>2y	0.32	-0.50	1.15

		28		1y>5y	-0.54	-1.58	0.49
		28		2y>5y	-0.87	-2.02	0.27
	Average Nose Angle	28			0.06	-0.04	0.16
	Patting Score	28			-0.02	-0.06	0.02
Questionnaire Time Since Last Dental Examination	Bridle Type	28	S=17 D=11	S>D	0.59	-1.12	2.31
	Last Dental Examination	28	1m=4 3m=12 6m=5 12m=7	3m>1m	0.30	-1.29	1.88
		28		6m>1m	0.66	-1.19	2.51
		28		12m>1m	0.31	-1.57	2.21
		28		3m>6m	-0.36	-1.41	0.69
		28		3m>12m	-0.01	-1.10	1.09
		28		6m>12m	0.35	-0.89	1.58
	**Bridle Type*Last Dental**	28	S*1m=3 D*1m=1 S*3m=6 D*3m=6 S*6m=3 D*6m=2 S*12m=5 D*12m=2	S>D*3m>1m	-0.45	-2.39	1.48
		28		S>D*6m>1m	0.86	-1.32	3.01
		28		S>D*12m>1m	-0.72	-2.82	1.38
		28		**S>D*3m>6m**	**-1.32**	**-2.67**	**0.06**
		28		S>D*3m>12m	0.27	-1.08	1.63
		28		**S>D*6m>12m**	**1.59**	**0.04**	**3.14**
	Average Nose Angle	28			0.10	0.01	0.18
	Patting Score	28			-0.00	-0.03	0.03
Questionnaire Dental Examination Frequency	Bridle Type	28	S=17 D=11	S>D	0.42	-0.37	1.20
	Dental Examination Frequency	28	6m=15 7.5m=1 12m=11	6m>12m	0.18	-0.72	1.07
		28		7.5m>12m	0.24	1.80	2.28
		28		6m>7.5m	-0.06	-1.61	1.49
	Average Nose Angle	28			0.09	-0.00	0.18
	Patting Score	28			-0.01	-0.05	0.03
Questionnaire Bit Category	**Bit Category**	26	DJS=4 ADJS=7 ES=4 PW=4 SW=7	**DJS>ADJS**	**-0.74**	**-1.42**	**-0.05**
		26		ES>ADJS	-0.05	-0.85	0.74
		26		PW>ADJS	-0.50	-1.31	0.34
		26		SW>ADJS	-0.38	-1.08	0.33
		26		**DJS>ES**	**-0.69**	**-1.26**	**-0.11**
		26		DJS>PW	-0.24	-0.84	0.36
		26		DJS>SW	-0.36	-0.83	0.11
		26		**ES>PW**	0.45	-0.25	1.15
		26		ES>SW	0.33	-0.27	0.93
		26		PW>SW	-0.12	-0.67	0.43
	Competition Type	26	T=8 R=18	R>T	-1.27	-1.86	-0.68
	Competition Level	26	E=14 M=7 A=1 P=2 I=2	E>A	1.28	-0.01	2.57
		26		I1>A	0.85	-0.70	2.42
		26		M>A	0.47	-0.78	1.70
		26		P>A	0.70	-0.89	2.25
Behind the Vertical Angle Categories	Bridle Type	121	S=60 D=61	S>D	1.1420	-0.1891	2.4696
	<-10 degrees	121			0.0062	-0.0083	0.0208
	-10-0 degrees	121			0.0028	-0.0136	0.0190
	Ear Bonnet	121	EB=57 nEB=64	EB>nEB	-0.2610	-0.6179	0.1053
	Competition Level	121	E=50 M=25 A=8 P=23 I=15	E>A	0.4450	-0.3125	1.2034
		121		E>I1	-0.1081	-1.0105	0.8101
		121		E>M	-0.1453	-0.9364	0.6538
		121		E>P	-0.0036	-0.8523	0.8519
	Test Score	121			-0.0395	-0.1106	0.0270
	**Bridle Type * <-10 degrees**	121	S=60 D=61	**S>D**	**-0.0279**	**-0.0502**	**-0.0057**
	Bridle Type * -10-0 degrees	121	S=60 D=61	S>D	-0.0083	-0.0309	0.0139

Table S14. Component 5 (Full Body Conflict Behaviours 3) Bayesian Regression Model Results. Predictive/trend results are show in bold. (S=Snaffle Bridle; D=Double Bridle; E=Elementary, M=Medium, A=Advanced Medium, P=Prix St. George, I=Intermediate I; R=Regionals, T=Typical; Mr=Male, Fr=Female; EB=Ear Bonnet, nEB=No Ear Bonnet; Lr=Loose ring, Eb=Eggbutt, Hc=Hanging cheek, Dr=D-ring; PF=Professionally Fitted, nPF=Not professionally fitted, MT=Regular maintenance treatments, nMT=No maintenance treatments; nDB=Does not wear double bridle, LH=Less than half of training rides, MH=More than half of training rides; nDB=Does not

wear double bridle, 3m=<3 months, 1y=3 month to 1 year, 2y=1-2 years, 5y=>5 years; EM=Elementary or medium, A=Advanced Medium, P= Prix St. George, I2=Intermediate II, G=Grand Prix; E=Elementary, M=Medium, A=Advanced Medium, P=Prix St. George, G=Grand Prix; 6m=>6 months, 1y=6 months to 1 year, 2y=1-2 years, 5y=2-5 years; 1m=>1 month, 3m=1-3 months ago, 6m=3-6 months ago, 12m=6-12 months ago; DJS=Double jointed snaffle, ADJS=Anatomical double jointed snaffle, ES=Eggbutt snaffle, SW=Straightbar Weymouth double bridle, PW=Ported/Myler Weymouth double bridle; 6m=Every 6 months, 7.5m=Every 7.5 months, 12m=Every 12 months; *denotes category interaction).

Model	Variable Name	Sample Size	Category Size	Hypothesis	Estimate	Lower CrI	Upper CrI
Main Data Set	Bridle Type	118	S=60 D=58	S>D	-0.18	-2.19	1.85
	Average Nose Angle	118			-0.05	-0.11	0.02
	**Competition Level**	118	E=50 M=24 A=8 P=21 I=15	**E>A**	**-1.64**	**-2.83**	**-0.45**
		118		**I1>A**	**-1.39**	**-2.53**	**-0.28**
		118		**M>A**	**-1.72**	**-2.95**	**-0.53**
		118		**P>A**	**-1.22**	**-2.31**	**-0.13**
		118		E>I1	-0.25	-0.90	0.41
		118		E>M	0.08	-0.75	0.90
		118		E>P	-0.42	-1.04	0.20
		118		I1>M	0.33	-0.43	1.09
		118		I1>P	-0.17	-0.71	0.38
		118		M>P	-0.50	-1.23	0.23
	Competition Type	118	T=11 R=107	R>T	0.93	-0.41	2.30
	Rider Gender	118	Fr=97 Mr=21	Mr>Fr	0.10	-0.42	0.62
	Ear Bonnet	118	nEB=63 EB=55	F>nF	-0.11	-0.48	0.27
	Snaffle Cheek Piece - Eggbut	118	Dr=1 Eb=18 Hc=1 Lr=98	Eb>Dr	-0.40	-2.32	1.50
		118		Hc>Dr	-1.30	-4.04	1.42
		118		Lr>Dr	-0.46	-2.35	1.43
		118		Eb>Hc	0.90	-0.88	2.67
		118		Eb>Lr	0.06	-0.37	0.48
		118		Hc>Lr	-0.84	-2.57	0.92
	Patting Score	118			-0.00	-0.02	0.01
	Test Score	118			-0.01	-0.18	0.07
	Bridle Type * Average Nose Angle	118	S=60 D=58	S>D	0.06	-0.04	0.16
	**Bridle Type * Competition Level**	118	S*E=39 D*E=11 S*M=15 D*M=9 S*A=4 D*A=4 S*P=1 D*P=20 S*I=1 D*I=14	**S>D*E>A**	**1.36**	**-0.17**	**2.89**
		118		S>D*I1>A	1.94	-0.53	4.43
		118		**S>D*M>A**	**1.51**	**-0.10**	**3.10**
		118		**S>D*P>A**	**2.75**	**-0.20**	**5.27**
		118		S>D*E>I1	-0.59	-2.42	1.24
		118		S>D*E>M	-0.15	-1.09	0.78
		118		S>D*E>P	-1.40	-3.24	0.46
		118		S>D*I1>M	0.43	-1.47	2.37
		118		S>D*I1>P	-0.81	-3.44	1.84
		118		S>D*M>P	-1.24	-3.17	0.68
	**Bridle Type * Competition Type**	118	S*T=8 D*T=3 S*R=52 D*R=55	**S>D*R>T**	**-1.32**	**-2.87**	**0.23**
Questionnaire Bridle Professional Fit	Bridle Type	28	S=17 D=11	S>D	0.20	-0.50	0.91
	Bridle Professional Fit	28	PF=8 nPF=20	PF>nPF	-0.23	-1.28	0.82
	Competition Level	28		E>A	-0.32	-1.92	1.25
		28		I1>A	1.08	-0.96	3.11
		28		M>A	-0.58	-2.12	0.97
		28		P>A	0.48	-1.56	2.48
	Competition Type	28		R>T	0.06	-0.74	0.82
	Bridle Type * Bridle Professional Fit	28		S>D*PF>nPF	-0.31	-1.62	1.02
	Bridle Type	28	S=17 D=11	S>D	0.00	-0.73	0.73

Rider Maintenance Treatment	Rider Maintenance Treatment	28	MT=20 nMT=8	MT>nMT	-0.16	-0.90	0.59
	Competition level	28	E=15 M=8 A=1 P=2 I=2	E>A	-0.22	-1.83	1.40
		28		I1>A	1.16	-0.82	3.19
		28		M>A	-0.56	-2.11	1.01
		28		P>A	0.56	-1.43	2.55
	Competition Type	28	T=10 R=18	R>T	-0.18	-0.90	0.52
Questionnaire Time Training in Double Bridle	Training in Double Bridle	28	nDB=17 LH=5 MH=6	LH>nDB	0.50	-0.26	1.26
		28		MH>nDB	0.11	-0.61	0.82
		28		LH>MH	0.39	-0.36	1.14
Questionnaire Time Wearing Double Bridle	**Time Wearing Double Bridle**	28	nDB=17 3m=2 1y=1 2y=6 5y=2	3m>nDB	0.23	-0.84	1.30
		29		**3m>5y**	**-1.07**	**-2.26**	**0.13**
		28		1y>nDB	-0.12	-1.59	1.35
		29		**1y>5y**	**-1.43**	**-2.90**	**0.04**
		28		2y>nDB	0.03	-0.65	0.71
		28		**5y>nDB**	**1.30**	**0.23**	**2.40**
		28		3m>1y	0.36	-1.11	1.81
		28		3m>2y	0.20	-0.77	1.16
		28		1y>2y	-0.16	-1.44	1.12
		28		**1y>5y**	**-1.27**	**-2.24**	**-0.30**
Questionnaire Rider Highest Level Trained	Bridle Type	28	S=17 D=11	S>D	0.03	-0.89	0.93
	Rider Highest Level Trained	28	EM=4 A=4 P=6 I2=5 G=9	EM>A	0.06	-1.20	1.32
		28		G>A	-0.11	-1.33	1.11
		28		I2>A	-0.05	-1.28	1.22
		28		P>A	-0.03	-1.09	1.02
		28		EM>G	0.17	-0.82	1.15
		28		EM>I2	0.11	-0.87	1.07
		28		EM>P	0.09	-0.80	0.99
		28		G>I2	-0.06	-1.03	0.91
		28		G>P	-0.08	-1.06	0.90
		28		I2>P	-0.02	-0.94	0.91
	Competition Level	28	E=15 M=8 A=1 P=2 I=2	E>A	-0.37	-2.40	1.67
		28		I1>A	1.07	-1.49	3.65
		28		M>A	-0.50	-2.64	1.29
		28		P>A	0.47	-2.02	2.97
	Competition Type	28	T=10 R=18	R>T	-0.17	-1.03	0.69
Questionnaire Horse Highest Level Trained	Bridle Type	28	S=17 D=11	S>D	0.05	-0.60	0.70
	**Horse Highest Level Trained**	28	E=4 M=12 A=4 P=4 G=4	E>A	0.32	-0.72	1.34
		29		**E>G**	**-0.99**	**-1.96**	**-0.02**
		28		**G>A**	**1.31**	**0.28**	**2.35**
		29		**G>M**	**1.15**	**0.39**	**1.91**
		28		M>A	0.16	-0.65	0.97
		28		P>A	0.52	-0.46	1.52
		28		E>M	0.16	-0.52	0.84
		28		E>P	-0.20	-1.01	0.61
		28		**G>P**	**0.79**	**-0.12**	**1.69**
		28		M>P	-0.36	-1.02	0.31
Questionnaire Time Horse Trained Before Starting to Compete	Bridle Type	28	S=17 D=11	S>D	-0.22	-1.28	0.82
	Training Before Competing	28	6m=6 1y=6 2y=12 5y=4	1y>6m	-0.34	-1.29	0.62
		28		2y>6m	-0.01	-0.89	0.87
		28		5y>6m	-0.51	-1.67	0.66
		28		1y>2y	-0.33	-1.09	0.42
		28		1y>5y	0.17	-0.92	1.23
		28		2y>5y	0.5	-0.63	1.61
	Competition Level	28	E=15 M=8 A=1 P=2 I=2	E>A	-0.57	-2.30	1.16
		28		I1>A	0.66	-1.59	2.89
		28		M>A	-0.95	-2.73	0.83
		28		P>A	0.04	-2.23	2.29
	Competition Type	28	T=10 R=18	R>T	-0.23	-1.00	0.52
Questionnaire Time Since Last Dental Examination	Bridle Type	28	S=17 D=11	S>D	-0.45	-2.33	1.42
	Last Dental Examination	28	1m=4 3m=12 6m=5 12m=7	3m>1m	-0.47	-2.33	1.41
		28		6m>1m	-0.43	-2.37	1.70

		28		12m>1m	0.11	-2.31	2.61
		28		3m>6m	-0.13	-1.28	1.03
		28		3m>12m	-0.58	-1.91	0.76
		28		6m>12m	-0.45	-2.22	1.33
	Bridle Type*Last Dental	28	S*1m=3 D*1m=1 S*3m=6 D*3m=6 S*6m=3 D*6m=2 S*12m=5 D*12m=2	S>D*3m>1m	0.64	-1.52	2.76
		28		S>D*6m>1m	0.73	-1.77	3.19
		28		S>D*12m>1m	-0.02	-2.84	2.73
		28		S>D*3m>6m	-0.09	-1.7	1.48
		28		S>D*3m>12m	0.66	-0.99	2.3
		28		S>D*6m>12m	0.75	-1.47	2.96
	Competition Level	28	E=15 M=8 A=1 P=2 I=2	E>A	-0.50	-2.70	1.76
		28		I>A	0.68	-2.23	3.60
		28		M>A	-0.73	-2.78	1.33
		28		P>A	0.08	-2.83	2.98
	Competition Type	28	T=10 R=18	R>T	-0.04	-1.17	1.09
Questionnaire Dental Examination Frequency	Bridle Type	28	S=17 D=11	S>D	-0.01	-0.78	0.78
	Dental Examination Frequency	28	6m=15 7.5m=1 12m=11	6m>12m	-0.15	-0.81	0.50
		28		7.5m>12m	-0.19	-1.91	1.55
		28		6m>7.5m	0.04	-1.3	1.36
	Competition Level	28	E=15 M=8 A=1 P=2 I=2	E>A	-0.26	-1.89	1.39
		28		I1>A	1.06	-1.01	3.15
		28		M>A	-0.54	-2.17	1.08
		28		P>A	0.45	-1.63	2.49
	Competition Type	28	T=10 R=18	R>T	-0.16	-0.91	0.58
Questionnaire Bit Category	**Bit Category**	26	DJS=4 ADJS=7 ES=4 PW=4 SW=7	DJS>ADJS	0.15	-0.72	1.02
		26		ES>ADJS	-0.53	-1.54	0.48
		26		PW>ADJS	0.34	-0.72	1.36
		26		SW>ADJS	-0.11	-0.99	0.78
		26		**DJS>ES**	**0.68**	**-0.05**	**1.42**
		26		DJS>PW	-0.18	-0.94	0.58
		26		DJS>SW	0.26	-0.35	0.86
		26		**ES>PW**	**-0.87**	**-1.75**	**0.03**
		26		ES>SW	-0.43	-1.19	0.34
		26		PW>SW	0.44	-0.25	1.13
	Competition Type	26	T=8 R=18	R>T	0.16	-0.58	0.90
	Competition Level	26	E=14 M=7 A=1 P=2 I=2	E>A	-1.22	-2.82	0.41
		26		I>A	-0.01	-1.97	1.99
		26		M>A	-1.37	-2.89	0.20
		26		P>A	-0.61	-2.60	1.41
Behind the Vertical Angle Categories	Bridle Type	135	S=65 D=70	S>D	0.0337	-1.1082	1.1726
	**<-10 degrees**	135			**0.0106**	**-0.0025**	**0.0238**
	-10-0 degrees	135			-0.0010	-0.0148	0.0132
	Competition Level	135	E=53 M=27 A=9 P=27 I=19	E>A	-1.0123	-1.6794	-0.3451
		135		E>I1	-0.3327	-1.1221	0.4422
		135		E>M	-1.0185	-1.7252	-0.3107
		135		E>P	-0.4960	-1.2385	0.2457
	Competition Type	135	T=13 R=122	R>T	0.0353	-0.5344	0.6016
	Bridle Type * <-10 degrees	135	S=65 D=70	S>D	-0.0095	-0.0299	0.0109
	Bridle Type * -10-0 degrees	135	S=65 D=70	S>D	-0.0012	-0.0204	0.0181

Table S15. Component 6 (Training-Related Conflict Behaviours) Bayesian Regression Model Results. Predictive/trend results are show in bold. (S=Snaffle Bridle; D=Double Bridle; E=Elementary, M=Medium, A=Advanced Medium, P=Prix St. George, I=Intermediate I; R=Regionals, T=Typical; Mr=Male, Fr=Female; EB=Ear Bonnet, nEB=No Ear Bonnet; Lr=Loose ring, Eb=Eggbutt, Hc=Hanging cheek, Dr=D-ring; PF=Professionally Fitted, nPF=Not professionally fitted, MT=Regular maintenance treatments, nMT=No maintenance treatments; nDB=Does not wear double bridle, LH=Less than half of training rides, MH=More than half of training rides; nDB=Does not wear double bridle, 3m=<3 months, 1y=3 month to 1 year, 2y=1-2

years, 5y=>5 years; EM=Elementary or medium, A=Advanced Medium, P= Prix St. George, I2=Intermediate II, G=Grand Prix; E=Elementary, M=Medium, A=Advanced Medium, P=Prix St. George, G=Grand Prix; 6m=>6 months, 1y=6 months to 1 year, 2y=1-2 years, 5y=2-5 years; 1m=>1 month, 3m=1-3 months ago, 6m=3-6 months ago, 12m=6-12 months ago; DJS=Double jointed snaffle, ADJS=Anatomical double jointed snaffle, ES=Eggbutt snaffle, SW=Straightbar Weymouth double bridle, PW=Ported/Myler Weymouth double bridle; 6m=Every 6 months, 7.5m=Every 7.5 months, 12m=Every 12 months; *denotes category interaction).

Model	Variable Name	Sample Size	Category Size	Hypothesis	Estimate	Lower CrI	Upper CrI
Main Data Set	**Bridle Type**	118	S=60 D=58	**S>D**	**-2.52**	**-4.64**	**-0.38**
	Average Nose Angle	118			0.05	-0.02	0.12
	**Competition Level**	118	E=50 M=24 A=8 P=21 I=15	E>A	0.30	-0.92	1.54
		118		I1>A	0.70	-0.46	1.87
		118		M>A	-0.30	-1.55	0.98
		118		P>A	-0.03	-1.16	1.10
		118		E>I1	-0.40	-1.11	0.29
		118		E>M	0.59	-0.27	1.46
		118		E>P	0.33	-0.32	0.97
		118		**I1>M**	**1.00**	**0.19**	**1.79**
		118		**I1>P**	**0.73**	**0.16**	**1.29**
		118		M>P	-0.27	-1.04	0.50
	**Competition Type**	118	T=11 R=107	**R>T**	**-2.86**	**-4.29**	**-1.43**
	**Rider Gender**	118	Fr=97 Mr=21	**Mr>Fr**	**0.47**	**-0.08**	**1.01**
	Ear Bonnet	118	nEB=63 EB=55	F>nF	-0.11	-0.50	0.29
	**Snaffle Cheek Piece**	118	Dr=1 Eb=18 Hc=1 Lr=98	Eb>Dr	0.11	-1.87	2.09
		118		Hc>Dr	**2.43**	**-0.40**	**5.23**
		118		Lr>Dr	0.18	-1.79	2.15
		118		Eb>Hc	-2.32	-4.15	-0.50
		118		Eb>Lr	-0.07	-0.52	0.38
		118		Hc>Lr	**2.25**	**0.46**	**4.05**
	Patting Score	118			0.00	-0.01	0.02
	Test Score	118			-0.03	-0.11	0.05
	Bridle Type * Average Nose Angle	118	S=60 D=58	S>D	-0.07	-0.17	0.03
	**Bridle Type * Competition Level**	118	S*E=39 D*E=11 S*M=15 D*M=9 S*A=4 D*A=4 S*P=1 D*P=20 S*I=1 D*I=14	S>D*E>A	-0.82	-2.42	0.74
		118		**S>D*I1>A**	**-2.25**	**-4.81**	**0.31**
		118		S>D*M>A	-0.32	-2.00	1.32
		118		S>D*P>A	-1.12	-3.75	1.55
		118		S>D*E>I1	1.43	-0.50	3.36
		118		S>D*E>M	-0.50	-1.47	0.48
		118		S>D*E>P	0.30	-1.65	2.23
		118		**S>D*I1>P**	**0.72**	**0.16**	**1.29**
		118		**S>D*I1>M**	**-1.93**	**-3.92**	**0.06**
		118		S>D*M>P	0.80	-1.21	2.80
	**Bridle Type * Competition Type**	118	S*T=8 D*T=3 S*R=52 D*R=55	**S>D*R>T**	**3.07**	**1.43**	**4.68**
Bridle fit model	Bridle Type	28	S=17 D=11	S>D	1.31	-2.45	-0.19
	Bridle Professional Fit	28	PF=8 nPF=20	PF>nPF	0.26	-1.28	1.78
	Competition Type	28	T=10 R=18	R>T	-1.00	-2.18	0.20
	Competition Level	28	E=15 M=8 A=1 P=2 I=2	E>A	-2.38	-5.03	0.25
		28		I1>A	-2.10	-5.34	1.12
		28		M>A	-2.41	-4.91	0.08
		28		P>A	-3.61	-6.85	-0.40
	Snaffle Cheekpiece	28	Eb=4 Lr=24 Hc=0 Dr=0		0.37	-1.04	1.80
	Bridle Type * Bridle Professional Fit	28	S*PF=5 S*nPF=12 D*PF=3 D*nPF=8	S>D*PF>nPF	-0.33	-2.28	1.66
	Bridle Type	28	S=17 D=11	S>D	-1.46	-2.50	-0.43

Questionnaire Rider Maintenance Treatment	Rider Maintenance Treatment	28	MT=20 nMT=8	MT>nMT	-0.20	-1.26	0.85
	Competition Type	28	T=10 R=18	R>T	-1.02	-2.05	-0.02
	Competition Level	28	E=15 M=8 A=1 P=2 I=2	E>A	-2.28	-4.85	0.24
		28		I1>A	-1.90	-4.98	1.09
		28		M>A	-2.37	-4.80	0.01
		28		P>A	-3.43	-6.48	-0.40
	Snaffle Cheekpiece	28	Eb=4 Lr=24 Hc=0 Dr=0	Lr>Eb	0.40	-0.90	1.71
Questionnaire Time Training in Double Bridle	**Training in Double Bridle**	28	nDB=17 LH=5 MH=6	LH>nDB	0.75	-0.47	1.96
		28		MH>nDB	**1.29**	**0.15**	**2.41**
		28		LH>MH	-0.54	-1.74	0.66
Questionnaire Time Wearing Double Bridle	**Time Wearing Double Bridle**	28	nDB=17 3m=2 1y=1 2y=6 5y=2	3m>nDB	0.29	-1.46	2.05
		28		3m>2y	**-1.29**	**-2.88**	**0.30**
		28		1y>nDB	1.07	-1.43	3.54
		28		**2y>nDB**	**1.58**	**0.46**	**2.69**
		28		5y>nDB	0.18	-1.57	1.93
		28		**2y>5y**	**1.40**	**-0.18**	**2.99**
		28		3m>1y	-0.78	-3.19	1.64
		28		3m>5y	0.11	-1.82	2.04
		28		1y>2y	-0.51	-2.67	1.63
		28		1y>5y	0.89	-1.52	3.29
Questionnaire Rider Highest Level Trained	Bridle Type	28	S=17 D=11	S>D	-0.98	-2.11	0.16
	**Rider Highest Level Trained**	28	EM=4 A=4 P=6 I2=5 G=9	EM>A	1.02	-0.58	2.62
		28		**G>A**	**1.30**	**-0.24**	**2.85**
		28		I2>A	0.64	-0.92	2.26
		28		P>A	0.56	-1.04	2.17
		28		EM>G	-0.29	-1.56	0.95
		28		EM>I2	0.38	-0.84	1.61
		28		EM>P	0.46	-0.89	1.79
		28		G>I2	0.66	-0.59	1.91
		28		G>P	0.75	-0.70	2.20
		28		I2>P	0.08	-1.32	1.50
	Competition Type	28	T=10 R=18	R>T	-1.12	-2.27	0.02
	Competition Level	28	E=15 M=8 A=1 P=2 I=2	E>A	-2.29	-5.49	0.94
		28		I1>A	-2.09	-5.84	1.64
		28		M>A	-2.09	-5.15	0.94
		28		P>A	-3.60	-7.40	0.17
	Snaffle Cheekpiece	28	Eb=4 Lr=24 Hc=0 Dr=0	Lr>Eb	0.29	-1.38	1.97
Questionnaire Horse Highest Level Trained	Bridle Type	28	S=17 D=11	S>D	-1.51	-2.53	-0.49
	Horse Highest Level Trained	28	E=4 M=12 A=4 P=4 G=4	E>A	0.82	-0.78	2.40
		29		G>A	-0.20	-1.79	1.42
		28		M>A	0.03	-1.22	1.29
		28		P>A	0.27	-1.33	1.89
		28		E>G	1.02	-0.49	2.53
		28		E>M	0.79	-0.26	1.85
		28		E>P	-0.20	-1.01	0.61
		28		G>M	-0.23	-1.44	0.97
		28		G>P	-0.47	-1.99	1.06
		28		M>P	-0.24	-1.29	0.82
	Competition Type	28	T=10 R=18	R>T	-1.24	-2.17	-0.31
Questionnaire Time Horse Trained Before Starting to Compete	Bridle Type	28	S=17 D=11	S>D	-0.43	-1.78	0.91
	**Training Before Competing**	28	6m=6 1y=6 2y=12 5y=4	1y>6m	-0.16	-1.48	1.16
		28		2y>6m	-0.76	-1.92	0.38
		28		5y>6m	0.81	-0.71	2.35
		28		1y>2y	0.60	-0.38	1.57
		28		1y>5y	-0.98	-2.38	0.43
		28		**2y>5y**	**-1.58**	**-3.00**	**-0.15**
	Competition Type	28	T=10 R=18	R>T	-0.86	-1.84	0.11
		28		E>A	-2.16	-4.57	0.27

	Competition Level	28	E=15 M=8 A=1 P=2 I=2	I1>A	-0.82	-3.94	2.32
		28		M>A	-1.93	-4.36	0.55
		28		P>A	-2.33	-5.46	0.81
	Snaffle Cheekpiece	28	Eb=4 Lr=24 Hc=0 Dr=0	Lr>Eb	0.28	-0.99	1.56
Questionnaire Time Since Last Dental Examination	Bridle Type	28	S=17 D=11	S>D	-0.21	-2.63	2.22
	Last Dental Examination	28	1m=4 3m=12 6m=5 12m=7	3m>1m	1.20	-1.24	3.64
		28		6m>1m	0.87	-1.76	3.48
		28		12m>1m	0.47	-2.72	3.69
		28		3m>6m	0.33	-1.16	1.82
		28		3m>12m	0.72	-1.00	2.46
		28		6m>12m	0.40	-1.88	2.67
	Bridle Type*Last Dental	28	S*1m=3 D*1m=1 S*3m=6 D*3m=6 S*6m=3 D*6m=2 S*12m=5 D*12m=2	S>D*3m>1m	-1.62	-4.40	1.21
		28		S>D*6m>1m	-1.64	-4.89	1.59
		28		S>D*12m>1m	0.02	-3.58	3.68
		28		S>D*3m>6m	0.02	-2.09	2.12
		28		S>D*3m>12m	-1.64	-3.81	0.58
		28		S>D*6m>12m	-1.66	-4.53	1.24
	Competition Type	28	T=10 R=18	R>T	-1.50	-2.94	-0.04
	Competition Level	28	E=15 M=8 A=1 P=2 I=2	E>A	-1.54	-4.61	1.49
		28		I1>A	-0.96	-4.81	2.90
		28		M>A	-1.82	-4.61	0.98
		28		P>A	-2.46	-6.32	1.43
	Snaffle Cheekpiece	28	Eb=4 Lr=24 Hc=0 Dr=0	Lr>Eb	0.62	-1.01	2.28
Questionnaire Dental Examination Frequency	Bridle Type	28	S=17 D=11	S>D	-1.4	-2.5	-0.3
	Dental Examination Frequency	28	6m=15 7.5m=1 12m=11	6m>12m	-0.13	-1.06	0.80
		28		7.5m>12m	0.07	-2.40	2.54
		28		6m>7.5m	-0.21	-2.10	1.69
	Competition Type	28	T=10 R=18	R>T	-1.02	-2.08	0.05
	Competition Level	28	E=15 M=8 A=1 P=2 I=2	E>A	-2.35	-4.97	0.24
		28		I1>A	-1.99	-5.15	1.09
		28		M>A	-2.37	-4.92	0.13
		28		P>A	-3.52	-6.63	-0.42
	Snaffle Cheekpiece	28	Eb=4 Lr=24 Hc=0 Dr=0	Lr>Eb	0.38	-0.96	1.74
Questionnaire Bit Category	**Bit Category**	26	DJS=4 ADJS=7 ES=4 PW=4 SW=7	DJS>ADJS	-0.41	-1.82	0.97
		26		ES>ADJS	-0.67	-2.27	0.94
		26		PW>ADJS	0.44	-1.22	2.14
		26		**SW>ADJS**	**1.29**	**-0.13**	**2.71**
		26		DJS>ES	0.25	-0.94	1.43
		26		DJS>PW	-0.86	-2.06	0.34
		26		**DJS>SW**	**-1.70**	**-2.65**	**-0.75**
		26		ES>PW	-1.11	-2.52	0.29
		26		**ES>SW**	**-1.95**	**-3.15**	**-0.76**
		26		PW>SW	-0.84	-1.91	0.23
	Competition Type	26	T=8 R=18	R>T	-1.08	-2.27	0.10
	Competition Level	26	E=14 M=7 A=1 P=2 I=2	E>A	-2.37	-5.02	0.23
		26		I1>A	-1.76	-4.95	1.45
		26		M>A	-2.33	-4.82	0.14
		26		P>A	-3.26	-6.46	-0.15
Behind the Vertical Angle Categories	Bridle Type	135	S=65 D=70	S>D	-0.8838	-2.1172	0.3895
	**<-10 degrees**	135			**-0.0114**	**-0.0255**	**0.0027**
	-10-0 degrees	135			-0.0045	-0.0195	0.0108
	Bridle Type * <-10 degrees	135	S=65 D=70	S>D	0.0140	-0.0082	0.0359
	Bridle Type * -10-0 degrees	135	S=65 D=70	S>D	0.0114	-0.0103	0.0325
	Competition Level	135	E=53 M=27 A=9 P=27 I=19	E>A	-0.5322	-1.2516	0.1894
		135		E>I1	-0.1422	-0.9805	0.6862
		135		E>M	-0.5302	-1.2933	0.2301
		135		E>P	-0.7196	-1.5156	0.0759

	Competition Type	135	T=13 R=122	R>T	-0.5985	-1.2048	0.0169
	Snaffle Cheekpiece	135	Eb=23 Lr=110 Dr=1 Hc=1	Eb>Dr	0.1684	-1.8608	2.1990
		135		Hc>Dr	2.4914	-0.4234	5.3901
		135		Lr>Dr	0.2383	-1.7754	2.2723

Table S16. Component 7 (Spook-Related Conflict Behaviours) Bayesian Regression Model Results. Predictive/trend results are show in bold. (S=Snaffle Bridle; D=Double Bridle; E=Elementary, M=Medium, A=Advanced Medium, P=Prix St. George, I=Intermediate I; R=Regionals, T=Typical; Mr=Male, Fr=Female; EB=Ear Bonnet, nEB=No Ear Bonnet; Lr=Loose ring, Eb=Eggbutt, Hc=Hanging cheek, Dr=D-ring; PF=Professionally Fitted, nPF=Not professionally fitted, MT=Regular maintenance treatments, nMT=No maintenance treatments; nDB=Does not wear double bridle, LH=Less than half of training rides, MH=More than half of training rides; nDB=Does not wear double bridle, 3m=<3 months, 1y=3 month to 1 year, 2y=1-2 years, 5y=>5 years; EM=Elementary or medium, A=Advanced Medium, P= Prix St. George, I2=Intermediate II, G=Grand Prix; E=Elementary, M=Medium, A=Advanced Medium, P=Prix St. George, G=Grand Prix; 6m=>6 months, 1y=6 months to 1 year, 2y=1-2 years, 5y=2-5 years; 1m=>1 month, 3m=1-3 months ago, 6m=3-6 months ago, 12m=6-12 months ago; DJS=Double jointed snaffle, ADJS=Anatomical double jointed snaffle, ES=Eggbutt snaffle, SW=Straightbar Weymouth double bridle, PW=Ported/Myler Weymouth double bridle; 6m=Every 6 months, 7.5m=Every 7.5 months, 12m=Every 12 months; *denotes category interaction).

Model	Variable Name	Sample Size	Category Size	Hypothesis	Estimate	Lower CrI	Upper CrI
Main Data Set	**Bridle Type**	118	S=60 D=58	**S>D**	**2.19**	**-0.01**	**4.37**
	Average Nose Angle	118			0.01	-0.06	0.08
	Competition Level	118	E=50 M=24 A=8 P=21 I=15	E>A	0.58	-0.70	1.84
		118		I1>A	0.56	-0.63	1.76
		118		M>A	0.36	-0.94	1.65
		118		P>A	0.17	-1.00	1.33
		118		E>I1	0.01	-0.70	0.71
		118		E>M	0.22	-0.67	1.10
		118		E>P	0.40	-0.26	1.06
		118		I1>M	0.21	-0.62	1.04
		118		I1>P	0.39	-0.20	0.98
		118		M>P	0.18	-0.61	0.98
	Competition Type	118	T=11 R=107	R>T	0.06	-1.39	1.53
	Rider Gender	118	Fr=97 Mr=21	Mr>Fr	0.36	-0.20	0.92
	Ear Bonnet	118	nEB=63 EB=55	F>nF	-0.02	-0.43	0.38
	Snaffle Cheek Piece	118	Dr=1 Eb=18 Hc=1 Lr=98	Eb>Dr	-0.34	-2.41	1.70
		118		Hc>Dr	0.06	-2.88	3.00
		118		Lr>Dr	-0.13	-2.18	1.88
		118		Eb>Hc	-0.40	-2.29	1.52
		118		Eb>Lr	-0.21	-0.67	0.25
		118		Hc>Lr	0.19	-1.69	2.07
	Patting Score	118			-0.01	-0.03	0.01
	Test Score	118			-0.02	-0.11	0.06
	Bridle Type * Average Nose Angle	118	S=60 D=58	S>D	-0.04	-0.15	0.06
	Bridle Type * Competition Level	118	S*E=39 D*E=11 S*M=15 D*M=9 S*A=4 D*A=4 S*P=1 D*P=20 S*I=1 D*I=14	S>D*E>A	-0.71	-2.35	0.95
		118		S>D*I1>A	-0.95	-3.62	1.75
		118		S>D*I>1M	-0.46	-2.18	1.29
		118		S>D*I1>P	-0.57	-3.34	2.21
		118		S>D*E>I1	0.24	-1.75	2.23
		118		S>D*E>M	-0.26	-1.27	0.76
		118		S>D*E>P	-0.14	-2.19	1.86
		118		S>D*I1>M	-0.50	-2.57	1.59
		118		S>D*I1>P	-0.38	-3.27	2.53
		118		S>D*M>P	0.12	-2.00	2.22

	**Bridle Type * Competition Type**	118	S*T=8 D*T=3 S*R=52 D*R=55	**S>D*R>T**	**-1.95**	**-3.60**	**0.30**
Questionnaire Bridle Professional Fit	Bridle Type	28	S=17 D=11	S>D	0.41	-0.69	1.52
	Bridle Professional Fit	28	PF=8 nPF=20	PF>nPF	0.04	-1.25	1.95
	Competition Type	28	T=10 R=18	R>T	-0.65	-1.74	0.44
	Bridle Type * Bridle Professional Fit	28	S*PF=5 S*nPF=12 D*PF=3 D*nPF=8	S>D*PF>nPF	-0.53	-2.57	1.48
Questionnaire Rider Maintenance Treatment	Bridle Type	28	S=17 D=11	S>D	0.13	-0.90	1.17
	Rider Maintenance Treatment	28	MT=20 nMT=8	MT>nMT	-0.26	-1.39	0.88
	Competition Type	28	T=10 R=18	R>T	-0.65	-1.56	0.28
Questionnaire Time Training in Double Bridle	Training in Double Bridle	28	nDB=17 LH=5 MH=6	LH>nDB	-0.40	-1.60	0.80
		28		MH>nDB	-0.30	-1.43	0.81
		28		LH>MH	-0.1	-1.28	1.09
Questionnaire Time Wearing Double Bridle	Time Wearing Double Bridle	28	nDB=17 3m=2 1y=1 2y=6 5y=2	3m>nDB	-0.32	-2.19	1.53
		28		1y>nDB	-0.28	-2.85	2.29
		28		2y>nDB	-0.29	-1.47	0.88
		28		5y>nDB	-0.55	-2.40	1.30
		28		3m>1y	-0.04	-2.56	2.49
		28		3m>2y	-0.03	-1.71	1.65
		28		3m>5y	0.24	-1.81	2.31
		28		1y>2y	0.01	-2.19	2.23
		28		1y>5y	0.27	-2.25	2.82
		28		2y>5y	0.26	-1.42	1.94
Questionnaire Rider Highest Level Trained	Bridle Type	28	S=17 D=11	S>D	0.38	-0.94	1.68
	Rider Highest Level Trained	28	EM=4 A=4 P=6 I2=5 G=9	EM>A	-1.25	-2.97	0.48
		28		G>A	-0.87	-2.58	0.86
		28		I2>A	-1.10	-2.76	0.57
		28		P>A	-1.13	-2.70	0.43
		28		EM>G	-0.39	-1.75	0.97
		28		EM>I2	-0.16	-1.47	1.13
		28		EM>P	-0.12	-1.42	1.19
		28		G>I2	0.23	-1.09	1.55
		28		G>P	0.27	-1.16	1.68
		28		I2>P	0.04	-1.17	1.27
	Competition Type	28	T=10 R=18	R>T	-0.42	-1.46	0.60
Questionnaire Horse Highest Level Trained	Bridle Type	28	S=17 D=11	S>D	0.33	-0.75	1.42
	**Horse Highest Level Trained**	28	E=4 M=12 A=4 P=4 G=4	E>A	-0.14	-1.81	1.53
		29		**E>P**	**-1.53**	**-2.94**	**-0.15**
		28		G>A	0.37	-1.32	2.10
		28		M>A	0.15	-1.17	1.49
		29		**M>P**	**-1.24**	**-2.36**	**-0.12**
		28		**P>A**	**1.39**	**-0.29**	**3.12**
		28		E>G	-0.51	-2.15	1.09
		28		E>M	-0.29	-1.43	0.84
		28		G>M	0.22	-1.09	1.52
		28		G>P	-1.02	-2.65	0.60
	Competition Type	28	T=10 R=18	R>T	-0.37	-1.37	0.63
Questionnaire Time Horse Trained Before Starting to Compete	Bridle Type	28	S=17 D=11	S>D	0.05	-1.11	1.21
	Training Before Competing	28	6m=6 1y=6 2y=12 5y=4	1y>6m	0.24	-1.16	1.65
		28		2y>6m	0.59	-0.63	1.79
		28		5y>6m	0.04	-1.73	1.81
		28		**1y>2y**	-0.35	-1.4	0.7
		28		1y>5y	0.2	-1.21	1.57
		28		2y>5y	0.54	-0.89	1.96
	Competition Type	28	T=10 R=18	R>T	-0.73	-1.70	0.24
	Bridle Type	28	S=17 D=11	S>D	-0.08	-3.13	2.96

Questionnaire Time Since Last Dental Examination	Last Dental Examination	28	1m=4 3m=12 6m=5 12m=7	3m>1m	-0.20	-3.10	2.67
		28		6m>1m	-0.28	-3.57	3.04
		28		12m>1m	-0.23	-3.46	2.97
		28		3m>6m	0.09	-1.69	1.87
		28		3m>12m	0.04	-1.75	1.86
		28		6m>12m	-0.05	-2.31	2.25
	Bridle Type*Last Dental	28	S*1m=3 D*1m=1 S*3m=6 D*3m=6 S*6m=3 D*6m=2 S*12m=5 D*12m=2	S>D*3m>6m	0.36	-2.02	2.76
		28		S>D*3m>12m	0.46	-1.69	2.7
		28		S>D*6m>12m	0.1	-2.64	2.85
		28		S>D*3m>1m	0.64	-2.76	4.03
		28		S>D*6m>1m	0.27	-3.70	4.31
		28		S>D*12m>1m	0.17	-3.63	3.98
	Competition Type	28	T=10 R=18	R>T	-0.64	1.94	0.65
Questionnaire Dental Examination Frequency	Bridle Type	28	S=17 D=11	S>D	0.42	-0.55	1.41
	Dental Examination Frequency	28	6m=15 7.5m=1 12m=11	6m>12m	0.45	-0.50	1.40
		28		7.5m>12m	0.63	-1.91	3.18
		28		6m>7.5m	-0.18	-2.25	1.88
	Competition Type	28	T=10 R=18	R>T	-0.66	-1.59	0.29
Questionnaire Bit Category	Bit Category	26	DJS=4 ADJS=7 ES=4 PW=4 SW=7	DJS>ADJS	0.91	-0.93	2.69
		26		ES>ADJS	-0.13	-2.26	1.95
		26		PW>ADJS	-0.05	-2.22	2.11
		26		SW>ADJS	0.11	-1.70	1.93
		26		DJS>ES	1.03	-0.51	2.60
		26		DJS>PW	0.95	-0.60	2.52
		26		DJS>SW	0.80	-0.47	2.07
		26		ES>PW	-0.08	-1.89	1.76
		26		ES>SW	-0.23	-1.81	1.35
		26		PW>SW	-0.16	-1.55	1.23
	Competition Type	26	T=8 R=18	R>T	-0.54	-2.07	1.01
	Competition Level	26	E=14 M=7 A=1 P=2 I=2	E>A	-0.47	-3.91	2.89
		26		I1>A	-0.13	-4.22	3.93
		26		M>A	-0.13	-3.38	3.11
		26		P>A	-0.15	-4.32	3.94
Behind the Vertical Angle Categories	Bridle Type	135	S=65 D=70	S>D	-0.5428	-1.6888	0.6139
	<-10 degrees	135			0.0035	-0.0097	0.0168
	-10-0 degrees	135			-0.0012	-0.0153	0.0123
	Bridle Type * <-10 degrees	135	S=65 D=70	S>D	-0.0007	-0.0215	0.0198
	Bridle Type * -10-0 degrees	135	S=65 D=70	S>D	0.0122	-0.0074	0.0316
	Competition Type	135	T=13 R=122	R>T	-1.0853	-1.6543	-0.5182

Table S17. Component 8 (Full Body Conflict Behaviours 4) Bayesian Regression Model Results. Predictive/trend results are show in bold. (S=Snaffle Bridle; D=Double Bridle; E=Elementary, M=Medium, A=Advanced Medium, P=Prix St. George, I=Intermediate I; R=Regionals, T=Typical; Mr=Male, Fr=Female; EB=Ear Bonnet, nEB=No Ear Bonnet; Lr=Loose ring, Eb=Eggbutt, Hc=Hanging cheek, Dr=D-ring; PF=Professionally Fitted, nPF=Not professionally fitted, MT=Regular maintenance treatments, nMT=No maintenance treatments; nDB=Does not wear double bridle, LH=Less than half of training rides, MH=More than half of training rides; nDB=Does not wear double bridle, 3m=<3 months, 1y=3 month to 1 year, 2y=1-2 years, 5y=>5 years; EM=Elementary or medium, A=Advanced Medium, P= Prix St. George, I2=Intermediate II, G=Grand Prix; E=Elementary, M=Medium, A=Advanced Medium, P=Prix St. George, G=Grand Prix; 6m=>6 months, 1y=6 months to 1 year, 2y=1-2 years, 5y=2-5 years; 1m=>1 month, 3m=1-3 months ago, 6m=3-6 months ago, 12m=6-12 months ago; DJS=Double jointed snaffle, ADJS=Anatomical double jointed snaffle, ES=Eggbutt snaffle, SW=Straightbar Weymouth double bridle, PW=Ported/Myler Weymouth double bridle; 6m=Every 6 months, 7.5m=Every 7.5 months, 12m=Every 12 months; *denotes category interaction).

**Model**	**Variable Name**	**Sample Size**	**Category Size**	**Hypothesis**	**Estimate**	**Lower CrI**	**Upper CrI**

Main Data Set	Bridle Type	118	S=60 D=58	S>D	0.10	-2.14	2.33
	Average Nose Angle	118			0.05	-0.02	0.12
	Competition Level	118	E=50 M=24 A=8 P=21 I=15	E>A	0.12	-1.18	1.41
		118		I1>A	0.36	-0.87	1.60
		118		M>A	-0.13	-1.45	1.21
		118		P>A	0.49	-0.71	1.69
		118		E>I1	-0.24	-0.97	0.49
		118		E>M	0.25	-0.66	1.15
		118		E>P	-0.38	-1.06	0.30
		118		I1>M	0.49	-0.36	1.32
		118		I1>P	-0.13	-0.74	0.46
		118		M>P	-0.63	-1.42	0.18
	Competition Type	118	T=11 R=107	R>T	-0.41	-1.87	1.09
	Rider Gender	118	Fr=97 Mr=21	Mr>Fr	0.08	-0.49	0.65
	Ear Bonnet	118	nEB=63 EB=55	F>nF	0.22	-0.19	0.63
	**Snaffle Cheek Piece**	118	Dr=1 Eb=18 Hc=1 Lr=98	Eb>Dr	1.32	-0.79	3.43
		118		Hc>Dr	0.07	-2.90	3.06
		118		Lr>Dr	0.39	-1.71	2.46
		118		Eb>Hc	1.25	-0.68	3.19
		118		**Eb>Lr**	**0.92**	**0.45**	**1.39**
		118		Hc>Lr	-0.32	-2.23	1.58
	Patting Score	118			0.00	-0.01	0.02
	Test Score	118			-0.00	-0.09	0.08
	Bridle Type * Average Nose Angle	118	S=60 D=58	S>D	-0.07	-0.18	0.04
	Bridle Type * Competition Level	118	S*E=39 D*E=11 S*M=15 D*M=9 S*A=4 D*A=4 S*P=1 D*P=20 S*I=1 D*I=14	S>D*E>A	0.30	-1.37	1.97
		118		S>D*I1>A	0.94	-1.77	3.66
		118		S>D*M>A	-0.09	-1.88	1.69
		118		S>D*P>A	0.94	-1.89	3.79
		118		S>D*E>I1	-0.65	-2.69	1.38
		118		S>D*E>M	0.39	-0.62	1.42
		118		S>D*E>P	-0.64	-2.69	1.40
		118		S>D*I>M	1.03	-1.06	3.16
		118		S>D*I>P	0.00	-2.92	2.93
		118		S>D*M>P	-1.03	-3.11	1.08
	Bridle Type * Competition Type	118	S*T=8 D*T=3 S*R=52 D*R=55	S>D*R>T	-0.41	-2.13	1.27
Bridle fitted Professionally	Bridle Type	28	S=17 D=11	S>D	0.39	-0.81	1.57
	Bridle Professional Fit	28	PF=8 nPF=20	PF>nPF	0.03	-1.77	1.81
	Bridle Type * Bridle Professional Fit	28	S*PF=5 S*nPF=12 D*PF=3 D*nPF=8	S>D*PF>nPF	-1.07	-3.37	1.19
Questionnaire Rider Maintenance Treatment	Bridle Type	28	S=17 D=11	S>D	0.27	-0.93	1.45
	Rider Maintenance Treatment	28	MT=20 nMT=8	MT>nMT	0.42	-0.88	1.70
Questionnaire Time Training in Double Bridle	Training in Double Bridle	28	nDB=17 LH=5 MH=6	LH>nDB	-0.02	-1.40	1.35
		28		MH>nDB	-0.11	-1.40	1.17
		28		LH>MH	0.09	-1.28	1.43
Questionnaire Time Wearing Double Bridle	Time Wearing Double Bridle	28	nDB=17 3m=2 1y=1 2y=6 5y=2	3m>nDB	-0.29	-2.37	1.80
		28		1y>nDB	0.04	-2.92	2.97
		28		2y>nDB	-0.14	-1.48	1.20
		28		5y>nDB	0.28	-1.81	2.36
		28		3m>1y	-0.33	-3.22	2.55
		28		3m>2y	-0.15	-2.07	1.73
		28		3m>5y	-0.57	-2.86	1.75
		28		1y>2y	0.18	-2.36	2.70
		28		1y>5y	-0.24	-3.09	2.58
		28		2y>5y	-0.42	-2.29	1.47
	Bridle Type	28	S=17 D=11	S>D	-0.16	-1.63	1.30
		28		EM>A	0.34	-1.57	2.26

Questionnaire Rider Highest Level Trained	**Rider Highest Level Trained**	28	EM=4 A=4 P=6 I2=5 G=9	G>A	0.26	-1.59	2.15
		28		I2>A	-0.29	-2.10	1.48
		29		**I2>P**	**-1.47**	**-2.85**	**-0.08**
		28		P>A	1.18	-0.56	2.94
		28		EM>G	0.08	-1.46	1.62
		28		EM>I2	0.63	-0.86	2.12
		28		EM>P	-0.84	-2.30	0.62
		28		G>I2	0.55	-0.93	2.02
		28		G>P	-0.92	-2.49	0.70
Questionnaire Horse Highest Level Trained	Bridle Type	28	S=17 D=11	S>D	0.21	-1.14	1.55
	Horse Highest Level Trained	28	E=4 M=12 A=4 P=4 G=4	E>A	0.28	-1.85	2.40
		28		G>A	0.73	-1.39	2.28
		28		M>A	0.51	-1.17	2.19
		28		P>A	0.14	-1.90	2.21
		28		E>G	-0.45	-2.44	1.52
		28		E>M	-0.23	-1.63	1.18
		28		E>P	0.13	-1.55	1.82
		28		G>M	0.22	-1.34	1.73
		28		G>P	0.58	-1.29	2.45
		28		M>P	0.37	-1.01	1.76
Questionnaire Time Horse Trained Before Starting to Compete	Bridle Type	28	S=17 D=11	S>D	0.34	-0.89	1.60
	**Training Before Competing**	28	6m=6 1y=6 2y=12 5y=4	1y>6m	0.98	-0.54	2.52
		28		**1y>2y**	**1.21**	**0.08**	**2.35**
		28		2y>6m	-0.23	-1.56	1.11
		28		5y>6m	0.24	-1.65	2.10
		28		1y>5y	0.75	-0.75	2.22
		28		2y>5y	-0.46	-2.01	1.03
Questionnaire Time Since Last Dental Examination	Bridle Type	28	S=17 D=11	S>D	-0.43	-3.34	2.44
	Last Dental Examination	28	1m=4 3m=12 6m=5 12m=7	3m>1m	0.05	-2.67	2.74
		28		6m>1m	0.05	-3.05	3.08
		28		12m>1m	0.47	-2.58	3.50
		28		3m>6m	0.00	-1.69	1.70
		28		3m>12m	-0.43	-2.10	1.23
		28		6m>12m	-0.43	-2.52	1.63
	**Bridle Type*Last Dental**	28	S*1m=3 D*1m=1	**S>D*3m>6m**	**-2.21**	**-4.44**	**0.03**
		28	S*3m=6 D*3m=6	S>D*3m>12m	0.61	-1.46	2.68
		28	S*6m=3 D*6m=2	**S>D*6m>12m**	**2.83**	**0.26**	**5.40**
		28	S*12m=5	S>D*3m>1m	0.31	-2.92	3.55
		28	D*12m=2	S>D*6m>1m	2.52	-1.19	6.17
		28		S>D*12m>1m	-0.30	-3.80	3.27
Questionnaire Dental Examination Frequency	Bridle Type	28	S=17 D=11	S>D	0.21	-0.93	1.36
	Dental Examination Frequency	28	6m=15 7.5m=1 12m=11	6m>12m	0.39	-0.76	1.52
		28		6m>7.5m	-0.07	-2.43	2.31
		28		7.5m>12m	0.46	-2.55	3.45
Questionnaire Bit Category	**Bit Category**	26	DJS=4 ADJS=7 ES=4 PW=4 SW=7	DJS>ADJS	0.71	-0.57	1.97
		26		**ES>ADJS**	**2.40**	**0.95**	**3.88**
		26		PW>ADJS	0.53	-0.97	2.05
		26		SW>ADJS	0.52	-0.77	1.81
		26		**DJS>ES**	**-1.69**	**-2.77**	**-0.61**
		26		DJS>PW	0.18	-0.91	1.26
		26		DJS>SW	0.19	-0.69	1.08
		26		**ES>PW**	**1.87**	**0.59**	**3.15**
		26		**ES>SW**	**1.88**	**0.77**	**2.98**
		26		PW>SW	0.01	-0.97	1.00
	Competition Type	26	T=8 R=18	R>T	-2.40	-3.50	-1.31
	Competition Level	26	E=14 M=7 A=1 P=2 I=2	E>A	4.98	2.59	7.37
		26		I1>A	5.30	2.37	8.18
		26		M>A	2.86	0.57	5.13
		26		P>A	5.72	2.78	8.62
Behind the Vertical Angle Categories	Bridle Type	135	S=65 D=70	S>D	0.1637	-1.0636	1.3817
	<-10 degrees	135			-0.0109	-0.0248	0.0028
	-10-0 degrees	135			-0.0003	-0.0151	0.0147
	**Bridle Type * <-10 degrees**	135	S=65 D=70	**S>D**	**0.0215**	**-0.0001**	**0.0432**

	Bridle Type * -10-0 degrees	135	S=65 D=70	S>D	-0.0101	-0.0308	0.0109
	Snaffle Cheek Piece	135	Lr=110 Eb=23 Hc=1 Dr=1	Eb>Dr	0.7082	-1.2546	2.6592
		135		Hc>Dr	-0.6374	-3.4195	2.1759
		135		Lr>Dr	0.0073	-1.9222	1.9265

Table S18. Noseband type Bayesian Regression Model Results. Predictive/trend results are show in bold. (Cn=Cavesson noseband, Dn=Drop noseband, Fn=Flash noseband, Rn=Four-ring-drop noseband; D=Double Bridle; E=Elementary, M=Medium, A=Advanced Medium, P=Prix St. George, I=Intermediate I; R=Regionals, T=Typical; Mr=Male, Fr=Female; EB=Ear Bonnet, nEB=No Ear Bonnet; Lr=Loose ring, Eb=Eggbutt, Hc=Hanging cheek, Dr=D-ring).

Model	Variable Name	Sample Size	Category Size	Hypothesis	Estimate	Lower CrI	Upper CrI
Component 6 Noseband Model	**Noseband type**	114	Cn=62 Dn=3 Fn=44 Rn=5	Dn>Cn	-0.23	-1.88	1.43
		114		**Fn>Cn**	**-0.66**	**-1.29**	**-0.02**
		114		Rn>Cn	-0.62	-2.38	1.15
		114		Dn>Fn	0.43	-0.95	1.81
		114		Dn>Rn	0.39	-1.58	2.35
		114		Fn>Rn	-0.04	-1.5	1.42
	Average nose angle	114			0.05	-0.02	0.12
	**Competition level**	114	E=46 M=24 A=8 P=21 I=15	E>A	-0.12	-1.01	0.76
		114		I>A	0.15	-0.82	1.12
		114		M>A	-0.2	-1.11	0.69
		114		P>A	-0.61	-1.56	0.35
		114		E>I	-0.27	-0.89	0.35
		114		E>M	0.08	-0.42	0.57
		114		E>P	0.49	-0.07	1.05
		114		I>M	0.35	-0.3	1
		114		**I>P**	**0.76**	**0.14**	**1.37**
		114		M>P	0.41	-0.19	1.01
		114		R>T	-0.51	-1.25	0.23
	Rider gender	114	Fr=93 Mr=21	Mr>Fr	0.25	-0.33	0.81
	Ear Bonnet	114	nEB=61 EB=53	EB>nEB	-0.31	-0.75	0.12
	**Snaffle cheek piece**	114	Dr=1 Eb=16 Hc=1 Lr=96	Eb>Dr	-0.16	-2.34	1.99
		114		**Hc>Dr**	**2.52**	**-0.57**	**5.61**
		114		Lr>Dr	-0.13	-2.26	1.99
		114		**Eb>Hc**	**-2.67**	**-4.68**	**-0.67**
		114		Eb>Lr	-0.03	-0.58	0.51
		114		**Hc>Lr**	**2.64**	**0.69**	**4.59**
	Patting score	114			0	-0.01	0.02
	Test score	114			-0.01	-0.09	0.08
	Noseband*Average nose angle	114	Cn=62 Dn=3 Fn=44 Rn=5	Dn>Cn	0.04	-0.23	0.32
		114		Fn>Cn	-0.08	-0.19	0.02
		114		Rn>Cn	-0.16	-0.6	0.29
		114		Dn>Fn	0.12	-0.11	0.35
		114		Dn>Rn	0.2	-0.22	0.62
		114		Fn>Rn	0.08	-0.3	0.45
Component 7 Noseband Model	Noseband type	114	Cn=62 Dn=3 nF=44 Rn=5	Dn>Cn	-0.58	-2.26	1.09
		114		Fn>Cn	-0.29	-0.93	0.24
		114		Rn>Cn	0.29	-1.47	2.05
		114		Dn>Fn	-0.28	-1.67	1.09
		114		Dn>Rn	-0.86	-2.82	1.09
		114		Fn>Rn	-0.58	-2.02	0.87
	Average nose angle	114			-0.00	-0.08	0.07
	**Competition level**	114	E=46 M=24 A=8 P=21 I=15	E>A	0.28	-0.61	1.17
		114		I>A	0.2	-0.78	1.19
		114		M>A	0.07	-0.83	0.96
		114		P>A	-0.12	-1.08	0.84
		114		E>I	0.08	-0.54	0.7
		114		E>M	0.21	-0.28	0.7
		114		E>P	0.4	-0.15	0.96
		114		I>M	0.13	-0.53	0.8
		114		I>P	0.32	-0.29	0.93

		114		M>P	0.19	-0.4	0.8
		114		**R>T**	**-1.33**	**-2.07**	**-0.6**
	Rider gender	114	Fr=93 Mr=21	Mr>Fr	0.34	-0.24	0.92
	Ear Bonnet	114	nEB=61 EB=53	EB>nEB	0.13	-0.3	0.56
	Snaffle cheek piece	114	Dr=1 Eb=16	Eb>Dr	-0.23	-2.44	1.97
		114	Hc=1 Lr=96	Hc>Dr	0.12	-2.95	3.25
		114		Lr>Dr	0.11	-2.05	2.28
		114		Eb>Hc	-0.35	-2.34	1.6
		114		Eb>Lr	-0.34	-0.87	0.19
		114		Hc>Lr	0.01	-1.9	1.96
	Patting score	114			-0.01	-0.02	0.01
	Test score	114			-0.05	-0.13	0.04
	Noseband*Average	114	Cn=62 Dn=3	Dn>Cn	-0.02	-0.3	0.26
	nose angle	114	Fn=44 Rn=5	Fn>Cn	-0.03	-0.14	0.07
		114		Rn>Cn	0.13	-0.31	0.59
		114		Dn>Fn	0.01	-0.22	0.25
		114		Dn>Rn	-0.15	-0.57	0.27
		114		Fn>Rn	-0.17	-0.54	0.21
Component 4	**Noseband type**	114	Cn=62 Dn=3	Dn>Cn	0.77	-0.72	2.25
Noseband		114	Fn=44 Rn=5	**Fn>Cn**	**0.91**	**0.36**	**1.48**
Model		114		Rn>Cn	0.62	-0.95	2.21
		114		Dn>Fn	-0.15	-1.39	1.08
		114		Dn>Rn	0.15	-1.6	1.88
		114		Fn>Rn	0.3	-1.01	1.6
	Average nose angle	114			-0.04	-0.1	0.03
	**Competition level**	114	E=46 M=24 A=8	E>A	0.32	-0.47	1.11
		114	P=21 I=15	I>A	-0.07	-0.93	0.79
		114		M>A	-0.41	-1.21	0.38
		114		P>A	0.13	-0.72	0.97
		114		E>I	0.39	-0.15	0.93
		114		**E>M**	**0.73**	**0.29**	**1.16**
		114		E>P	0.19	-0.3	0.68
		114		I>M	0.34	-0.24	0.92
		114		I>P	-0.2	-0.74	0.34
		114		**M>P**	**-0.54**	**-1.07**	**0**
		114		**R>T**	**-1.38**	**-2.03**	**-0.72**
	Rider gender	114	Fr=93 Mr=21	Mr>Fr	-0.03	-0.54	0.47
	Ear Bonnet	114	nEB=61 EB=53	EB>nEB	-0.3	-0.67	0.07
	Snaffle cheek piece	114	Dr=1 Eb=16	Eb>Dr	-0.02	-1.93	1.9
		114	Hc=1 Lr=96	Hc>Dr	-1.02	-3.73	1.71
		114		Lr>Dr	0.01	-1.86	1.89
		114		Eb>Hc	1	-0.74	2.75
		114		Eb>Lr	-0.03	-0.51	0.44
		114		Hc>Lr	-1.03	-2.75	0.69
	Patting score	114			0.01	-0.00	0.03
	Test score	114			-0.03	-0.11	0.04
	**Noseband*Average**	114	C=62 D=3	Dn>Cn	0.05	-0.2	0.29
	**nose angle**	114	F=44 R=5	**Fn>Cn**	**0.13**	**0.03**	**0.22**
		114		Rn>Cn	0.02	-0.37	0.42
		114		Dn>Fn	-0.08	-0.29	0.12
		114		Dn>Rn	0.02	-0.35	0.39
		114		Fn>Rn	0.1	-0.23	0.43

6. Questionnaire

BD Dressage at Hartpury College Research Questionnaire

This is a questionnaire for competitors in BD Dressage competitions at Hartpury College to collect data for usage in an academic study. The study will not disclose any identifying features

of any horse and rider combination. All riders involved must be over the age of 18. To revoke consent for this to be used, please contact Rifka Faithfull at rio9@aber.ac.uk

1. The purpose of the collection of data in this questionnaire and video footage of your dressage test is for use in an academic study. The completed study will not disclose any names or identifiable features of the rider, all data will be anonymised, and no video footage will be released to the public.

   I give consent for my data to be used as specified above in this academic study. I understand that if I change my mind, I can contact rio9@aber.ac.uk to prevent my video and questionnaire answers being included in the research.

   ☐Yes ☐No
2. Please confirm that you are over the age of 18.

   For data protection purposes, all participants must be over the age of 18.

   ☐Yes ☐No

3. Please state competitor's name and competitor number.

   If you are competing more than one horse, please fill out one form for each horse.
   If the horse and rider combination are competing at multiple levels, please fill out one questionnaire but note questions that ask you to state an answer for each level competed at in this competition.

   ____

The Horse

1. What is the horse's age?

   ____

2. What is the horse's sex?

   ☐Mare ☐Gelding ☐Stallion
3. What is the horse's breed?

   ____

4. What is the horse's height (hands)?

   ____

5. When was the horse's saddle last fitted?

   ☐Saddle has not been professionally fitted
   ☐In the last week
   ☐In the last month
   ☐In the last 3 months
   ☐In the last 6 months
   ☐In the last year
   ☐More than a year ago
6. Has the horse's bridle been professionally fitted? If so, how long ago?

   ☐Bridle was not professionally fitted
   ☐In the last week
   ☐In the last month
   ☐In the last 3 months
   ☐In the last 6 months
   ☐In the last year

☐More than a year ago

7. What type of bit is the horse ridden in for dressage?

   If this is different for dressage training and competitions, please state both bits and whether they are for training or competition.

   ____

8. If the horse is being ridden in a double bridle at this competition, how long has the horse been ridden in a double bridle?

   ☐Not ridden in a double bridle at this competition
   ☐Less than a month
   ☐Less than 3 months
   ☐Less than 6 months
   ☐Less than a year
   ☐Less than 2 years
   ☐Less than 5 years
   ☐More than 5 years

9. If the horse is being ridden in a double bridle at this competition, how often are they ridden in a double bridle at home?

   ☐ Only at competition
   ☐ Rarely used at home
   ☐ Used for less than half of training rides
   ☐ Used for most training rides
   ☐ Always trained in a double bridle

10. Does the horse have regular maintenance treatment? Which treatment, and how frequently?

    Please tick each treatment and how regularly they each occur. If they do not receive a treatment, tick the N/A column.

	N/A	Weekly	Biweekly	Monthly	3 Monthly	6 Monthly	Yearly
Physiotherapist	☐	☐	☐	☐	☐	☐	☐
Massage	☐	☐	☐	☐	☐	☐	☐
Osteopath	☐	☐	☐	☐	☐	☐	☐
Hydrotherapy	☐	☐	☐	☐	☐	☐	☐
Chiropractor	☐	☐	☐	☐	☐	☐	☐
Cryotherapy	☐	☐	☐	☐	☐	☐	☐
Ultrasound Therapy	☐	☐	☐	☐	☐	☐	☐
Laser Therapy	☐	☐	☐	☐	☐	☐	☐
Red Light Therapy	☐	☐	☐	☐	☐	☐	☐
Acupuncture	☐	☐	☐	☐	☐	☐	☐
Magnetic Therapy	☐	☐	☐	☐	☐	☐	☐

11. Please state any other maintenance treatments the horse receives that are not listed in the previous question and how frequently these occur. If no additional treatments, answer none.

    For example: Physiotherapy - every 6 weeks etc.

    ____

12. How long ago were the horse's teeth last checked?

    ☐In the last week
    ☐In the last month

☐In the last 3 months
☐In the last 6 months
☐In the last year
☐In last 2 years
☐More than 2 years ago

13. How frequently are the horse's teeth checked?

____

14. How recently has the horse seen a farrier?

☐In the last week
☐In the last 2 weeks
☐In the last month
☐In the last 3 months
☐More than 3 months ago

15. How frequently does the horse see the farrier?

____

16. Does the horse normally become stressed at competitions?

☐No
☐The horse experiences low stress levels at competitions
☐The horse experiences moderate stress levels at competitions
☐The horse experiences high stress levels at competitions

Current Injuries and Health conditions

This section is only in reference to injuries/health conditions the horse is **currently experiencing and currently being treated for.** For each applicable condition, please rate its severity on the scale below. The questions use the following severity scale:

1– Requires no medical intervention
2– Requires low level pain management
(e.g. horse continues training, possibly at a lower intensity but requires half or one sachet of Equipalazone (bute))
3– Requires time off training and potentially box rest for up to a week.
Alternatively training proceeds as usual, but the horse requires veterinary intervention on a regular basis to maintain pain free, comfortable training (for example 6 monthly injections for arthritic horses)
4– Requires veterinary intervention and box rest for up to a month
5– Requires serious veterinary intervention (e.g. surgery) and/or intensive nursing for recovery and requires more than a month of time off training and box rest

1. Is the horse lame?

☐No ☐1 ☐2 ☐3 ☐4 ☐5

2. Do they have arthritis?

☐No ☐1 ☐2 ☐3 ☐4 ☐5

3. Are they laminitic?

☐No ☐1 ☐2 ☐3 ☐4 ☐5

4. Do they have colic?

☐No ☐1 ☐2 ☐3 ☐4 ☐5

5. Do they have respiratory issues?

☐No ☐1 ☐2 ☐3 ☐4 ☐5

6. Do they have a skin condition?
   ☐No ☐1 ☐2 ☐3 ☐4 ☐5
7. Do they have gastric ulcers?
   ☐No ☐1 ☐2 ☐3 ☐4 ☐5
8. Do they have a ligament or tendon injury that is currently being treated?
   ☐No ☐1 ☐2 ☐3 ☐4 ☐5
9. Do they currently have dental issues?
   ☐No ☐1 ☐2 ☐3 ☐4 ☐5
10. Please state any other current health conditions and their severity.
   ☐No ☐1 ☐2 ☐3 ☐4 ☐5

Previous Injuries and Health conditions

This section is only in reference to any **previous** injuries or health conditions that **have been treated and the horse is now recovered** from. For each applicable condition, please rate its severity on the scale below. The questions use the following severity scale:

1– Required no medical intervention
2– Required low level pain management
(e.g. horse continues training, possibly at a lower intensity but requires half or one sachet of Equipalazone (bute))
3– Required time off training and potentially box rest for up to a week.
Alternatively, training proceeded as usual, but the horse required veterinary intervention on a regular basis to maintain pain free, comfortable training (for example 6 monthly injections for arthritic horses)
4– Required veterinary intervention and box rest for up to a month
5– Required serious veterinary intervention (e.g. surgery) and/or intensive nursing for recovery and required more than a month of time off training and box rest

11. Has the horse previously been lame?
   ☐No ☐1 ☐2 ☐3 ☐4 ☐5
12. Have they previously had arthritis that they are now recovered from?
   ☐No ☐1 ☐2 ☐3 ☐4 ☐5
13. Have they had laminitis that they are now recovered from?
   ☐No ☐1 ☐2 ☐3 ☐4 ☐5
14. Have they previously had colic?
   ☐No ☐1 ☐2 ☐3 ☐4 ☐5
15. Have they previously had respiratory issues that have now been treated?
   ☐No ☐1 ☐2 ☐3 ☐4 ☐5
16. Have they previously had a skin condition that they have now recovered from?
   ☐No ☐1 ☐2 ☐3 ☐4 ☐5
17. Have they previously had gastric ulcers that they are now recovered?
   ☐No ☐1 ☐2 ☐3 ☐4 ☐5
18. Have they previously had a ligament or tendon injury that they have recovered from?
   ☐No ☐1 ☐2 ☐3 ☐4 ☐5
19. Have they previously had dental issues that have now been treated?
   ☐No ☐1 ☐2 ☐3 ☐4 ☐5
20. Please state any other previous health conditions and their severity.

☐No ☐1 ☐2 ☐3 ☐4 ☐5

____

The Rider

1. What is the rider's age?

   ____
2. What is the rider's height (cm/feet and inches)?

   ____
3. What is the rider's weight (kg/lb/st)? (This question is optional)

   ____
4. Does the rider have regular maintenance-treatment (e.g. physiotherapist, sports massage, osteopath etc.)? Please state each treatment and how regularly they each occur.

   For example: Sports massage – monthly

   ____

Competition and Training Experience

This section asks questions about what level the horse and the rider have each trained and competed up to. The levels competed to include both unaffiliated and affiliated competition. For example, if the rider competed at elementary unaffiliated for a year and then another year affiliated, they have competed at elementary for 2 years.

1. At what level is the combination competing at in this competition?
   - ☐Elementary
   - ☐Medium
   - ☐Advanced Medium
   - ☐Advanced
   - ☐Prix St George
   - ☐Intermediate I
   - ☐Intermediate II
   - ☐Grand Prix
2. What is the highest level the rider competed to?
   - ☐Elementary
   - ☐Medium
   - ☐Advanced Medium
   - ☐Advanced
   - ☐Prix St George
   - ☐Intermediate I
   - ☐Intermediate II
   - ☐Grand Prix
3. How long have they competed at that level?
   - ☐Less than a month
   - ☐Less than 3 months
   - ☐Less than 6 months
   - ☐Less than a year
   - ☐Less than 2 years
   - ☐Less than 5 years

☐More than 5 years

4. What is the highest level the rider competently trained at home?
   ☐Elementary
   ☐Medium
   ☐Advanced Medium
   ☐Advanced
   ☐Prix St George
   ☐Intermediate I
   ☐Intermediate II
   ☐Grand Prix
5. What is the highest level the horse competed to?
   ☐Elementary
   ☐Medium
   ☐Advanced Medium
   ☐Advanced
   ☐Prix St George
   ☐Intermediate I
   ☐Intermediate II
   ☐Grand Prix
6. How long have they competed at that level?
   ☐Less than a month
   ☐Less than 3 months
   ☐Less than 6 months
   ☐Less than a year
   ☐Less than 2 years
   ☐Less than 5 years
   ☐More than 5 years
7. What is the highest level the horse competently trained at home?
   ☐Elementary
   ☐Medium
   ☐Advanced Medium
   ☐Advanced
   ☐Prix St George
   ☐Intermediate I
   ☐Intermediate II
   ☐Grand Prix
8. To what level was the horse trained before it started competing?
   ☐Introductory
   ☐Preliminary
   ☐Novice
   ☐Elementary
   ☐Medium
   ☐Advanced Medium
   ☐Advanced
   ☐Prix St George
   ☐Intermediate I
   ☐Intermediate II

☐Grand Prix

9. How long did it spend training before it started competing?

   ☐Less than 6 months
   ☐Less than a year
   ☐Less than 2 years
   ☐Less than 5 years
   ☐More than 5 years

10. Please state how long the horse both trained and competed at the level of dressage it is entered at in this competition.

    For example: Preliminary - trained for 2 years, competed for 1 year

    If the horse is competing at multiple levels at this competition, please answer for each level.

    ____

11. Please state how long the rider both trained and competed at the level of dressage they are entered at in this competition.

    For example: Preliminary - trained for 2 years, competed for 1 year

    If the horse is competing at multiple levels at this competition, please answer for each level.

    ____

12. Please state how long the horse and rider combination both trained and competed together at the level of dressage they are entered at in this competition.

    For example: Preliminary - trained for 2 years, competed for 1 year

    If the horse is competing at multiple levels at this competition, please answer for each level.

    ____

7. References

1. Górecka-Bruzda, A.; Kosińska, I.; Jaworski, Z.; Jezierski, T.; Murphy, J. Conflict Behavior in Elite Show Jumping and Dressage Horses. *Journal of Veterinary Behavior* **2015**, *10*, 137–146, doi:10.1016/j.jveb.2014.10.004.
2. Mellor, D.J. Mouth Pain in Horses: Physiological Foundations, Behavioural Indices, Welfare Implications, and a Suggested Solution. *Animals* **2020**, *10*, 572, doi:10.3390/ani10040572.
3. Anttila, M.; Raekallio, M.; Valros, A. Oral Dimensions Related to Bit Size in Adult Horses and Ponies. *Front. Vet. Sci.* **2022**, *9*, doi:10.3389/fvets.2022.879048.
4. Økken, C.B. Factors Affecting the Behaviour of Horses When Ridden, with Emphasis on Bridles and Other Equipment Used to Control Behaviour. Master thesis, Norwegian University of Life Sciences, Ås, 2020.
5. Clayton, H.M. A Fluoroscopic Study of the Position and Action of Different Bits in the Horse's Mouth. *Journal of Equine Veterinary Science* **1985**, *5*, 68–77, doi:10.1016/S0737-0806(85)80050-2.
6. Cross, G.H.; Cheung, M.K.P.; Honey, T.J.; Pau, M.K.; Senior, K.-J. Application of a Dual Force Sensor System to Characterize the Intrinsic Operation of Horse Bridles and Bits. *Journal of Equine Veterinary Science* **2017**, *48*, 129-135.e3, doi:10.1016/j.jevs.2016.01.017.
7. Karl, P. *Dérives Du Dressage Moderne : - Recherche d'une Alternative "Classique";* Equitation; Editions Belin: Paris, 2006; ISBN 978-2-7011-4450-4.

8. Bennett, D.G. An Overview of Bits and Bitting. In Proceedings of the Focus on Dentistry Meeting; 2006.
9. Christensen, J.W.; Keeling, L.J.; Nielsen, B.L. Responses of Horses to Novel Visual, Olfactory and Auditory Stimuli. *Applied Animal Behaviour Science* **2005**, *93*, 53–65, doi:10.1016/j.applanim.2005.06.017.
10. Eisersiö, M.; Roepstorff, L.; Weishaupt, M.A.; Egenvall, A. Movements of the Horse's Mouth in Relation to Horse–Rider Kinematic Variables. *The Veterinary Journal* **2013**, *198*, e33–e38, doi:10.1016/j.tvjl.2013.09.030.
11. Witzmann, P. DAS PFERDEMAUL – EIN DUNKLER, UNBEKANNTER ORT?; January 15 2011.
12. Cook, W.R.; Kibler, M. Behavioural Assessment of Pain in 66 Horses, with and without a Bit. *Equine Veterinary Education* **2019**, *31*, 551–560, doi:10.1111/eve.12916.
13. Elbrønd, V.; Schultz, R. Myofascial Kinetic Lines in Horses. *Equine Veterinary Journal* **2014**, *46*, 40–40, doi:10.1111/evj.12267_121.
14. Scoggins, R.D. Bits, Bitting, and Dentistry. **2001**.
15. McLean, A.N.; McGreevy, P.D. Horse-Training Techniques That May Defy the Principles of Learning Theory and Compromise Welfare. *Journal of Veterinary Behavior* **2010**, *5*, 187–195, doi:10.1016/j.jveb.2010.04.002.
16. Johnson, T.J.; Porter, C.M. Dental Conditions Affecting the Mature Performance Horse (5-15 Years). In Proceedings of the American Association of Equine Practitioners; 2006; pp. 17–25.
17. McGreevy, P.; Warren-Smith, A.; Guisard, Y. The Effect of Double Bridles and Jaw-Clamping Crank Nosebands on Temperature of Eyes and Facial Skin of Horses. *Journal of Veterinary Behavior* **2012**, *7*, 142–148, doi:10.1016/j.jveb.2011.08.001.
18. Werhahn, H.; Hessel, E.F.; Schulze, H.; Van den Weghe, H.F.A. Temporary Turnout for Free Exercise in Groups: Effects on the Behavior of Competition Horses Housed in Single Stalls. *Journal of Equine Veterinary Science* **2011**, *31*, 417–425, doi:10.1016/j.jevs.2011.01.006.
19. McBride, S.; Hemmings, A. A Neurologic Perspective of Equine Stereotypy. *Journal of Equine Veterinary Science* **2009**, *29*, 10–16, doi:10.1016/j.jevs.2008.11.008.
20. Eggbut Cheekpieces vs Loose Rings Available online: https://www.fei.org/stories/lifestyle/teach-me/eggbut-cheekpieces-vs-loose-rings (accessed on 27 February 2025).
21. Barrey, E.; Desliens, F.; Poirel, D.; Biau, S.; Lemaire, S.; Rivero, J.-L.L.; Langlois, B. Early Evaluation of Dressage Ability in Different Breeds. *Equine Veterinary Journal* **2002**, *34*, 319–324, doi:10.1111/j.2042-3306.2002.tb05440.x.
22. Dyson, S.; Martin, C.; Bondi, A.; Ellis, A.D. The Influence of Rider Skill on Ridden Horse Behaviour, Assessed Using the Ridden Horse Pain Ethogram, and Gait Quality. *Equine Veterinary Education* **2022**, *34*, e308–e317, doi:10.1111/eve.13434.
23. Moine, S.; Flammer, S.A.; de Jesus Maia-Nussbaumer, P.; Klopfenstein Bregger, M.D.; Gerber, V. Evaluation of the Effects of Performance Dentistry on Equine Rideability: A Randomized, Blinded, Controlled Trial. *Veterinary Quarterly* **2017**, *37*, 195–199, doi:10.1080/01652176.2017.1329598.
24. Carmalt, J.L.; Carmalt, K.P.; Barber, S.M. The Effect of Occlusal Equilibration on Sport Horse Performance. *J Vet Dent* **2006**, *23*, 226–230, doi:10.1177/089875640602300405.
25. Pehkonen, J.; Karma, L.; Raekallio, M. Behavioral Signs Associated With Equine Periapical Infection in Cheek Teeth. *Journal of Equine Veterinary Science* **2019**, *77*, 144–150, doi:10.1016/j.jevs.2019.03.005.
